# Simultaneous delayed fluorescence and phosphorescence in organic luminescent material employing multiple excited states

**DOI:** 10.1038/s41377-025-02063-x

**Published:** 2026-01-01

**Authors:** Dehai Dou, Wenlan Liu, Xin Zhou, Qiqi Yang, Xiao Tan, Naz Ugur, Chongyao Li, Charusheela Ramanan, Xiaomin Liu, Gert-Jan A. H. Wetzelaer, Denis Andrienko, Martin Baumgarten, Paul W. M. Blom, Yungui Li

**Affiliations:** Max Planck Institute for Polymer Research, Mainz, Germany

**Keywords:** Organic LEDs, Polymers

## Abstract

Triplet dynamics play a key role in room temperature phosphorescence (RTP) and thermally activated delayed fluorescence (TADF). In this work, we report a model emitter with three emission components: prompt fluorescence (PF) in nanoseconds, delayed fluorescence in microseconds, and RTP in milliseconds, with the emission spectrum ranging from ultraviolet to deep blue. We experimentally and theoretically verify that a second triplet excited state, T_2_, below the singlet state S_1_ is involved in facilitating simultaneous PF, TADF, and RTP in the model emitter. The reverse intersystem crossing (rISC) from T_2_ to S_1_ contributes to the TADF, while the radiative transition from T_1_ to the ground state is the origin of the long-lived RTP. By transferring the energy of multiple excited states to a series of conventional fluorescence emitters, a multi-color emissive system covering the entire visible wavelength range has been realized, with the photoluminescence decay ranging from 10^−9^ s to 10^−1^ s. By slightly tuning the energy difference between these excited states in the model molecule, a highly efficient organic luminescent material with only PF and RTP emission has been obtained with an RTP quantum yield above 30%. This work provides insights into the key role of higher-lying triplet states in the development of efficient TADF and RTP materials.

## Graphical abstract

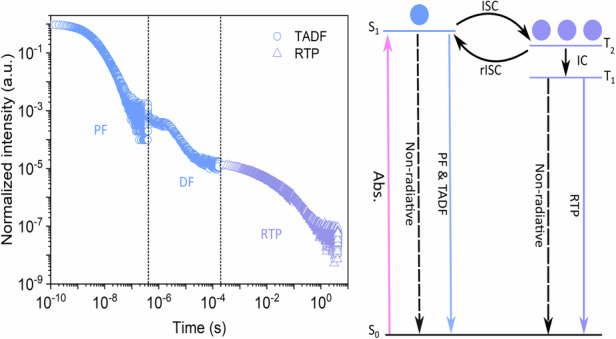

## Introduction

Exciton kinetics have a significant impact on the efficiency and operational stability of optoelectronic devices based on organic semiconductors^1–3^. In purely organic emitters, triplets are normally non-radiative or have low radiative rates, because the transition from triplets to the ground state is spin forbidden. However, 75% of excitons formed through electron-hole recombination in electrically driven light-emitting devices are triplets^4^. Mainly two strategies have been developed to harvest these non-radiative triplets for efficient organic light-emitting diodes (OLEDs). By using the heavy atom effect from iridium or platinum with a high atomic number, the intersystem crossing (ISC) and triplet radiation have been significantly enhanced, giving rise to efficient phosphorescence at room temperature^5–7^. Another strategy is minimizing the energy splitting between the singlet and triplet states Δ*E*_ST_. With the assistance of thermal energy (~0.026 eV) at room temperature, TADF emission from organic emitters with singlet-triplet splitting gaps within the thermal energy to even ~0.2 eV has been observed^8–10^. Recently, there have also been reports of direct triplet emission in purely organic emitters as room temperature phosphorescence (RTP), achieved by fixing organic emitters in a polymer matrix or crystals to reduce non-radiative relaxation from triplets^11–14^. Because there is no need for rare and expensive heavy metal ingredients, TADF and RTP emissions from purely organic emitters have gained tremendous scientific attention in recent years^12,15–17^.

For purely organic emitters, the prompt fluorescence (PF) originating from the spin-allowed singlet radiative transition has an emission lifetime on the nanoseconds timescale, resulting in a typical singlet radiative rate *k*_r,S_ in the range of 10^8^–10^10^ s^−1^^18–20^. The TADF from the ISC-rISC cycling typically has a longer decay lifetime of 10^−7^–10^−4^ s, because of the involvement of spin-flip processes and nuclear geometry changes between the molecular configuration of the singlet and triplet states^9,21^. In the case of RTP, the direct triplet radiation is very slow, with rates in the range of 1–10^3^ s^−1^^11,12,22,23^. These are typical rate constants for purely organic emitters, however, it is possible that the observed fluorescence, TADF and phosphorescence decay rates can also vary greatly outside these ranges for specific systems. Nevertheless, the transient emission behavior of purely organic emitters is kinetically determined by the involved processes in a coupled manner^8,21,24^. Because the kinetic rates related to TADF and RTP differ by several orders of magnitude, a comprehensive photophysical study of excitonic processes involved in these systems is of scientific importance^25–28^.

The transient photophysical properties for TADF and RTP emitters have been widely investigated and are typically modelled with a three-level model, in which two excited states – a singlet S_1_ and a triplet state T_1_-are involved^11,24^. The involvement of a second triplet state, T_2_, energetically above T_1_ and below S_1_ has been theoretically considered and discussed^29–31^. However, direct experimental verification of higher-lying triplet states in TADF and/or RTP systems is challenging. Therefore, from the experimental perspective, a thorough understanding of the impact of the higher-lying triplet state on the photophysical properties is still needed. For organic emitters, the rate of the (reverse) ISC process is dependent on the strength of the spin-orbit coupling (SOC) between singlet and triplet states, and also the energy difference between these states^18^. The coupling strength of SOCs largely rely on the orbital nature of involved states, which can be impacted by increasing the effective nuclear charge at the nucleus, and also the spin and orbital angular momenta of involved electrons^19^. Considering the smaller energy difference S_1_-T_2_ as compared to S_1_-T_1_, the rISC rate between S_1_-T_2_ can be potentially higher than the rate between S_1_-T_1_ if the orbital nature for T_2_ and T_1_ is similar. For TADF emitter design, a faster rISC rate will reduce the triplet concentration under a constant current density, which will enhance the operational stability for electroluminescent devices based on TADF emitters^32^. Furthermore, assuming similar orbital properties for T_2_ and T_1_, according to Fermi’s golden rule, a higher ISC rate is anticipated between S_1_-T_2_, as compared to S_1_-T_1_, because of the relatively smaller energy difference. If T_1_ is stabilized and the internal conversion between T_2_ and T_1_ is fast, efficient RTP emitters can still be obtained in cases that T_2_ and T_1_ are isoenergetic^18^. Therefore, the experimental investigation of the higher-lying triplet state and its impact on photophysical properties is of general significance for organic luminescent materials design and as well as for the development of electroluminescent devices.

In this work, we show that with only two excited states S_1_ and T_1_, it is impossible to achieve multiple emissive components in a transient PL decay: PF in nanoseconds (ns), TADF in microseconds (μs), and RTP in milliseconds (ms) from a single emissive system. In the present study, we firstly report a model emitter 3,6-di-tert-butyl-1,8-bis(3-(4,6-diphenyl-1,3,5-triazin-2-yl)phenyl)-9-phenyl-9H-carbazole (1.8-mDTAZ-PhtCz), with photoluminescence (PL) signatures of PF, TADF and RTP decay. Using the emitter 1.8-mDTAZ-PhtCz as a model system, we experimentally and theoretically verify by transient photoluminescence, transient absorption (TA) spectroscopy together with molecular simulations that a higher-lying triplet state T_2_ is involved. The transient emissive behavior can be described and fitted with a four-level model, with the upper triplet state T_2_ contributing to the TADF property while the T_1_ state being responsible for the RTP emission. Based on the model emitter, color tunable systems have been realized via Förster resonance energy transfer (FRET) to conventional fluorescent emitters, with the fluorescence decay spanning from the nanosecond to second region. By further modifying the chemical structure of the model molecule, one derivative named as 3,6-di-tert-butyl-1,8-bis(4-(4,6-diphenyl-1,3,5-triazin-2-yl)phenyl)-9-phenyl-9H-carbazole (1.8-pDTAZ-PhtCz) has been obtained. This new derivative exhibits highly efficient PF/RTP emission, with the RTP quantum yield above 30%.

## Results

### Synthesis and basic characterization

**Fig. 1:**
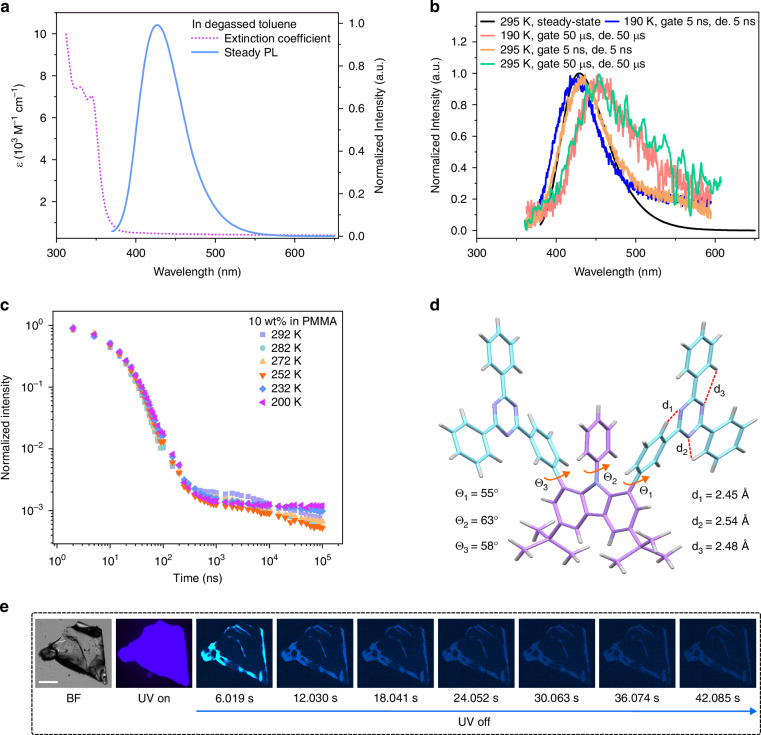
Luminescent materials with higher-lying triplet state. **a** Steady-state UV-Vis absorption and photoluminescence (PL) emission spectra obtained in degassed toluene at room temperature. **b** Time-resolved PL spectra. The transient PL were obtained with the sample being excited with a laser excitation at 360 nm in a closed cryostat at different temperatures, with the delay and gate time indicated. The steady state PL spectrum was measured under ambient condition. The doping concentration of the model emitter in PMMA is 2 wt%. **c** Temperature dependent transient PL decay of 1.8-mDTAZ-PhtCz (10 wt%) doped in PMMA. Here, the PL decay was measured by an iCCD camera, with the measurement condition summarized in the experimental section. **d** Intramolecular interactions of the model compound in the single crystal. **e** Ultralong afterglow emission from the model emitter 1.8 mDTAZ-PhtCz crystal

The model emitter (1.8-mDTAZ-PhtCz) and its derivative (1.8-pDTAZ-PhtCz) consists of two triazines (TAZ) as the acceptor units and a di-tert-butylcarbazole as the donor unit. The donor and acceptor moieties are attached to the bridging benzene rings in the *meta* or *para*-position. The synthetic route is summarized in Scheme S1. The structures of intermediates and the target product are unambiguously characterized by nuclear magnetic resonance (NMR) spectra (Fig. S1–S8), high-resolution mass spectra (HRMS), high performance liquid chromatography (HPLC), and finally by X-ray diffraction analysis from suitable crystals. The single crystal structure for the model emitter is shown in Fig. 1d, respectively.

The extinction coefficient of 1.8-mDTAZ-PhtCz in toluene obtained from UV-Vis absorption spectroscopy is shown in Fig. 1a. The strong absorption band at 330-360 nm is attributed to the π-π∗ transitions^33^. Strong PL emission from the emitter is observed, spanning from the ultraviolet to deep blue region, presented in Fig. 1a, and Figs. S9, S10. The fluorescence is solvent polarity dependent, as summarized in Table 1 and Table S1, indicating the charge transfer (CT) nature of the emissive dipoles^34^. The absolute photoluminescence quantum yield (PLQY) of the model emitter 1.8 mDTAZ-PhtCz in degassed toluene is 46.2%. When highly diluted in a rigid polymer matrix (2 wt%), it is 36.1% in air and 51.1% in nitrogen, respectively. The difference of PLQY in ambient and nitrogen atmosphere indicates the involvement of triplets during the emissive process. The key photophysical properties are summarized in Table 1. The delayed PL spectrum at room temperature spans from the ultraviolet to the deep blue region as well, shown in Fig. 1b, similar to that obtained at 190 K.

**Table 1:** Key photophysical properties for developed luminescent materials

Sample	λ_Abs_ (nm)^a^	λ_FL_ (nm)^a^	λ_FL_ (nm)^b^	λ_PH_ (nm)^c^	*φ* (%)^a^	*φ*_PF_ (%)^d^	*φ*_DF+PH_ (%)^d^	*φ*_PH_ (%)^d^
1.8-mDTAZ-PhtCz	330/345	425	427	458	46.2	36.1	15	e
1.8-pDTAZ-PhtCz	344/362	430	441	523	87.8	56.0	f	33.6

^a^In toluene

^b^Steady-state PL measured at ambient condition, doped in PMMA with 2 wt%

^c^Delayed spectra measured at room temperature, doped in PMMA with 2 wt%

^d^Doped in polystyrene (PS) with 2 wt%

^e^RTP quantum yield mixed with DF emission

^f^RTP without DF emission

A detailed temperature-dependent time-resolved PL study is conducted on the transient PL behavior of the model emitter 1.8-mDTAZ-PhtCz in PMMA. As shown in Fig. 1c, an intensity decrease of the delayed fluorescence is observed from 292 K to 252 K, consistent with the feature of TADF. Further reducing the temperature to 232 K, the intensity of the delayed component increases. At even lower temperature at 77 K (Fig. S9d), there is only a long-lived PL component in the delayed time regime, indicating that the phosphorescence emission gradually dominates at low temperatures. The decay lifetime for the PF, DF and RTP of the model emitter in PMMA at room temperature is estimated as 6.47 ns, 2.51 μs and 84.62 ms, respectively, as shown in Fig. S11 and Table S2. As shown in Fig. S12 and Table S3, PF, TADF, and RTP can still be observed at a lower doping concentration (0.5 wt% in PMMA), with decay lifetimes comparable to those at 2 wt%. These results suggest that the three-component PL emission is intrinsic, and it is less likely due to emitters with different molecular configurations or aggregated species (e.g., dimers/trimers).

The difference of PL decay of 1.8-mDTAZ-PhtCz in PS and PMMA is very minor, as shown in Fig. S13 and Table S4. This is reasonable because the change of RTP decay observed in previous reports were either from external heavy atom effects or molecular packing^35^. In our case, the polymeric host PS or PMMA has no heavy atom effect. The slight difference of molecular rigidity or polarity can only give a minor effect on the decay kinetics. The transient PL decay for the model emitter in degassed toluene is shown in Fig. S9. At room temperature, it is noted that the PL emission within the millisecond region part is quenched in degassed toluene, while clear PF and DF signals are still observed. Therefore, the presence of the delayed fluorescence and phosphorescence emission from the model emitter has been confirmed by the systematic transient PL measurements in different conditions.

The singlet and triplet energy can be determined by the emissive peak or lineshape analysis from the fluorescence and delayed spectrum. From the emissive peaks, the singlet and triplet levels of the model emitter is 2.904 eV and 2.707 eV, respectively. The lineshape analysis is based on the consideration that vibronic states with a small energy difference in the ground state contribute to the luminescent spectra from organic emitters. The Franck−Condon factors describing the transition probabilities to different vibronic states (i.e., different Gaussian shape fitting components) follow a Poisson progression, which leads to the relation between the emission spectral lineshape and energetic states involved, as shown in Fig. S16^36^. Such a lineshape analysis has been used to determine energy levels for TADF and RTP emitters^11,37^. As shown in Fig. S17, the 0-0 bands transition energy *E*_00_ is as 2.985 eV and 2.945 eV for the model emitter S_1_ and T_1_, respectively. The other related physical parameters obtained from the lineshape analysis are summarized in Table S6.

As shown in Fig. 1e, after stopping the UV irradiation, the intensity of the photoluminescence gradually decreases and remains present up to 42 s, indicating ultra-long afterglow phosphorescence emission in 1.8-mDTAZ-PhtCZ crystals at room temperature.

The long-lived PL of the 1.8-mDTAZ-PhtCz crystal is related to the packing mode. The crystal structure of 1.8-mDTAZ-PhtCz is shown in Fig. 1d and Fig. S18. In the single crystal, the TAZ groups are packed in a relative plane configuration, which might result from the strong intramolecular confinement from hydrogen bonds (2.48–2.54 Å), steric effects in crystal structures and/or intermolecular π- π staking. Meanwhile, the torsion angle Θ_2_ between the *N*-phenyl ring and the tert-butylcarbazole is 63°, close to the torsion angles between phenyl-triazine and tert-butylcarbazole (Θ_1_: 55°, Θ_3_: 58°) (Fig. 1d). Such a twisted configuration leads to a distortion between the donor and acceptor moieties. The intermolecular packing in the single crystal is presented in Fig. S18. The triazine planes from the two molecules are packed with a vertical distance of 3.35 Å, with a shift angle of 48.79°. Molecules are packed in a monoclinic style, with the detailed crystal parameters summarized in Table S7. These weak intramolecular and intermolecular interactions in crystals significantly reduce the molecular movements, giving rise to the long-lived triplet emission in crystals^38^.

### Limitation of the three-level model

We further turn to study the exciton kinetics of the model emitter 1.8-mDTAZ-PhtCz. Pronounced PF decay, TADF and RTP emissions from the nanosecond to second region have been observed by time-correlated single photon counting (TCSPC) measurements, which will be discussed in the following section. The transient PL kinetics of purely organic emitters with PF and TADF emission has been modeled with a three-level model system, with only a singlet excited state S_1_, a triplet state T_1_ and the ground state^24,39^. Analytically, the kinetics of singlets and triplets in the three-level system can be described by a bi-exponential function with two different decay lifetimes^31,40^. Theoretically, for a three-level system, the maximum number of transient PL components with different decay lifetimes is two, though PF, TADF and phosphorescence can be co-existed. When both TADF and phosphorescence contributes to the PL emission, the decay lifetime of TADF and phosphorescence should be at the same order of magnitude, otherwise the faster process will suppress the slower one. Therefore, it is impossible to observe transient decay with lifetimes in nanosecond, microsecond and millisecond from a three-level system described by Scheme S2.

To more intuitively understand the exciton dynamics described by a three-level model, the singlet and triplet evolution under different assumed kinetic rates are numerically simulated, as shown in Figs. S21, S22 in the Supplementary note S1. In summary, only two components with different orders of decay lifetime can be resolved with a three-level model. One can interpret the numerical results in the following way. To maintain the long-lived RTP emission in ms, the intrinsic triplet lifetime τ_T_ in the model should be comparable to that value. Meanwhile, to obtain delayed fluorescence with lifetime in μs, the rate *k*_rISC_ should be in the range of 10^6^ s^−1^. Since the rISC and RTP come from the same source T_1_ in the three-level model, if *k*_rISC_ is orders of magnitude higher than the triplet radiative rate (~1/τ_T_), triplets will mainly convert to singlets via rISC, leading to vanishing of RTP with pronounced TADF emission. In the other case when *k*_rISC_ is of the same order of 1/τ_T_, only one delayed component can be observed with a lifetime comparable to τ_T_, though both TADF and RTP contribute to the delayed PL. Finally, if *k*_rISC_ is orders lower than 1/τ_T_, only the long-lived RTP can be resolved, while the TADF emission is negligible. This is the normal case for RTP emitters when Δ*E*_ST_ is much larger than the thermal energy. The change of *k*_ISC_ and τ_S_ can only change the PF lifetime and the intensity of the delayed PL emission. Therefore, based on the three-level model, the triplet state can only contribute to one delayed PL signal, though the lifetime of the delayed component can be varied over several orders of magnitude. It is impossible to obtain two delayed components with lifetimes in the μs and ms simultaneously, with only one triplet state.

### Involvement of upper triplet state in the model emitter

**Fig. 2:**
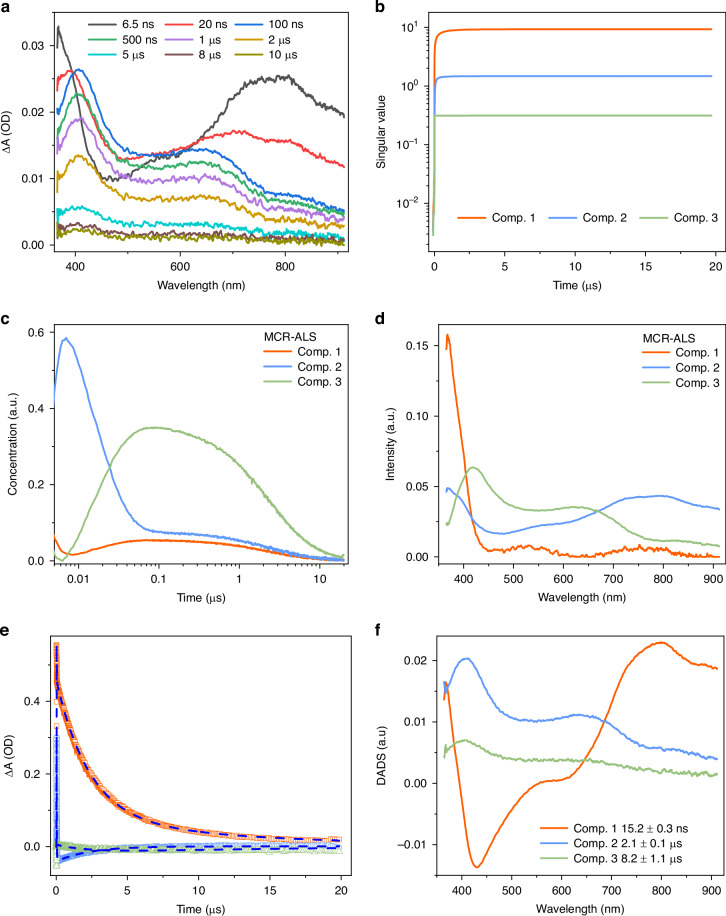
Characterization of excited states kinetics by ns-TA spectroscopy in degassed toluene at room temperature. **a** Experimental ns-TA spectra at specific delay times in the UV-Vis range at room temperature. **b** Forward EFA of the ns-TA data. **c** Concentration evolution and (**d**) associated spectra of the principal components from MCR-ALS fitting. **e** Kinetic fitting by the global analysis with three components based on a parallel scheme. **f** DADS from the global analysis

To reveal the mechanism behind the simultaneous occurrence of TADF and RTP, we then use TA spectroscopy to monitor the excited state kinetics in the model emitter 1.8-mDTAZ-PhtCz. The ns-TA spectra in degassed solution are shown in Fig. 2a within the UV-Vis wavelength range. The 2D ns-TA spectra of 1.8-mDTAZ-PhtCz in degassed and aerated toluene are shown in Fig. S33. A pronounced long-lived excited state absorption (ESA) signal from the nanosecond to microsecond timescale is observed.

A forward evolving factor analysis (EFA) based on the UV-Vis ns-TA dataset indicates that there are three principal components contributing to the ns-TA signal (Fig. 2b)^41,42^. We therefore use three components to fit the ns-TA data by the multivariate curve resolution alternating least square (MCR-ALS) algorithm, in which the experimental dataset is decomposed into associated spectra and concentration profiles of the principal components^43^. In this soft-hard modelling process, the only constraint condition is that the concentration for each component is non-negative, which is physically robust. The decomposed concentration profiles and associated spectra are shown in Fig. 2c and Fig. 2d, respectively. From the concentration evolution, component 2 has a fast decay within the early ~50 ns, while the other two components firstly grow in intensity, followed by a gradual concentration decrease in the microsecond region. The associated spectra for these components indicate the absorptive feature of these components in the UV-Vis range.

A global analysis based on the parallel model is then carried out to further uncover the physical origin of these components^44^. As shown in Fig. 2e, the experimental kinetic decays can be fitted nicely with three components within the investigated timescale. The decay associated difference spectra (DADS) for these components are presented in Fig. 2f. DADS1 exhibits ESA peaks at <400 nm and 700–900 nm, with a decay lifetime about 15 ns, indicating that it is a singlet state. This transient spectral change corresponds to the fast signal change within the first ~100 ns shown in Fig. 2a. DADS2 has ESA peaks at 400 nm and 700 nm, with a decay lifetime of ~2.1 μs, and is assigned to the triplet state. DADS3 has a very similar lineshape as DADS2, as shown in the normalized spectra Fig. S27. However, the decay lifetime for the third component is ~8.2 μs, ~4 times longer than the second component. Since the ns-TA measurement is done in degassed toluene at room temperature, the lifetime of long-lived triplet in ms range for RTP emission in the PMMA matrix can be significantly reduced to μs regime because of molecular collisions. Nevertheless, the existence of two long-lived components is consistent with the EFA and MCR-ALS analysis. Furthermore, because the similarity of the DADS for component 2 and 3, it indicates that these two are triplet states with very similar energy levels.

The comparison of ns-TA measurements in degassed and aerated solutions is shown in Fig. S28. The long-lived ESA features at 400 nm and 600 nm in degassed solution is quenched by oxygen with the decay lifetime to a few hundred nanoseconds. Such a measurement indicates the triplet contribution within the wavelength region, consistent with the MCR-ALS and global analysis. As for 800 nm and 900 nm, similar quenching behavior is noted for the long-lived signal, while the short-lived decay is preserved in aerated solution, indicating the combined singlet and triplet contributions, which is consistent with DADS from the global analysis.

Specifically, at 600 nm, a mono-exponential fitting fails to fit the long-lived signals, while bi-exponential fitting can nicely describe the entire ESA behavior, with decay lifetimes close to that from the global analysis based on the parallel model. As a comparison, another global analysis based on the parallel model with only two components is done for the ns-TA dataset for 1.8-mDTAZ-PhtCz, with the DADS and kinetics fitting shown in Fig. S29. While such a model can nicely fit the early experimental decays in nanosecond regime, it fails to fit the long-lived signals with only one component in the microsecond regime. This justifies the validity of fitting the data with three principal components in order to appropriately describe the transient kinetics within the entire timescale. In conclusion, from the ns-TA measurements, we observed a singlet and two triplets contributing to the exciton dynamics in 1.8-mDTAZ-PhtCz, while the two triplet states have similar energy levels but different decay lifetimes.

### Theoretical molecular simulations

To further elucidate the excited states of 1.8-mDTAZ-PhtCz, we perform molecular simulations to calculate their excited state energies, including Time-Dependent Density Functional Theory (TDDFT) with three different functionals, hybrid functional B3LYP, omega-tuned range-separated hybrid functionals ωT-CAM-B3LYP and ωT-ωB97xd, and the Restricted Open-shell Kohn–Sham method (ROKS)^45^ with the LC-ωPBE08 functional. For all the calculations, the 6–311 g (d,p) basis set are applied. The calculated results for energy levels are summarized in Table S9. All the three TDDFT methods underestimate the optical energy gaps, while the ROKS method predicted the excitation energies of the lowest singlet (S_1_) and the two lowest triplet states (T_1_ and T_2_), consistent with the experimental ns-TA results. The ground state and excited state electron distributions are shown in Fig. S34. The excitation energy of the first excited state is 3.493 eV (354 nm), close to the maximum absorption peak at 345 nm, shown in Fig. 1a and Table 1.

**Fig. 3:**
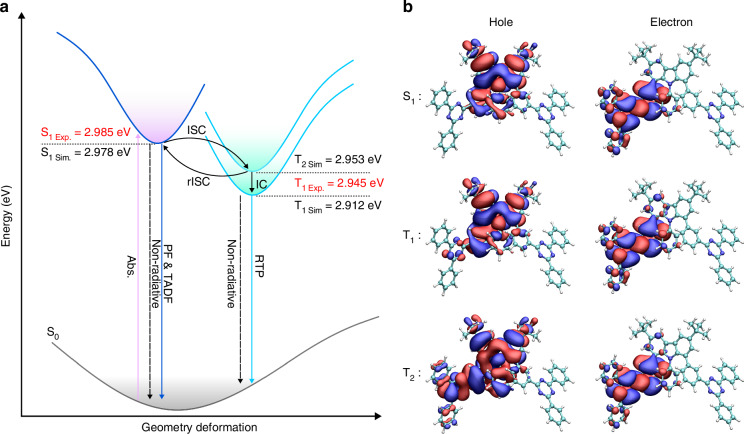
Molecular simulation. **a** Energy diagrams of 1.8-mDTAZ-PhtCz simulated based on ROKS. **b** The simulated hole and electron distributions for excited states

The calculated energy levels for the different excited states are shown in Fig. 3a. The calculated singlet state is 2.978 eV, based on the relaxed molecular configuration after photon excitation. The electron cloud distribution in Fig. 3b indicates that the singlet state has a pronounced CT feature, consistent with the experimental observations summarized in Table S1. The calculated energy level of S_1_ is close to the experimentally determined value of 2.985 eV, summarized in Table 1. The calculated energy level for T_1_ is 2.912 eV, which is also close to the experimental result of 2.945 eV. Moreover, another triplet state T_2_ with higher energy at 2.953 eV has been resolved, slightly lower than the simulated energy level of S_1_.

As shown in Fig. 3b, S_1_, T_1_, and T_2_ of 1.8-mDTAZ-PhtCz consist of a mixture of locally excited (LE) and CT states. The hole and electron wave functions of the S_1_ and T_1_ states are mainly located at the carbazole donor and phenyl-triazine acceptor, respectively. However, T_1_ exhibits more LE character compared to S_1_. In contrast, the hole state of the T_2_ is located on the carbazole donor and phenyl-triazine acceptor, while the electron state is mainly located on the phenyl-triazine acceptor, which means that the T_2_ state accounts for more LE contributions.

The energy level simulation indicates that efficient rISC can happen from T_2_ to S_1_. Also, since the energy splitting between S_1_ and T_2_ is smaller than S_1_-T_1_, higher ISC rate is anticipated from S_1_ to T_2_ than that from S_1_ to T_1_, according to the Fermi’s golden rule. Combined with the TA analysis in the previous section, we can then assign the two long-lived triplet states to T_2_ and T_1_, respectively.

### A four-level model

Based on the previous TA analysis and molecular simulations, we propose a four-level model with three excited states to describe the exciton kinetics in the model emitter. The specific model for the emitter 1.8-mDTAZ-PhtCz is shown in Fig. 3a. For simplicity, ISC and rISC are mainly happening between S_1_ and T_2_, which is similar to the three-level model for TADF emitters. Part of T_2_ will relax to T_1_ by internal conversion (IC), while the T_1_ state contributes to the phosphorescence. Since the energy difference between T_2_ and T_1_ is on the same order of the thermal energy at room temperature, thermal population from T_1_ to T_2_ is also possible, decreasing the density of T_1_ generated from IC. We here firstly assume that the generation rate from T_2_ to T_1_ with an effective IC rate *k*_IC_, indicating the final conversion rate from T_2_ to T_1_ after the IC from T_2_ to T_1_ and thermal excitation from T_1_ to T_2_. Furthermore, non-radiative losses are assumed from S_1_ with rate *k*_nr,S_ and T_1_ with non-radiative rate *k*_nr,T_. Since the radiative relaxation is competing with the non-radiative processes, the S_1_ intrinsic lifetime τ_S_ is kinetically defined as 1/(*k*_r,s_ + *k*_nr,s_), while the T_1_ lifetime τ_T_ as 1/(*k*_r,T_ + *k*_nr,T_). The bimolecular annihilation effects are assumed between S_1_-S_1,_ S_1_-T_2_ and T_1_-T_1_. In this kinetic model, one can describe the exciton kinetics with the following rate equations for S_1_ density $n$_S1_ (Eq. 1), T_2_ density $n$_T2_ (Eq. 2), and T_1_ density $n$_T1_ (Eq. 3), respectively:

1 $$\frac{d{n}_{{\rm{S}}1}}{{dt}}=-\frac{{n}_{{\rm{S}}1}}{{\tau }_{{\rm{s}}1}}-{k}_{{\rm{ISC}}}{n}_{{\rm{S}}1}+{k}_{{\rm{rISC}}}{n}_{{\rm{T}}2}-{k}_{{\rm{SSA}}}{n}_{{\rm{S}}1}^{2}-{k}_{{\rm{STA}}}{{n}_{{\rm{S}}1}n}_{{\rm{T}}2}$$

2 $$\frac{d{n}_{{\rm{T}}2}}{{dt}}={k}_{{\rm{ISC}}}{n}_{{\rm{S}}1}-{k}_{{\rm{rISC}}}{n}_{{\rm{T}}2\,}-{k}_{{\rm{IC}}}{n}_{{\rm{T}}2}-{k}_{{\rm{STA}}}{{n}_{{\rm{S}}1}n}_{{\rm{T}}2}$$

3 $$\frac{d{n}_{{\rm{T}}1}}{{dt}}=-\frac{{n}_{{\rm{T}}1}}{{\tau }_{{\rm{T}}1}}+{k}_{{\rm{IC}}}{n}_{{\rm{T}}2}-{k}_{{\rm{TTA}}}{n}_{{\rm{T}}1}^{2}$$

The transient PL decay of 1.8-mDTAZ-PhtCz has been studied by TCSPC measurements over the time range from ns to second region. To capture the full picture of the PL decay in different time domains, the repetition rate of the excitation laser is tuned to sequentially measure the PF in the ns region, delayed fluorescence in the μs region, and long-lived RTP emission in the ms to second region. Since the long-lived RTP spans a long-time window, to get a reasonable signal-to-noise ratio, the excitation laser was working in a burst mode. A number of pulses in 1 MHz repetition rate was used to accumulate sufficient emissive T_1_, then the long-lived emission was detected with the laser off. In such a case, it is possible that part of the accumulated long-lived triplets got quenched because of annihilation. The detailed laser settings for the TCSPC measurements can be found in the experimental section.

**Fig. 4:**
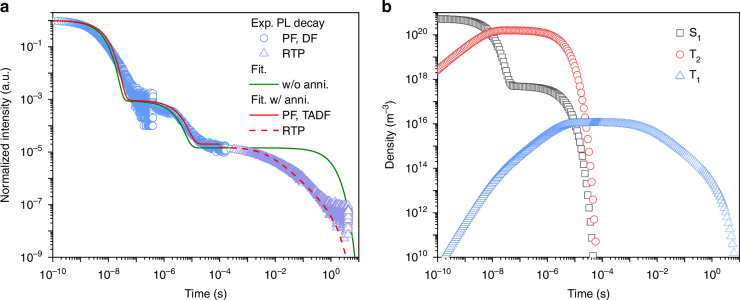
Exciton kinetics mechanism. **a** Normalized experimental transient PL decay with PF, DF and RTP at room temperature, fitted with the proposed four-level model, with (red lines) or without (green line) annihilation effects between singlet-singlet (S_1_-S_1_), singlet-triplet (S_1_-T_2_) and triplet-triplet (T_1_-T_1_). **b** Modelled exciton densities with the consideration of annihilation effects. For both plots, the accumulated density of T_1_ after 1 × 10^−^^4^ s is rescaled to match the density after a single pulse excitation

The entire transient PL decay is shown in Fig. 4a. After the PF decay in ns region, a pronounced TADF feature with lifetime in μs is observed. Furthermore, a very long-lived RTP emission of hundreds of ms is detected. The exciton density evolution can be obtained by fitting the experimental decay numerically based on the four-level model with Eqs. 1–3. The fitting parameters are summarized in Table 2. For 1.8-mDTAZ-PhtCz, the intrinsic lifetime of S_1_ is ~8 ns, while the lifetime of T_1_ is ~0.75 s. The lifetime of the T_2_ state can be estimated by 1/*k*_rISC_, which is about 1.8 μs. Since the IC rate is the effective transition rate from T_2_ to T_1_ between the competition between the internal conversion and thermal excitation, the low effective IC rate indicates that T_1_ can be thermally populated with a similar rate of internal conversion between T_2_ and T_1_.

**Table 2:** Kinetic modelling parameters for transient PL decay with bimolecular annihilation effects

τ_S1_ (s)	*k*_ISC_ (s^−1^)	*k*_rISC_ (s^−1^)	*k*_IC_ (s^−1^)	τ_T1_ (s)	*k*_SSA_ (m^−3^s^−1^)	*K*_STA_ (m^−3^s^−1^)	*k*_TTA_ (m^−3^s^−1^)
8.0 × 10^−9^	5.5 × 10^7^	5.5 × 10^5^	25	0.75	8.0 × 10^−18^	8.0 × 10^−18^	4.0 × 10^−18^

The exciton density evolution for each excited state is plotted in Fig. 4b. Because the singlet lifetime is only ~8 ns, the decay of PF till hundreds of ns results from the direct relaxation of S_1_ to the ground state. The delayed fluorescence within 10^−7^ to 10^−5^ s mainly comes from rISC from the T_2_ to S_1_ state. The slow radiation of T_1_ contributes to the long-lived RTP emission from 10^−4^ to 10 s. Because of annihilation effects, the decay deviates from the perfect monoexponential behavior within each part, as shown in Fig. 4a.

Multi-level models with a higher-lying triplet state have already been tested for TADF emitters, however, without considering triplet annihilation effects^44,45^. The reason for this omission is that typically for TADF emitters the T_1_ lifetime is generally in the μs region due to the efficient rISC process at room temperature^46^. In contrast, in our model emitter the T_1_ lifetime is in the range of hundreds of ms, resulting in much higher triplet densities after accumulation and therefore enhanced triplet annihilation processes. Because of the occurrence of triplet annihilation effects, the analytical expressions derived for the conventional multi-level model cannot be used^44^, and only numerical modelling with bimolecular annihilation can be adopted to describe the transient photoluminescence of our model emitter, as shown in Fig. 4.

Another four-level model has been tested with the consideration of rISC from T_1_ to S_1_, and the reverse internal conversion (rIC) from T_1_ to T_2_, as shown in Fig. S24. To estimate the kinetic rates of rISC from T_1_ to S_1_, and rIC from T_1_ to T_2_, additional assumptions that the rISC rates from T_1_ and T_2_ to S_1_, and the IC and rIC rate following the Arrhenius equation are needed. As shown in Fig. S25 in supplementary note S2, from the numerical fitting of the experimental PL decay, it is estimated that the energy difference between T_2_ and T_1_ around 250–300 meV under the additional assumptions. Furthermore, the contribution of the rISC from T_1_ to S_1_ is negligible compared to the rISC from T_2_ to S_1_. The rIC rate is also orders of magnitude smaller than the IC rate. Such a check further demonstrates that the simplified four-level model is enough to describe the exciton dynamics without considering rISC from T_1_ to S_1_, or the reverse internal conversion (rIC) from T_1_ to T_2_. However, the obtained T_2_-T_1_ splitting of 250-300 meV depends on the validity of *k*_rIC_/*k*_IC_ = exp(-Δ*E*/*k*_B_T). Because the TADF and RTP mechanism can give complementary effects on the delayed PL as the temperature decreases (Fig.1c), it is difficult to experimentally confirm this relation. It is noted that for pure TADF emitters such as 4CzIPN, there is a large deviation between the ratio of *k*_rISC_/*k*_ISC_ and exp(-Δ*E*_ST_/*k*_B_T), as the kinetic rates and activation energy can be experimentally measured independently^47^. Due to the strong assumption of rIC and IC rates *k*_rIC_/*k*_IC_ = exp(-Δ*E*/*k*_B_T), the energy difference determined from kinetic fitting can be deviating from the real case, different from that estimated from the molecular simulation or spectral analysis.

The detailed mechanism of such a slow IC and rIC rate between T_2_ and T_1_ in the model emitter is beyond the scope of current work and is an on-going project. A possible mechanism is the mixing properties with charge-transfer and local-excited states for S_1_, T_2_ and T_1_. As shown in Fig. 3b, the hole and particle NTOs of the excited states show a signature of state mixing between spatial separation and orbital overlap, indicating the coexistence of CT and LE components. Moreover, the phosphorescence spectra of the model emitter also deviate from the PL spectra of the acceptor or donor group, giving the hint that the T_1_ state is also a mixing of CT and LE properties^14,48^. Due to different properties of T_2_ and T_1_ with varied degree of state mixing, it is possible that the IC and rIC rate is low. The decoupling of T_1_ and T_2_ in the model emitter is consistent with the previous reports in purely organic hot-exciton materials for efficient OLEDs without TADF or RTP, in which the rISC process happens from higher-lying triplets (i.e., T_2_) to S_1_, outcompeting the internal conversion to T_1_ with a much lower energy than S_1_^49^.

In summary, the simultaneous occurrence of PF in nanoseconds, TADF in microseconds and RTP in seconds is experimentally observed from 1,8-mDTAz-PhTCz when doped in a polymeric host. The RTP emission can further elongate to dozens of seconds in crystals. However, RTP is fully quenched in degassed toluene, which is consistent with the universal observation that long-lived triplet emission is quenched in solvents at room temperature due to molecular collision. Meanwhile, from transient absorption spectroscopy, three excited states including one singlet state with lifetime in nanoseconds, and two triplet states with lifetime in microseconds are observed in degassed solvents. On the other hand, the three-level model fails to describe the TA results, or the presence of PF, TADF and RTP with lifetimes with differences in several orders of magnitude. From the exciton kinetic modelling, systems with a three-level model including a ground, singlet and triplet state can only lead to transient PL decays with two transient lifetimes. This is due to the competing nature of TADF process and RTP emission in the three-level model. With a single triplet level contributing TADF and RTP, the faster process between rISC and triplet decay will take over the slower one. The four-level model with two triplet states and bimolecular annihilation can explain the TA results and PL decay, consistent with the molecular simulation results. The materials are purified by column chromatogram and sublimation before photophysical investigations. The HPLC and NMR results demonstrate that the purity should not be a problem^50^. Due to molecular conformational disorder, there is another possibility that a proportional of emitters give only PF and RTP emission, while some of the other molecules with a relatively smaller Δ*E*_ST_ contributing to PF and TADF^51,52^. From the molecular structure shown in Fig. 1d, this is less likely because there is limited freedom to tune the molecular conformation between the donor and acceptor groups (i.e., θ_1_ and θ_3_ in Fig. 1d), because of the steric hindrance of the benzene ring linking to the carbazole donor. However, it is still difficult to directly exclude the potential contribution of conformational disorder at this moment. Single-molecule spectroscopic studies may facilitate to exclude the potential contribution of conformational disorders, but it is beyond the scope of current study^53^.

### Multi-color emissive system by Förster resonance energy transfer

**Fig. 5:**
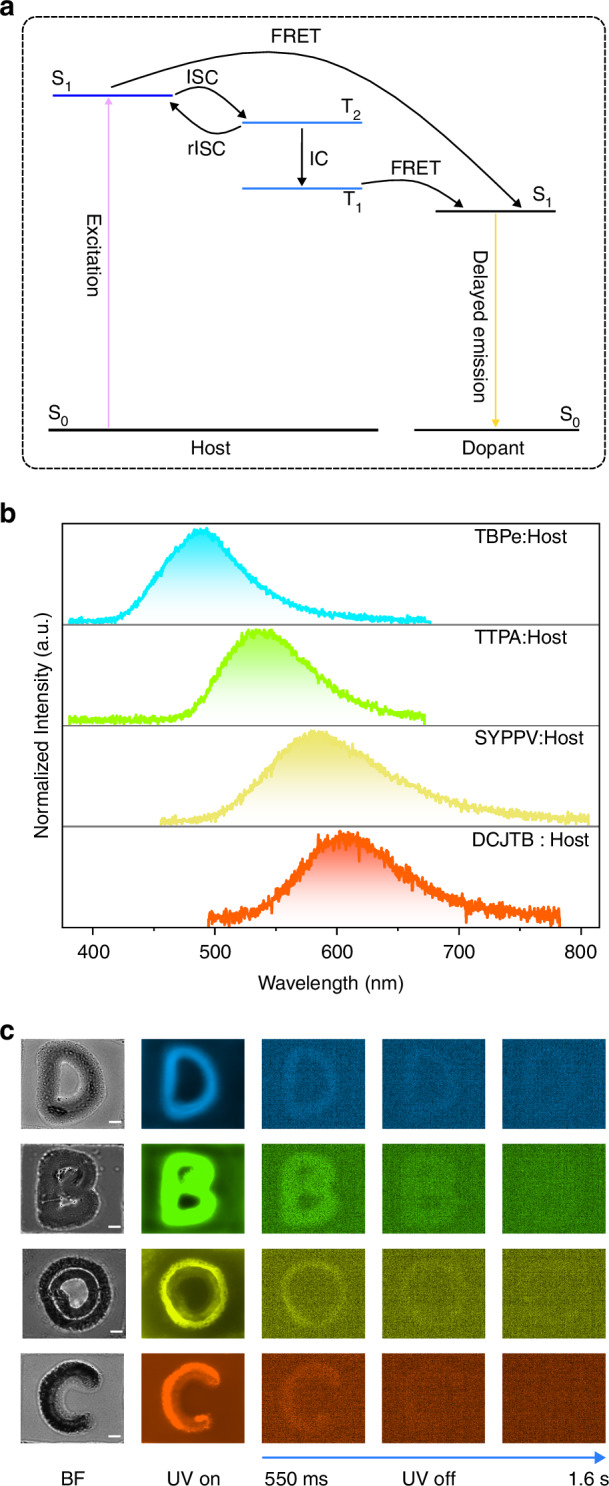
Multi-color FRET systems. **a** A possible diagram of energy transfer to obtain multi-color PF-TADF-RTP systems. **b** Delayed emission at 200 μs with different dopants. The doping concentrations are 2 wt% for TBPe, 8 wt% for TTPA, 2 wt% for SYPPV, 8 wt% for DCJTB. **c** Time-resolved microscopy images with tunable delayed emission color by doping different conventional fluorescent emitters. The letters D, B, O, and C are doped with TBPe, TTPA, SYPPV, and DCJTB (molecular structure in Fig. S35), respectively. The scale bar is 5 μm. BF bright field

Based on the fascinating photophysical properties of the model emitter 1.8-mDTAZ-PhtCz, we here demonstrate that by utilizing the energy of these multiple excited states, it is possible to obtain emissive systems with multi-color emission within a window from ns to ms region. The diagram of singlet energy transfer in such a system with multiple excited states is shown in Fig. 5a. We select some conventional fluorescent emitters as an example, including the blue emitter 2,5,8,11-tetra-tert-butylperylene (TBPe), green emitter 9,10-bis[N,N-di-(*p*-tolyl)-amino]anthracene (TTPA), super yellow poly(*p*-phenylene-vinylene) polymer (SYPPV) and red emitter 4-(dicyanomethylene)-2-tert-butyl-6-(1,1,7,7-tetramethyljulolidin-4-yl-vinyl)-4H-pyran (DCJTB). Their chemical structures are shown in Fig. S35. It is possible to further extend into the deep red or even NIR region by mixing emitters with lower energy gaps. In the framework of FRET, the energy transfer rate increases with the distance decrease between the donor and acceptor, as well as their spectral overlap^54^.

As plotted in Fig. S36, there is significant spectral overlap between the absorption of the dopant emitters and the delayed PL spectra of 1.8-mDTAZ-PhtCz. When increasing the dopant concentration, the emission originating from the donor is gradually reduced, as shown in Fig. S37. The distance between the acceptor and donor emitter decreases by increasing the dopant concentration, leading to a higher FRET rate. Complete energy transfer is achieved when the emission from 1.8-mDTAZ-PhtCz is fully quenched. Because of the up-conversion from T_1_ to T_2_ and then S_1_ in 1.8-mDTAZ-PhtCz, long-lived PL emission can be observed with conventional fluorescent dopants^54^. The delayed emission spectra with different dopants at 200 μs are shown in Fig. 5b. As shown in Fig. S38, by mixing conventional fluorescent emitters, we can obtain an emissive system within the entire visible wavelength range with delayed emission covering ns to second region.

The multi-color emissive system has quite some potential applications, such as bio-imaging or data encryption. We here show a demo for time-resolved microscopy as a proof-of-concept. The sample preparation with pre-defined microscopic patterns is schematically illustrated in Fig. S39. The letters *D*, *B*, *O*, and *C* in a pre-engraved coverslip were casted with a mixture of 1.8-mDTAZ-PhtCz and various dopants in a PMMA matrix. A gradual PL decay with different emission colors can be observed after the UV excitation. As presented in Fig. 5c, delayed emission till ~1.6 s is still observable. Due to the simultaneous emission of PF, TADF and RTP from the same luminescent material system, it is possible to select a delayed time window to capture the image from the sample. Furthermore, as shown in Fig. S40, when using luminescent systems with different emissive colors in one pattern, by selecting different detection wavelength and time window, only the area with a specific color within the exposure time windows can be detected. Therefore, such a color tunable luminescent system from blue, green, yellow to red, together with the simultaneous PF, TADF and RTP emission can potentially be applied for bio-imaging and data encryption.

### Application for efficient RTP materials development

**Fig. 6:**
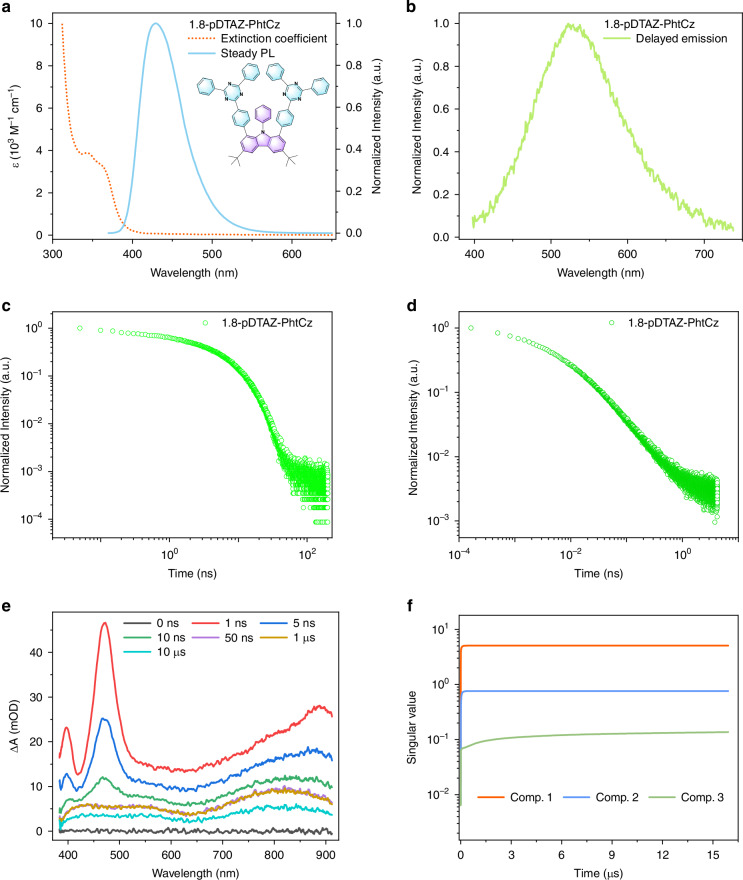
Photophysical properties of 1.8-pDTAZ-PhtCz. **a** Steady state UV-Vis absorption and PL emission spectra in degassed toluene at room temperature. Inset: chemical structure. **b** Phosphorescence spectrum. Recorded with a 200 μs delay after laser excitation in nitrogen atmosphere at room temperature with a doping concentration of 2 wt% in PMMA. **c** PF decay of 2 wt% 1.8-pDTAZ-PhtCz doped in PMMA. Measured at room temperature with a detection wavelength of 441 nm. **d** Long-lived phosphorescence decay of 2 wt% 1.8-pDTAZ-PhtCz in PMMA at room temperature monitored at 523 nm. **e** Selected ns-TA spectra for 1.8-pDTAZ-PhtCz dissolved in degassed toluene. **f** The EFA for ns-TA spectra in degassed toluene. Here, only the first three major singular values are presented for simplification, since they are orders larger than the residual

The presence of the additional higher-lying triplet state with energy lower than the singlet state makes it possible to develop efficient organic luminescent materials. A derivative 1.8-pDTAZ-PhtCz with its chemical structure shown in Fig. 6a, has been obtained based on the model emitter, with a slightly tuned energy difference between these excited states (S_1_, T_2_ and T_1_) by increasing the conjugation length between the donor and acceptor. Molecular packing of its single crystal is shown in Fig. S19 and Table S8. Ultralong afterglow emission has also been observed from crystals of 1.8-pDTAZ-PhtCz, as shown in Fig. S20.

The photophysical properties of the derivative have been systematically characterized by ns-TA and transient PL spectroscopy. As shown in Fig. 6a and Fig. 6b, the absorption onset is 400 nm, while the fluorescence and delayed emission spectral peaks are at 430 nm and 523 nm, respectively. The energy levels for singlet S_1_ (2.881 eV) and triplet T_1_ (2.578 eV) are shown in Fig. S17 and Table 1, respectively, determined by lineshape analysis of its fluorescence and phosphorescence spectra. Because of a reduction of the triplet energy levels, a larger Δ*E*_ST_ of 0.3 eV between S_1_-T_1_ is noted for 1.8-pDTAZ-PhtCz. As a result, the TADF effect is suppressed, with now only pronounced RTP emission with a high quantum yield exceeding 30%. The detailed photophysical properties of 1.8-pDTAZ-PhtCz are summarized in Table 1, along with those of the model emitter. The fluorescence and phosphorescence transient PL decays are shown in Figs. 6c and 6d. As shown in Fig. S14, the fluorescence lifetime of S_1_ is 5.2 ns, while the phosphorescence lifetime of T_1_ is ~118.7 ms. The lifetime observed in the PS matrix is similar with that measured in the PMMA matrix (Fig. S15). Based on the quantum yields and emission lifetimes for fluorescence and phosphorescence, the ISC rate from singlet to triplet states can be estimated based on a method reported previously, by assuming the non-radiative loss only from singlets or triplets^11^. As shown in Table 2 and Table S5, the derivative compound has a comparable ISC rate with the model emitter 1.8-mDTAZ-PhtCz.

Pronounced ESA are observed in the ns-TA spectra of 1.8-pDTAZ-PhtCz, as shown in Fig. 6e. The 2D ns-TA spectra of 1.8-pDTAZ-PhtCz in degassed and aerated toluene are shown in Fig. S33. ESA with fast decay is observed within the ns timescale, which can be assigned to singlets. Afterwards, a long-lived ESA within the time window of 20–50 μs persists. The EFA to determine the principal components of the ns-TA spectral data, shown in Fig. 6f, indicates that there are three principal components, which is the same for the model emitter 1.8-mDTAZ-PhtCz.

Furthermore, the ns-TA spectra for 1.8-pDTAZ-PhtCz in degassed toluene are fitted by the MCR-ALS method, shown in Fig. S30. It is noted that the first and second component are correlated. The fastest component has a decay lifetime in the ns region, correlated with the concentration increase of the second component with a slightly longer decay lifetime. The second component decays within the μs region, while the increase of the concentration of the slowest component is related to the concentration decrease of the second component. The MCR-ALS fitting and forward EFA analysis indicate that a singlet and two triplet states are involved in the dynamics of this derivative. Thus, similar exciton dynamics have been resolved in the ns-TA spectra as compared to the model emitter 1.8-mDTAZ-PhtCz.

Based on a parallel scheme, it is difficult to distinguish the slower components by the global analysis. This is indicated by the large uncertainty of decay lifetime and the similar or mirror-like DADS for the slower components, as shown in Fig. S30. The possible reason could be the small energy difference between T_2_ and T_1_, or similar lifetime in degassed solvent for these triplet states. The two triplet states can be resolved in a better manner whenever measured in aerated solvents, due to a different quenching behavior of the two triplet states with oxygen molecules. The detailed analysis for ns-TA data with oxygen quenching has been summarized in Fig. S31 and Fig. S32.

Nevertheless, the ns-TA spectroscopy measurements indicate the existence of an additional upper triplet state for 1.8-pDTAZ-PhtCz. However, with relatively larger energy difference between singlet and triplet states (Table 1), only RTP emission can be obtained without TADF contribution. The quantum yield of the RTP emission for 1.8-pDTAZ-PhtCz is more than 30%.

It is noted that the RTP quantum yield in purely organic PF/RTP emitters is normally less than 10% because of relatively low ISC rates and/or intrinsically slow triplet decay. The newly developed derivative of the model emitter 1.8-pDTAZ-PhtCz is 2–3 times higher than conventional purely organic emitters, as compared to reported purely organic RTP emitters with carbazole as the donor summarized in Table S10^55–59^. Compared to dual emission PF/RTP materials with two excited states (S_1_ and T_1_), there should be at least three excited stated involved for materials with simultaneous PF/TADF/RTP emission. The key mechanistic difference is presented in Fig. S23. Therefore, the current work will pave a new avenue for developing purely organic RTP materials by tuning the relative energy difference between S_1_ and T_2_/T_1_ states and their orbital properties.

Vice versa, the higher-lying triplet state can be potentially meaningful for TADF emitter design as well. Because the T_2_ level is higher than T_1_ resulting in smaller Δ*E*_ST_ between T_2_ and S_1_, as compared to T_1_ and S_1_, the rISC rate from T_2_ to S_1_ should be faster than that from T_1_ to S_1_. Thus, it is possible to enhance the rISC rate for TADF emitters with shorter triplet lifetime and lower triplet density in the device, which is of vital importance for operational stable organic light-emitting diodes^32^. The numerical modelling on exciton kinetics and experimental verification by transient photoluminescence and TA spectroscopic methods in the current work can shine light on future TADF designs and photophysical studies.

## Discussion

In summary, we demonstrated that multiple excited states are involved to obtain PF, TADF, and RTP from a single emitter. For emitters with only T_1_ and S_1_, it is not possible to simultaneously obtain efficient TADF and RTP with orders of decay lifetime difference. We experimentally and theoretically confirmed that another upper triplet state T_2_ is involved in the emissive process. We demonstrated that it is possible to obtain multi-color and long-lived emission by doping with different fluorescent emitters via Förster resonance energy transfer. The doped multi-color system still exhibits transient emission spanning 8–9 orders of time scale. Such an emissive system makes it possible to select different time windows and wavelength regions for microscopic applications. By tuning the relative energy difference between these exciton levels, we obtained highly luminescent materials with only prompt fluorescence and RTP emission, while the RTP quantum yield can reach more than 30%. We anticipate that this work can enhance the fundamental understanding on the photon generation from the purely organic emitters, and therefore the manipulation of the emission behavior either under optical or electrical excitation.

## Materials and Methods

### Materials

The synthesis details, NMR, and HRMS results of 1.8-mDTAZ-PhtCz and 1.8-pDTAZ-PhtCz can be found in Supplementary Information. TBPe and SYPPV were obtained from Sigma-Aldrich. TTPA and DCJTB from BLD Pharmatech. These doped emitters are used as received. The host emitter was purified by sublimation before testing.

### Doped film fabrication

PMMA/PS was dissolved in toluene at a concentration of 60 mg/mL, and a corresponding percentage of organic material was added to the solution of PMMA/PS to obtain different doping concentrations. These films were then prepared by blade coating onto cleaned and oxygen plasma-treated glass substrates. For quantum yield measurements, films were prepared by spin-coating on quartz substrates.

### Photophysical properties measurement

Steady state UV-Vis absorption spectra were measured on a Perkin-Elmer Lambda 900 spectrophotometer at room temperature. Steady-state PL spectra were recorded with a HORIBA Jobin-Yvon Fluorolog 3-22 Tau-3 using a photomultiplier tube (PMT) as detector, using 356 nm excitation. Time-resolved emission spectra and temperature-dependent PL decay in Fig. 1 were measured by using a 4Picos gated-iCCD camera (Stanford Computer Optics) with a Ti-sapphire laser (Coherent, Astrella, 5 mJ, 800 nm, 1 kHz). The PL decay at room temperature in Fig. 4 was obtained by means of TCSPC setup (Picoquant), with a repetition-rate tunable ps-laser at 375 nm, Peltier cooled PMT with the instrument response < 180 ps, and a TimeHarp card with 32,768 time bins and 32 bit depth. The laser was modulated in pulse mode to measure the prompt (1 MHz) and delayed fluorescence decay (10 KHz), while the laser was working in burst mode to measure the long-lived RTP decay. During the burst mode, to accumulate a sufficient population of long-lived triplet states, excited with 1 MHz laser for 100 ms, followed with the longest measurement time window of ~4.3 s defined by the setup (Fig. S41). The ns-TA spectra were measured by using an EOS pump-probe setup (Ultrafast Systems) paired with an amplified 1030 nm fs laser (Pharos, Light Conversion, 200 fs, 200 µJ) with an effective laser repetition rate of 1 kHz (set via an internal pulse picker). The probe white light was generated via a sub-nanosecond pulsed light source and a photonic crystal fiber, yielding broadband probe light across the UV-Vis-NIR regions. The pump pulse at 360 nm was generated with an optical parametric amplifier (Orpheus-F, Light Conversion). The pump energy was 200 μW. Samples were dissolved in degassed toluene as 4 mg/mL and measured in 2 mm path length cuvettes.

### Computational details

The geometry optimization of the molecule was performed in Qchem 6.5 using DFT at range-separated hybrid functionals LC-ωPBE08/6-311 g(d,p) level of theory^60^ in combination with DFT-D3(BJ) dispersion correction from Grimme et al.^61^ and a polarizable continuum model (PCM), where the toluene as solvent is used. The lowest lying singlet (S_1_) and triplet (T_1_) excited states are calculated with the spin-restricted open-shell Kohn–Sham method (ROKS)^60,61^ in combination with DFT-D3(BJ) and PCM (toluene as solvent). For the second lowest lying triplet (T_2_) excited state, the same level of theory is applied, however, instead of using the Geometric Direct Minimization (GDM)^62^ algorithm, the Square Gradient Minimization (SGM)^63^ is used for optimizing the excited state orbitals. Note that, the emission spectra, i.e. S_1_, T_1_, and T_2_ energies, are calculated at the optimized S_1_ geometry. Hole–electron analysis was displayed using the visual molecular dynamics (VMD) software package^63^. For comparison, the results of B3LYP/6-311 g (d,p), the ω tuned CAM-B3LYP/6-311 g (d,p) and the ω tuned ωB97XD/6-311 g (d,p) are also listed in Table S8.

### Time-resolved microscopy

The doped films were prepared with materials mixed in PMMA as described above. All films and crystal for microscopy were prepared with oxygen plasma-treated gridded glass coverslip (ibidi Grid-50) performed in a nitrogen glove box and finally been sealed. The time-resolved phosphorescence images were performed using the Thunder system (Leica) equipped with a sCMOS camera (Leica DFC9000). A 390 nm LED was selected for illumination, which equipped with a quadband filter cube (the excitation filter 375–407 nm, 462–496 nm, 542–566 nm, 622–654 nm; the dichroic beam splitter 415, 500, 572, 660 nm and the emission filter 420-450 nm, 506-532 nm, 578–610 nm, 666–724 nm). Here, we chose the same LED and corresponding quadband filtercube for the fluorescence and phosphorescence channels, except that the LED for phosphorescence channel was turned off. For the 1.8-mDTAZ-PhtCz and 1.8-pDTAZ-PhtCz crystal, the phosphorescence images were acquired with camera exposure time of 6 s for each frame after illuminated by 390 nm LED with 100% power. After the crystal was continuously excited by the LED for 200 ms, after another 19 ms, the phosphorescence images were began to be recorded with an 11 ms interval between each phosphorescence image. HC PL FLUOTAR 10X/0.32 DRY objective was selected, pixel size is 1.291 μm. For the fluorescence images of doped films, power of LED was set to 1%, 1%, 100%, and 16% for TBPe, TTPA, SYPPV, and DCJTB, separately. The phosphorescence images were acquired with camera exposure time of 100 ms for each frame after illuminated with 390 nm LED. After the film was continuously excited by the LED for 10 ms, after another 550 ms, the phosphorescence images were beginning to be recorded with a 400 ms interval between each phosphorescence image. HC PL APO CS2 63X/1.40 OIL objective was selected, pixel size is 0.205 μm. It should be noted that the time switched the fluorescence channel to the phosphorescence channel and the time switched the phosphorescence channel to the next phosphorescence channel is different, which we specifically marked in the picture. For each sample, the brightness/intensity range of all phosphorescence images was set to the same as that of the first phosphorescence image.

## Supplementary information

Simultaneous delayed fluorescence and phosphorescence in organic luminescent material employing multiple excited states.

## Data Availability

The data that support the findings of this study are available from the corresponding author upon reasonable request.
