# Supplementary material for: Simultaneous delayed fluorescence and phosphorescence in organic luminescent material employing multiple excited states
Source: Light Sci Appl. 2026 Jan 1;15:4. doi: 10.1038/s41377-025-02063-x (PMC12756230; doi:10.1038/s41377-025-02063-x)
Supplement: Supplementary file 1 — Simultaneous delayed fluorescence and phosphorescence in organic luminescent material employing multiple excited states. [file 41377_2025_2063_MOESM1_ESM.docx]

Supporting Information

**Simultaneous delayed fluorescence and phosphorescence in organic luminescent material employing multiple excited states**

Dehai Dou,^‡^ Wenlan Liu,^‡^ Xin Zhou, Qiqi Yang, Xiao Tan, Naz Ugur, Chongyao Li, Charusheela Ramanan, Xiaomin Liu, Gert-Jan A. H. Wetzelaer, Denis Andrienko, Martin Baumgarten*, Paul W. M. Blom*, and Yungui Li*

**Table of contents**

[1. Synthesis and characterization 2](#_Toc188614871)

[2. X-ray crystal structure analysis and crystal imaging 13](#_Toc188614872)

[3. Supplementary note S1: Three-level model 17](#_Toc188614873)

[4. Supplementary note S2: Four-level model 21](#_Toc188614874)

[5. Transient absorption spectroscopy 24](#_Toc188614875)

[6. DFT calculations 29](#_Toc188614876)

[7. Multi-color FRET systems 30](#_Toc188614877)

# 1. Synthesis and characterization

Scheme S1 Synthetic routes for the 1.8-mDTAZ-PhtCz and 1.8-pDTAZ-PhtCz.

3,6-Di-tert-butyl-9-phenyl-9H-carbazole (PhtCz).

A mixture of iodobenzene (0.61 g, 30 mmol), 3,6-di-tert-butyl carbazole (0.28 g, 10 mmol), copper(I) iodide (0.19 g, 1 mmol), N, N-diisopropylethylamine (0.05 g, 0.4 mmol) and potassium hydroxide (1.1 g, 20 mmol) were added in N, N-dimethylformamide (100 mL). The reaction was carried out under nitrogen atmosphere at 110 °C for 24 hours. After cooling down to room temperature, the mixture was poured into 100 mL water, and extracted with dichloromethane (DCM) three times. The organic layer was then dried over anhydrous Na_2_SO_4_. The crude product was purified by column chromatography to give a white solid compound (yield: 84%). ^1^H NMR (700 MHz, CDCl_3_) δ 8.18 (d, J = 1.9 Hz, 2H), 7.62 – 7.58 (m, 4H), 7.48 (dd, J = 8.7, 1.9 Hz, 2H), 7.45 (td, J = 6.8, 2.2 Hz, 1H), 7.38 (d, J = 8.6 Hz, 2H), 1.50 (d, J = 1.1 Hz, 18H). ^13^C NMR (176 MHz, CDCl_3_) δ 142.93, 139.42, 138.36, 129.88, 127.10, 126.92, 123.71, 123.48, 116.35, 109.33, 34.88, 32.18. High-resolution mass spectrometry (HRMS) (APCI) m/z: calcd for C_26_H_29_N: 355.2300; found: 355.2299.

1,8-Dibromo-3,6-di-tert-butyl-9-phenyl-9H-carbazole (1.8-DBr-PhtCz).

3,6-Di-tert-butyl-9-phenyl-9H-carbazole (1.5 g, 0.42 mmol), NBS (1.5 g, 8.6 mmol), DMF (60 mL) and CHCl_3_ (40 mL) were added into a 250 mL flask. The mixture was stirred at 80 °C in the dark for 12 h. After cooling to room temperature, the mixture was poured into 100 mL water, and extracted with DCM for three times. The organic layer was washed with brine and then dried over anhydrous Na_2_SO_4_. The solution was filtered and concentrated by a rotary evaporator. The crude product was purified by column chromatography to give a white solid product. (yield: 75%).^1^H NMR (700 MHz, CDCl_3_) δ 8.09 (dd, J = 1.9, 0.9 Hz, 2H), 7.64 (dd, J = 1.8, 0.9 Hz, 2H), 7.58 – 7.53 (m, 1H), 7.50 – 7.45 (m, 4H), 1.47 (d, J = 1.2 Hz, 18H) . ^13^C NMR (176 MHz, CDCl_3_) δ 144.78, 138.41, 137.15, 132.77, 130.28, 129.34, 128.02, 125.58, 115.51, 103.92, 34.79, 31.94. High-resolution mass spectrometry (HRMS) (APCI) m/z: calcd for C_26_H_27_Br_2_N: 511.0510; found: 511.0508.

3,6-Di-tert-butyl-1,8-bis(3-(4,6-diphenyl-1,3,5-triazin-2-yl)phenyl)-9-phenyl-9H-carbazole (1.8-mDTAZ-PhtCz).

A mixture of 1.8-DBr-PhtCz (0.15 g, 0.29 mmol ) and 2,4-diphenyl-6-(3-(4,4,5,5-tetramethyl-1,3,2-dioxaborolan-2-yl)phenyl)-1,3,5-triazine (0.26 g, 0.61 mmol), Pd_2_(dba)_3_ (0.027 g, 0.029 mmol), dicyclohexyl(2',6'-dimethoxy-[1,1'-biphenyl]-2-yl)phosphane (SPhos) (0.049 g, 0.12 mmol) and K_2_CO_3_ (0.16 g, 1.16 mmol) were dissolved in a mixture of THF and water in an argon atmosphere. The mixture was heated to 80 °C and stirred for 24 h. After cooling to room temperature, the mixture was washed with brine and then extracted with DCM. Afterwards, the mixture was dried over anhydrous Na_2_SO_4_. The solution was filtered and then concentrated by a rotary evaporator. The product was purified by column chromatography. (yield: 45%). ^1^H NMR (700 MHz, CD_2_Cl_2_) δ 8.68 – 8.66 (m, 8H), 8.38 (d, *J* = 2.0 Hz, 2H), 8.32 (d, *J* = 7.8 Hz, 2H), 8.06 (s, 1H), 7.53 (d, *J* = 7.3 Hz, 13H), 7.43 (d, J = 2.0 Hz, 3H), 7.17 (s, 3H), 6.68 (s, 2H), 6.12 (s, 3H), 1.56 (s, 18H). ^13^C NMR (176 MHz, CD_2_Cl_2_) δ 171.64, 143.00, 140.48, 139.66, 137.81, 136.64, 133.80, 132.82, 129.16, 128.99, 127.98, 127.77, 127.67, 126.64, 126.51, 124.75, 115.96, 35.06, 32.21. High-resolution mass spectrometry (HRMS) (APCI) m/z: calcd for C_68_H_55_N_7_: 969.4519; found: 969.4504.

3,6-Di-tert-butyl-1,8-bis(4-(4,6-diphenyl-1,3,5-triazin-2-yl)phenyl)-9-phenyl-9H-carbazole (1.8-pDTAZ-PhtCz).

A mixture of 1.8-DBr-PhtCz (0.15 g, 0.29 mmol ) and 2,4-diphenyl-6-(4-(4,4,5,5-tetramethyl-1,3,2-dioxaborolan-2-yl)phenyl)-1,3,5-triazine (0.26 g, 0.61 mmol), Pd_2_(dba)_3_ (0.027 g, 0.029 mmol), dicyclohexyl(2',6'-dimethoxy-[1,1'-biphenyl]-2-yl)phosphane (SPhos) (0.049 g, 0.12 mmol) and K_2_CO_3_ (0.16 g, 1.16 mmol) were dissolved in a mixture of THF and water in an argon atmosphere. The mixture was heated to 80 °C and stirred for 24 h. After cooling to room temperature, the mixture was washed with brine and then extracted with DCM. Afterwards, the mixture was dried over anhydrous Na_2_SO_4_. The solution was filtered and then concentrated by a rotary evaporator. The product was purified by column chromatography. (yield: 48%). ^1^H NMR (400 MHz, CD_2_Cl_2_) δ 8.76 – 8.69 (m, 8H), 8.36 – 8.27 (m, 6H), 7.65 – 7.55 (m, 11H), 7.54 (t, J = 1.8 Hz, 1H), 7.40 (d, J = 2.0 Hz, 2H), 7.17 – 7.09 (m, 4H), 6.74 – 6.66 (m, 2H), 6.64 – 6.51 (m, 3H), 1.54 (s, 18H). ^13^C NMR (101 MHz, CD_2_Cl_2_) δ 171.90, 171.86, 144.97, 143.09, 139.89, 137.95, 136.72, 133.94, 132.84, 130.14, 129.80, 129.19, 129.01, 127.92, 127.89, 127.57, 126.87, 126.37, 124.90, 116.13, 35.04, 32.16. High-resolution mass spectrometry (HRMS) (APCI) m/z: calcd for C_68_H_55_N_7_: 969.4519; found: 969.4512.

**
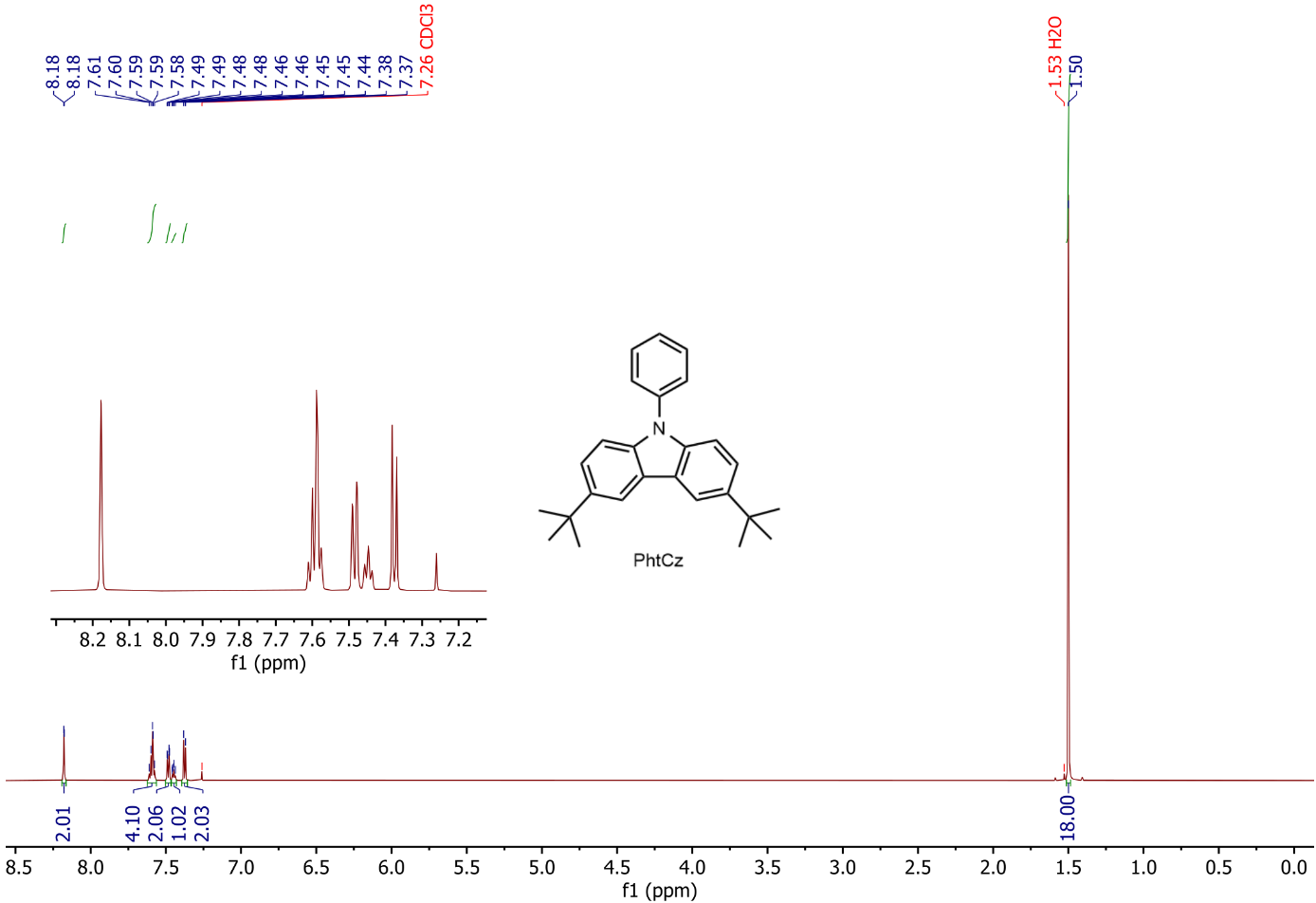
Fig. S1** ^1^H NMR spectrum of compound PhtCz (700 MHz, CDCl_3_).

**
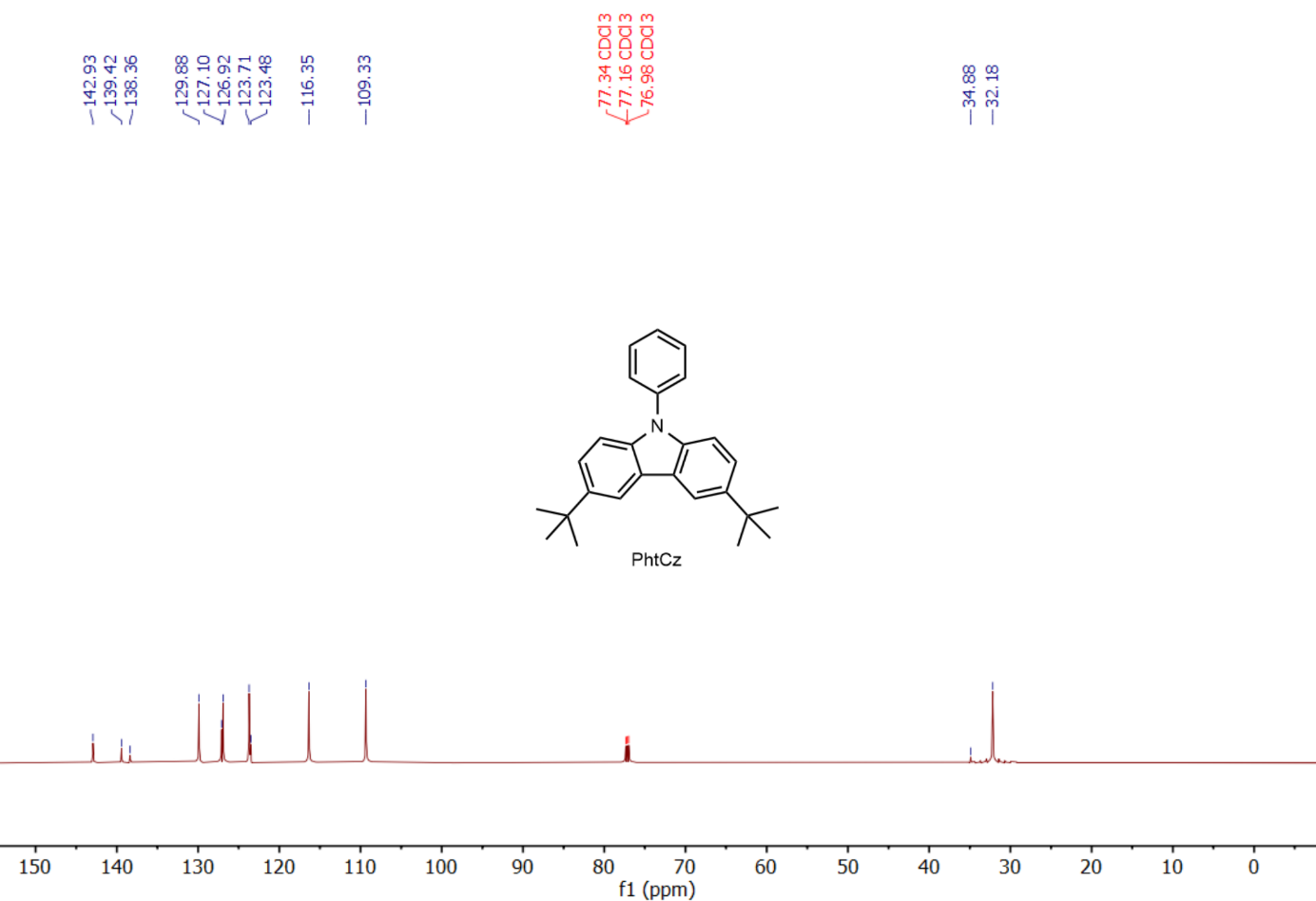
** **Fig. S2** ^13^C NMR spectrum of compound PhtCz (176 MHz, CDCl_3_).

**
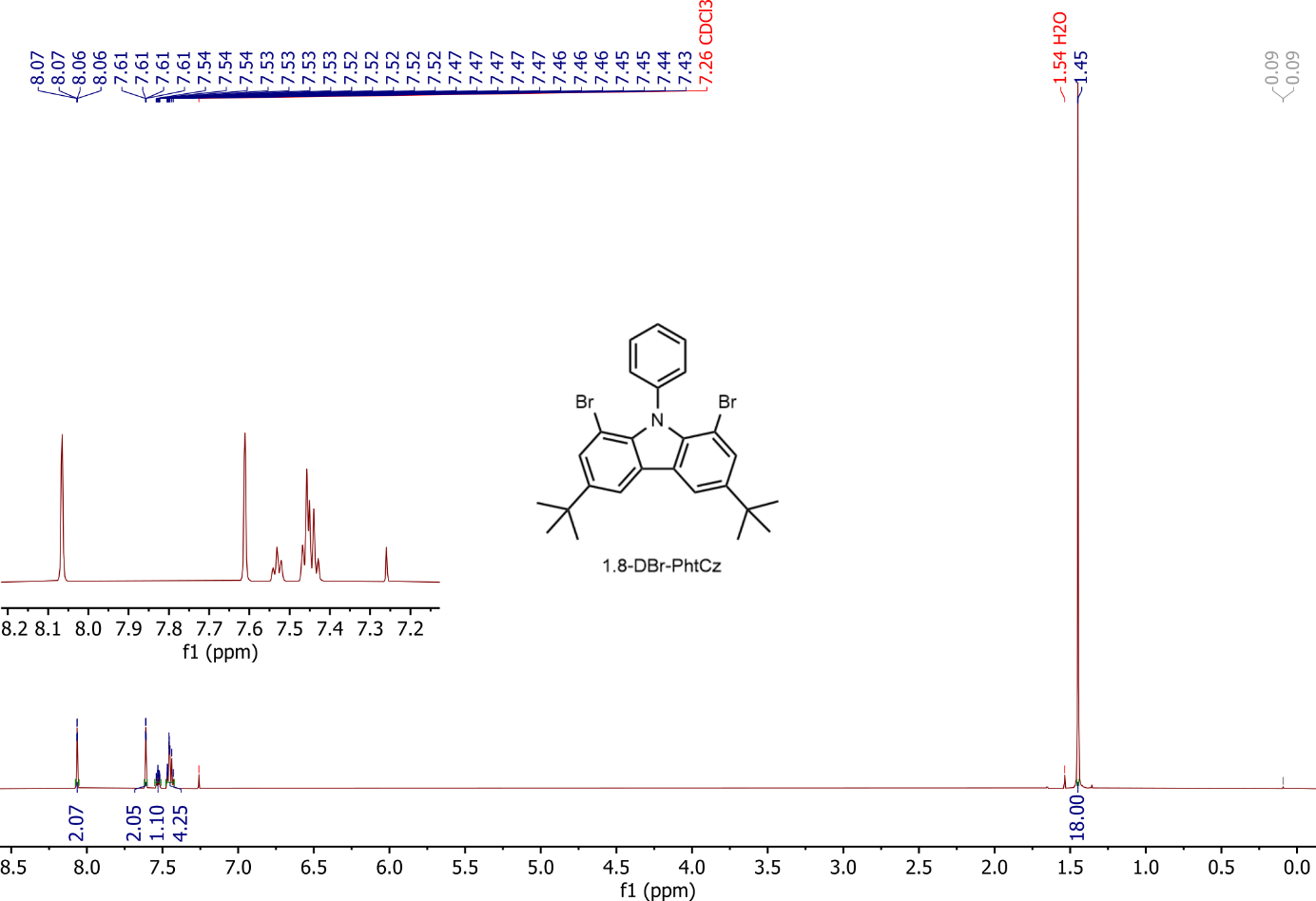
** **Fig. S3** ^1^H NMR spectrum of compound 1.8-DBr-PhtCz (700 MHz, CDCl_3_).

**
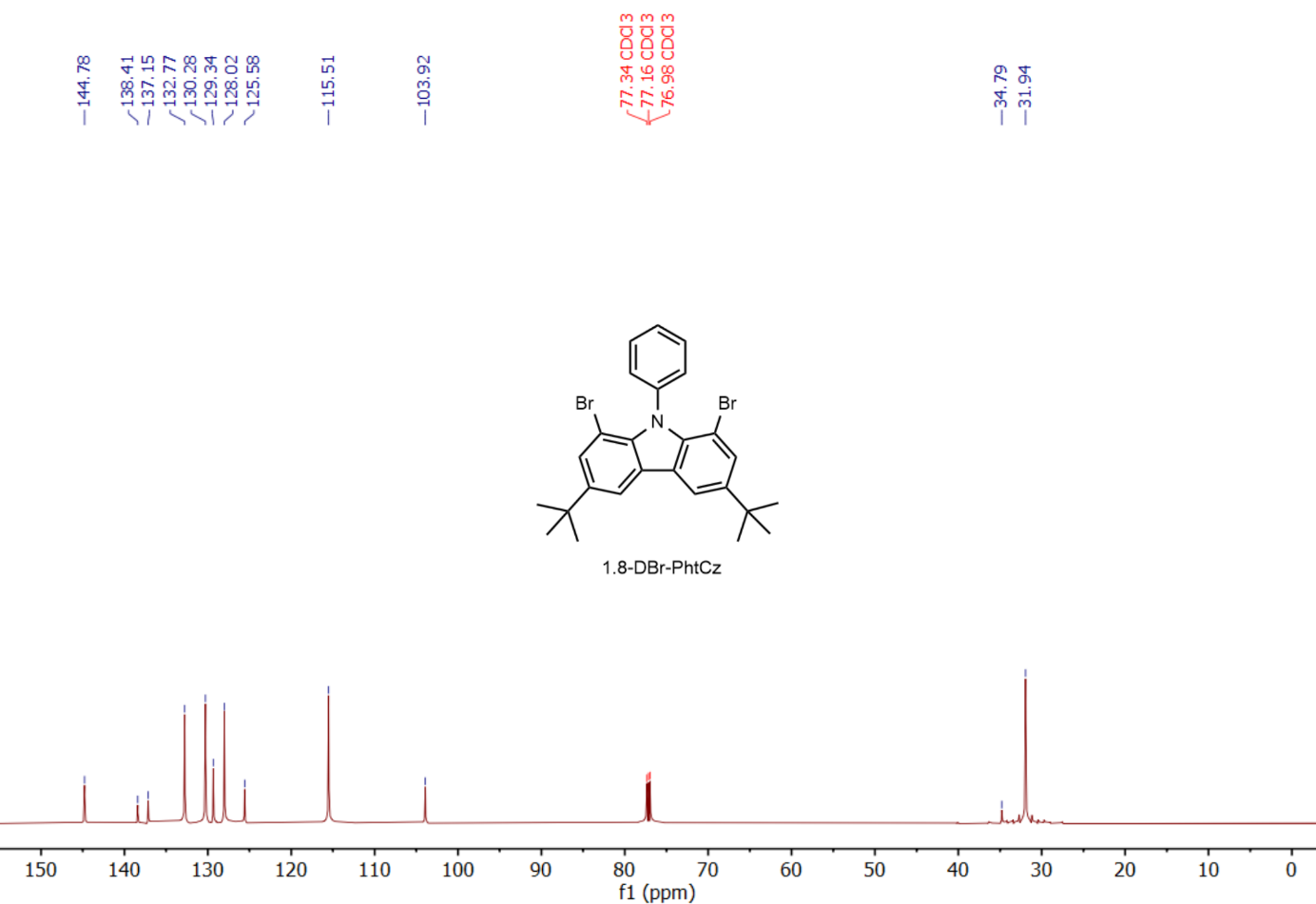
** **Fig. S4** ^13^C NMR spectrum of compound 1.8-DBr-PhtCz (176 MHz, CDCl_3_).


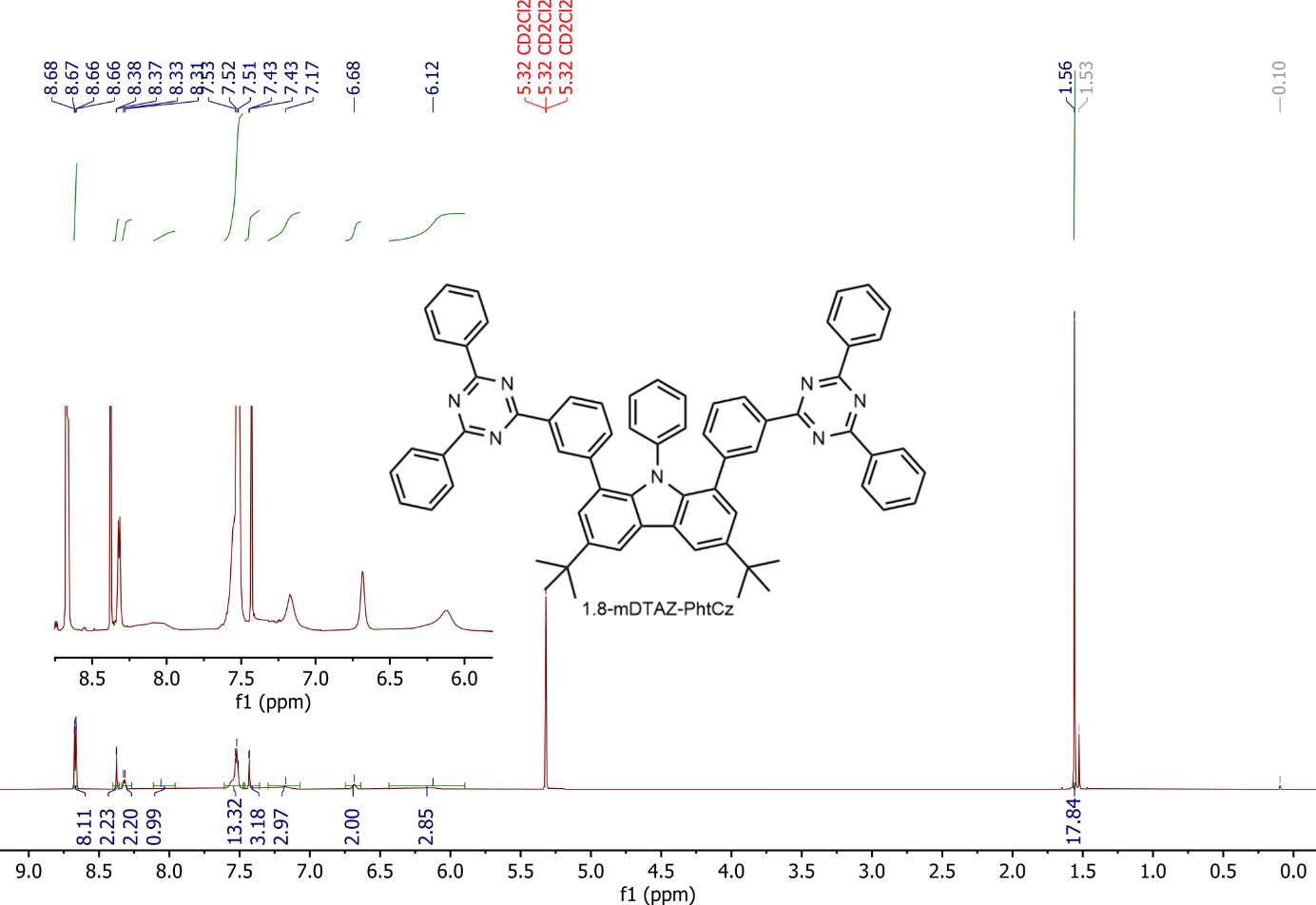


**Fig. S5** ^1^H NMR spectrum of compound 1.8-mDTAZ-PhtCz (700 MHz, CD_2_Cl_2_).

**
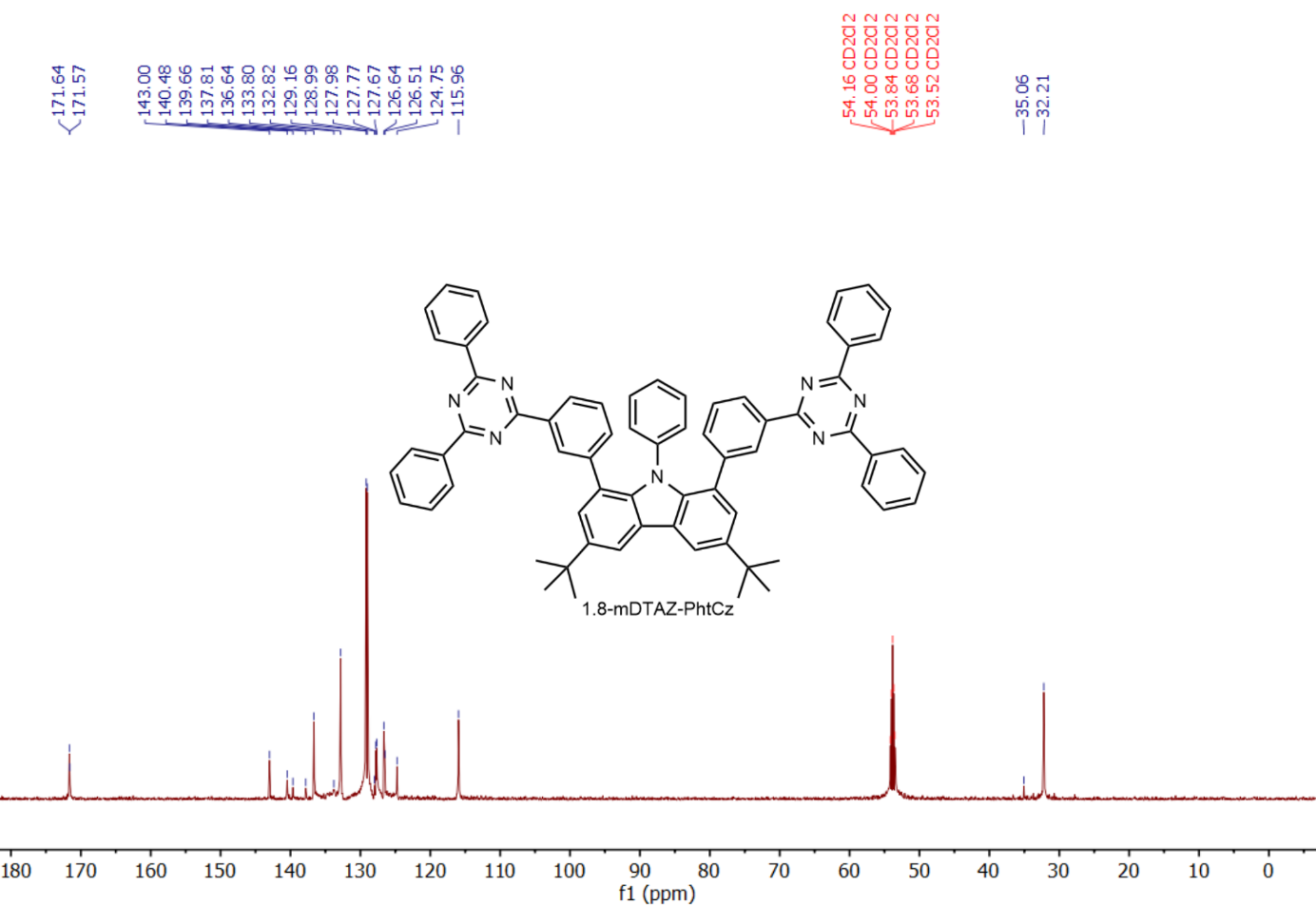
** **Fig. S6** ^13^C NMR spectrum of compound 1.8-mDTAZ-PhtCz (176 MHz, CD_2_Cl_2_).


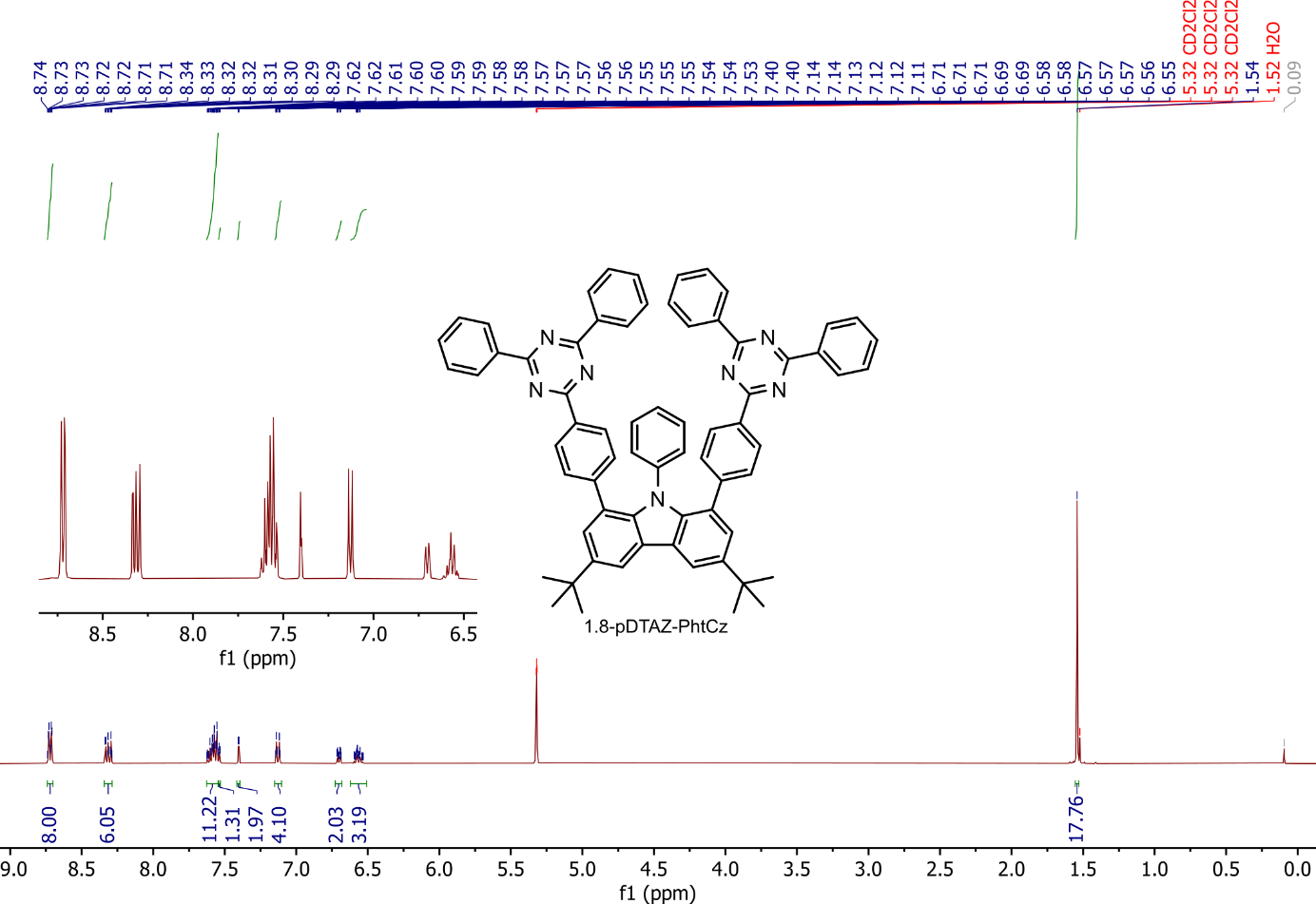


**Fig. S7** ^1^H NMR spectrum of compound 1.8-pDTAZ-PhtCz (400 MHz, CD_2_Cl_2_).


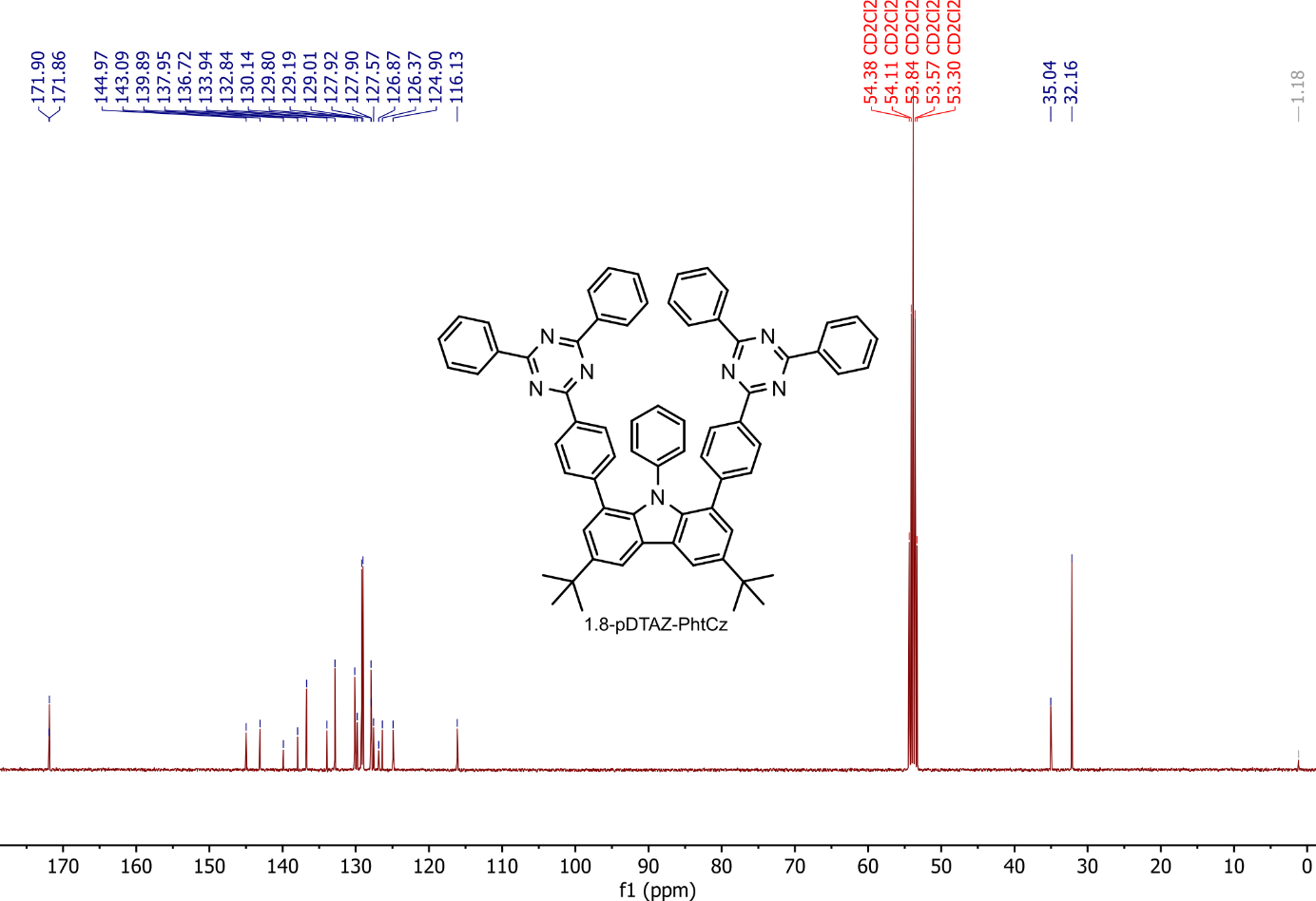


**Fig. S8** ^13^C NMR spectrum of compound 1.8-pDTAZ-PhtCz (101MHz, CD_2_Cl_2_).


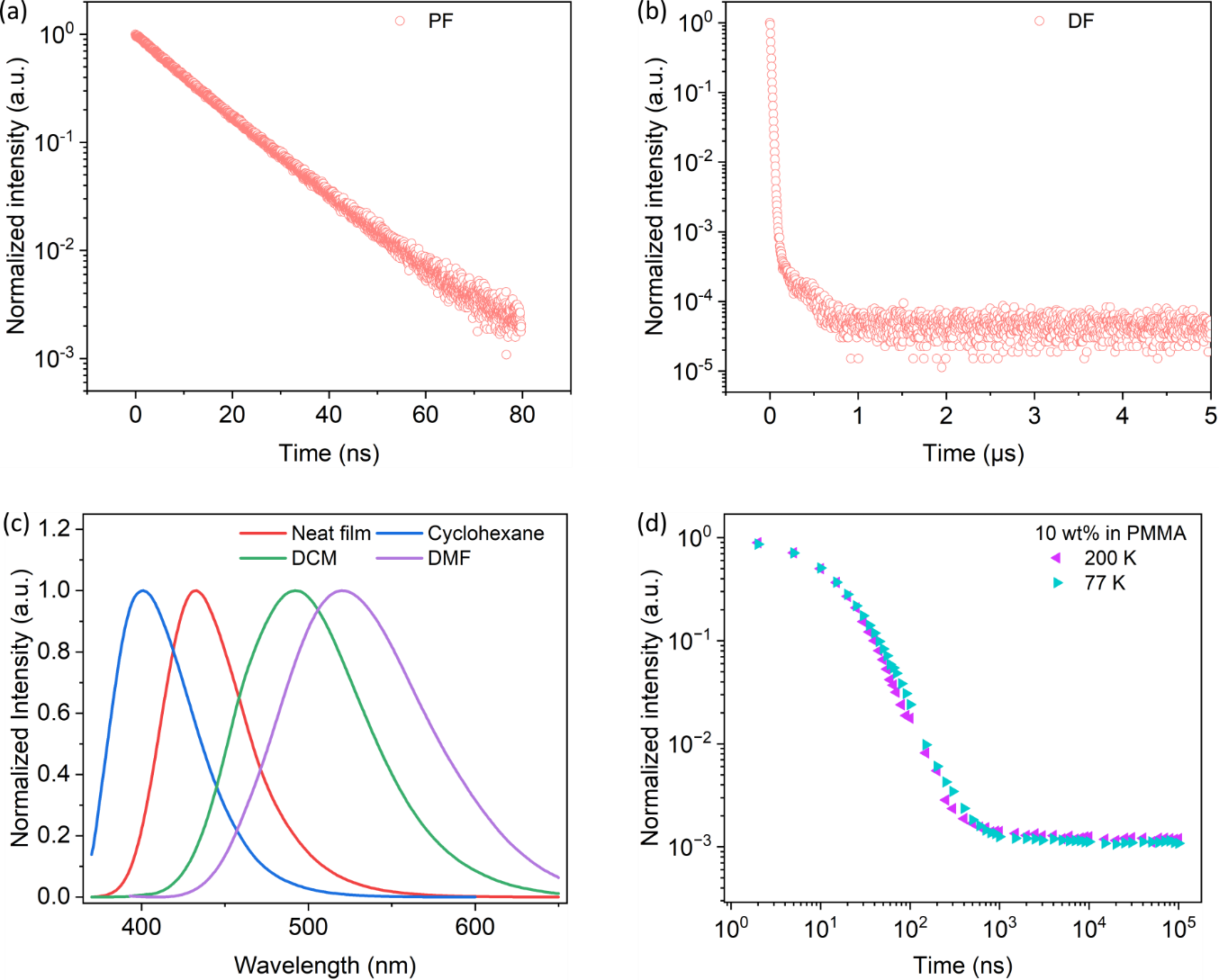


**Fig. S9** PL emission of 1.8-pDTAZ-PhtCz. (a) Prompt fluorescence in degassed toluene. (b) delayed fluorescence in degassed toluene. (c)Steady-state PL spectra in different solvents and neat film of 1.8-mDTAZ-PhtCz with the excitation at 360 nm. (d)Temperature dependent transient PL emission of 1.8-mDTAZ-PhtCz doped in PMMA at 200 K and 77 K.

**Table S1** Key photophysical properties of 1.8-mDTAZ-PhtCz.

| λ_PL_  (nm) ^a^ | λ_PL_  (nm) ^b^ | λ_PL_  (nm) ^c^ | λ_PL_  (nm) ^d^ |
| --- | --- | --- | --- |
| 410 | 433 | 493 | 521 |

1. In cyclohexane.
2. Neat film.
3. In DCM.
4. In DMF.


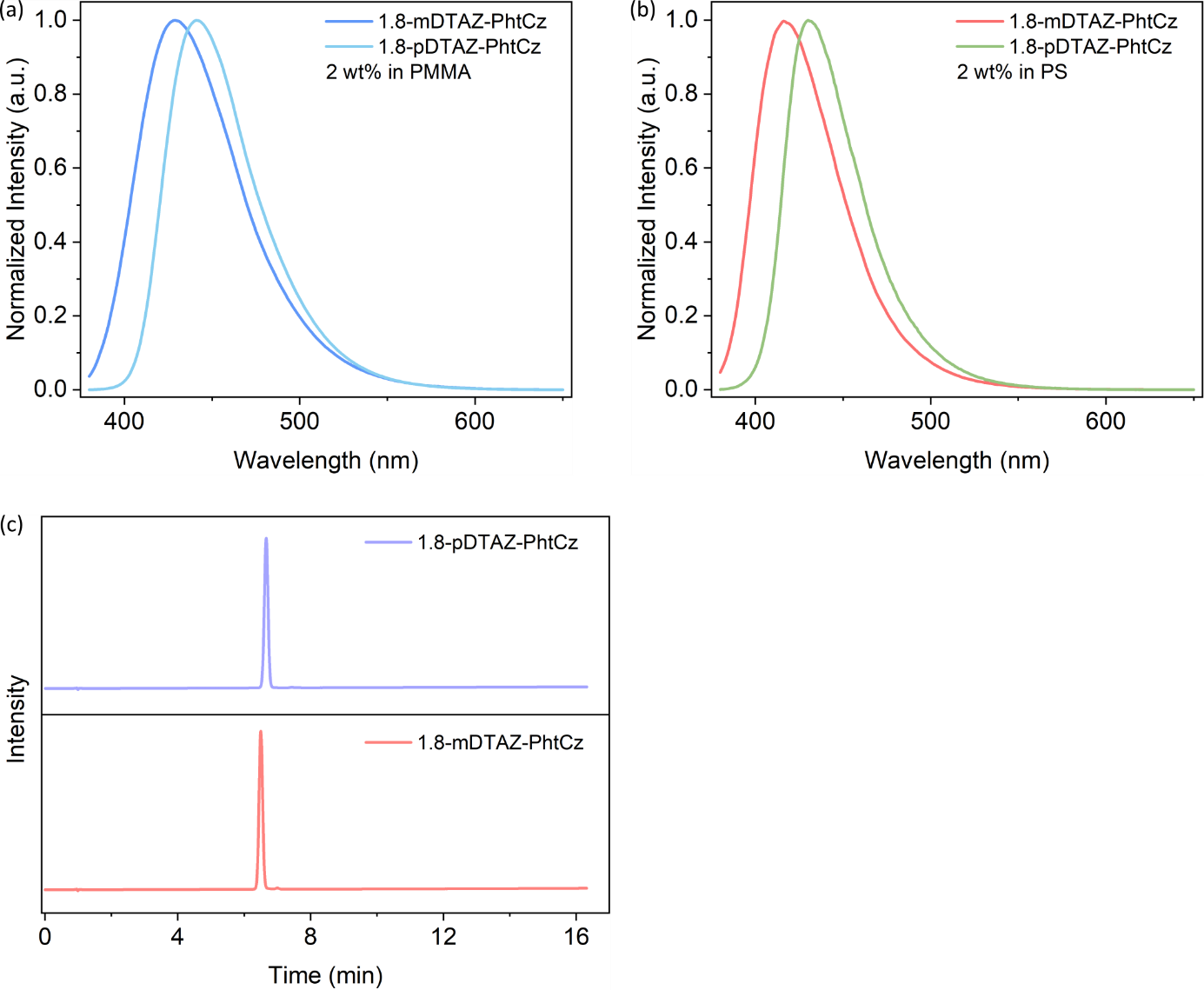


**Fig. S10** (a) PL spectra of both dopants in PMMA at a concentration of 2 wt% with the excitation at 360nm. (b) PL spectra of both dopants in PS at a concentration of 2 wt% with the excitation at 360nm. (c) HPLC for the two developed organic emitters.


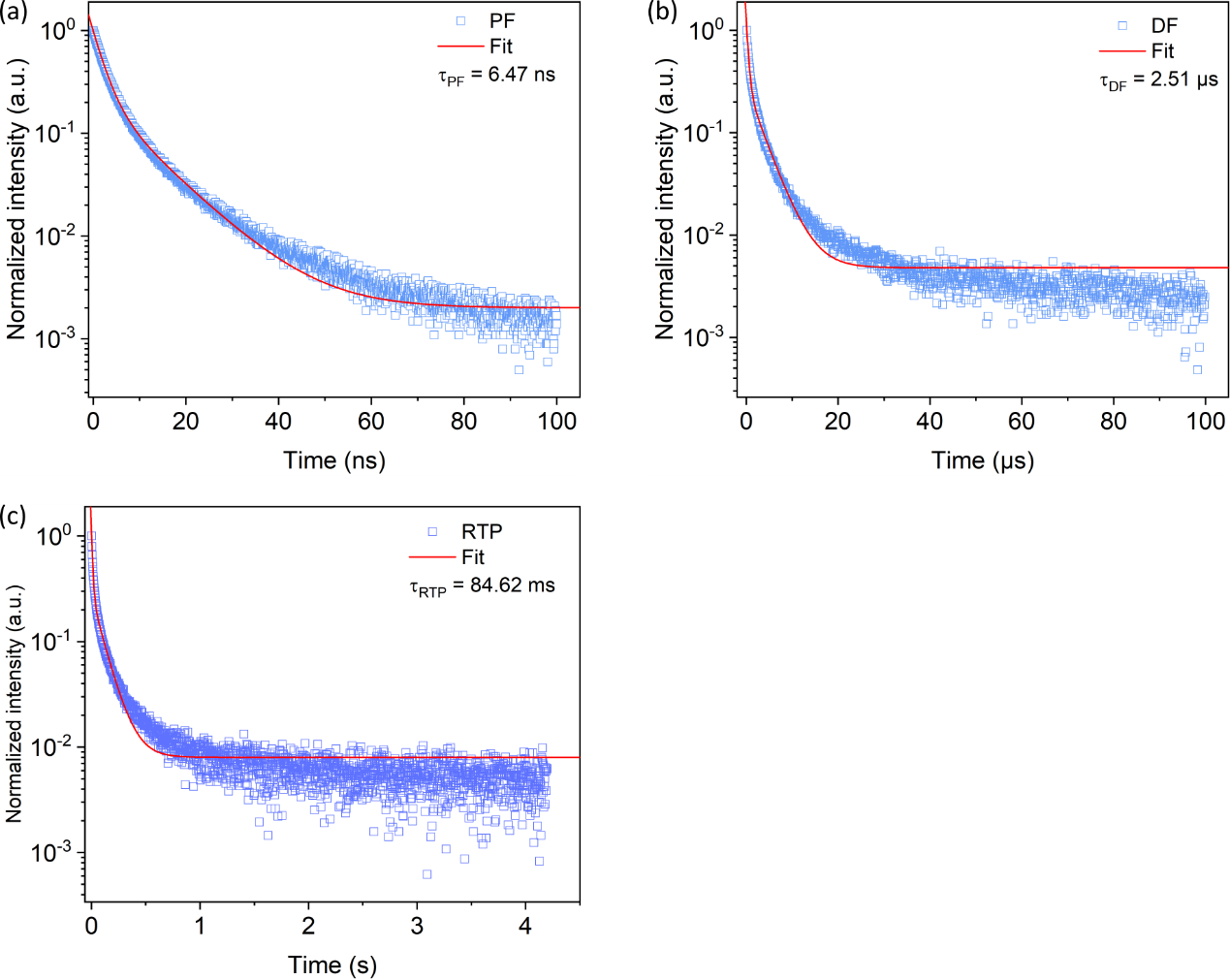


**Fig. S11** Fitting of the PF, TADF and RTP decay lifetimes of the model emitter in PMMA (2 wt%).

**Table S2** Lifetime estimation from transient PL decay of 1.8-mDTAZ-PhtCz in PMMA (2 wt%).

|  | A_1_ | t_1_ | A_2_ | t_2_ | ꚍ |
| --- | --- | --- | --- | --- | --- |
| PF | 0.83 | 2.34 ns | 0.23 | 9.99 ns | 6.47 ns |
| DF | 0.95 | 0.45 μs | 0.26 | 3.48 μs | 2.51 μs |
| RTP | 1.01 | 0.01 S | 0.27 | 0.11 S | 84.62 ms |


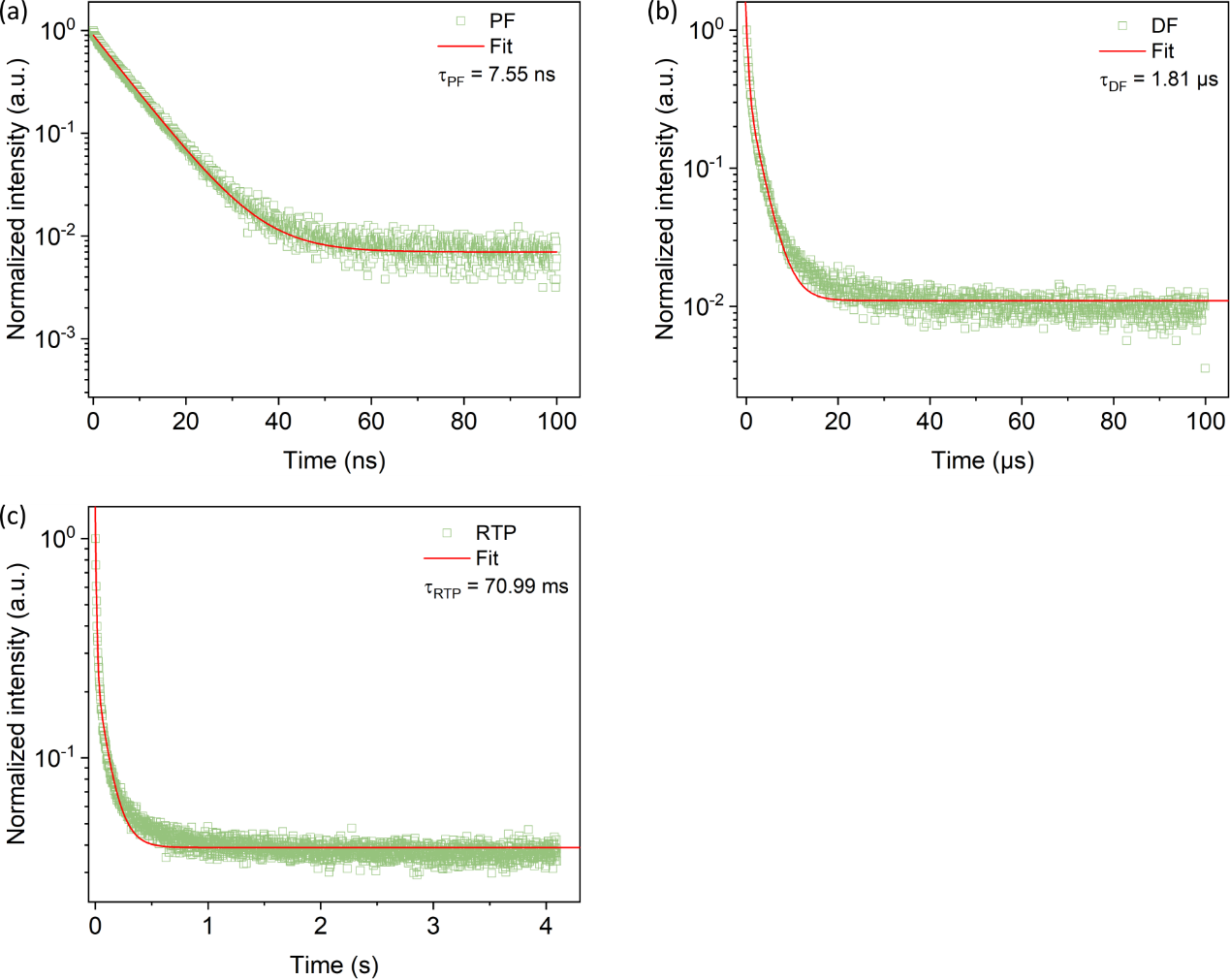


**Fig. S12** Fitting of the PF, TADF and RTP decay lifetimes of the model emitter in PMMA (0.5 wt%).

**Table S3** Lifetime estimation from transient PL decay of 1.8-mDTAZ-PhtCz in PMMA (0.5 wt%).

|  | A_1_ | t_1_ | A_2_ | t_2_ | ꚍ |
| --- | --- | --- | --- | --- | --- |
| PF | 0.99 | 7.55 ns | - | - | 7.55 ns |
| DF | 1.52 | 0.43 μs | 0.38 | 2.62 μs | 1.81 μs |
| RTP | 1.00 | 0.01 s | 0.21 | 0.10 s | 70.99 ms |


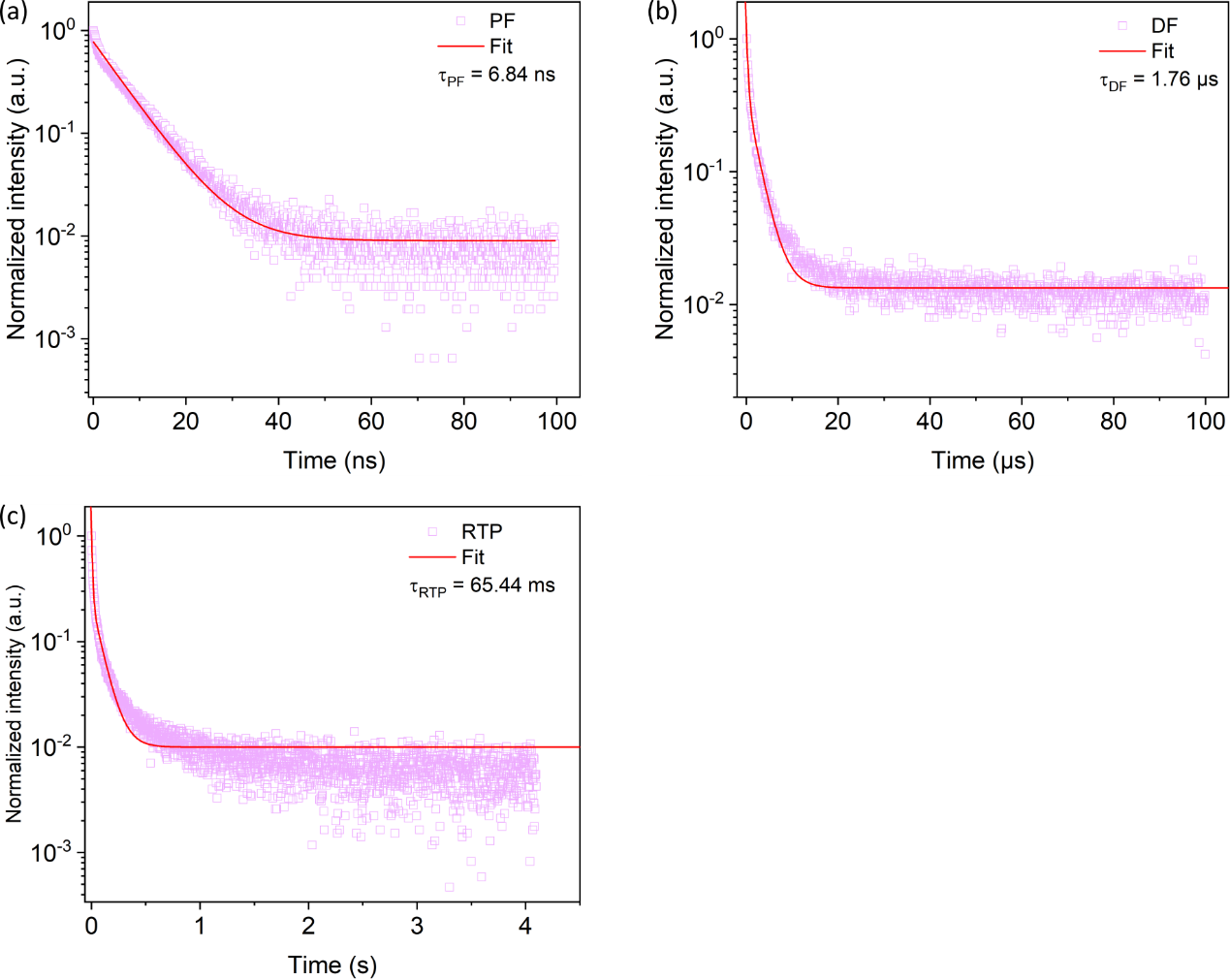


**Fig. S13** Fitting of the PF, TADF and RTP decay lifetimes of the model emitter in PS (2 wt%).

**Table S4** Lifetime estimation from transient PL decay of 1.8-mDTAZ-PhtCz in PS host at room temperature.

|  | A1 | t1 | A2 | t2 | ꚍ |
| --- | --- | --- | --- | --- | --- |
| PF | 1.03 | 6.84 ns | - | - | 6.84 ns |
| DF | 1.17 | 0.35 μs | 0.38 | 2.40 μs | 1.76 μs |
| RTP | 0.95 | 0.009 s | 0.23 | 0.89 s | 65.44 ms |


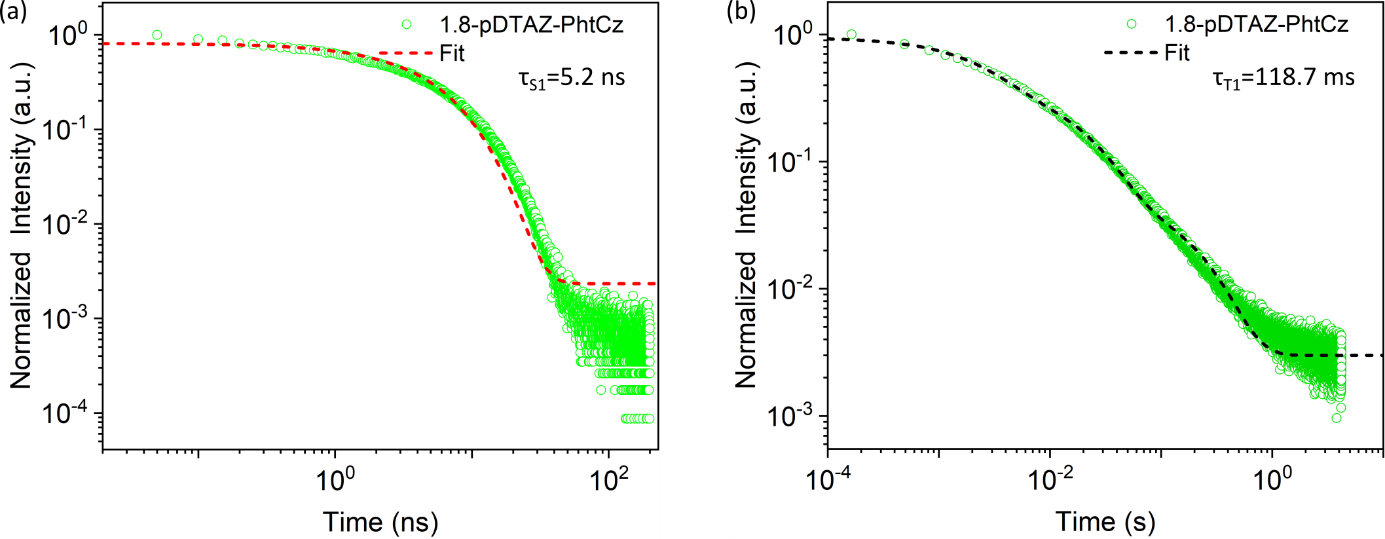


**Fig. S14** Transient PL emission of 1.8-pDTAZ-PhtCz. The doping concentration in PMMA is 2 wt%. (a) Normalized fluorescence decay transients at room temperature with a detection wavelength of 441 nm, fitted with the mono-exponential function. (b) Normalized phosphorescence decay transients at room temperature with a detection wavelength of 523 nm, fitted with the tri-exponential function.


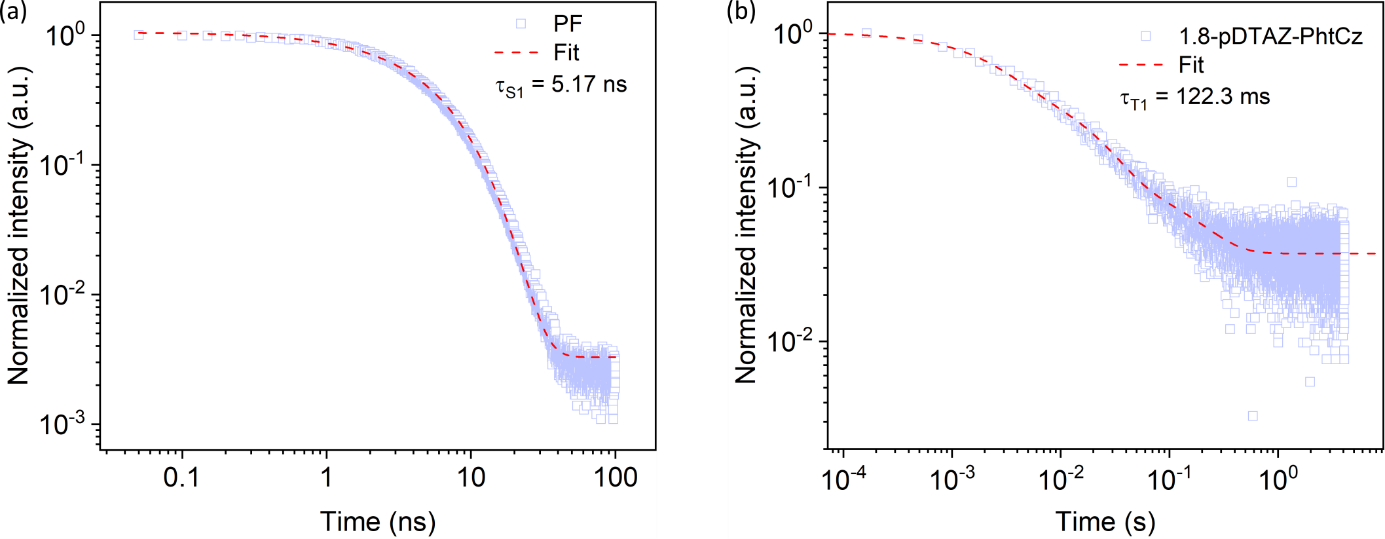


**Fig. S15** Fitting of the PF, and RTP decay lifetimes of the 1.8-pDTAZ-PhtCz in PS (2 wt%).

**Table S5** Kinetic parameters estimation from transient PL decay of 1.8-pDTAZ-PhtCz.

| τ_S1_  (s) | *k*_ISC_  (s^-1^) ^a^ | *k*_ISC_  (s^-1^)^b^ | τ_T1_  (ms) |
| --- | --- | --- | --- |
| 5.2 ×10^-9^ | 6.5×10^7^ | 8.5×10^7^ | 118.7 |

*k*_ISC_ =*φ*_ISC_ / τ_S1._

1. *k*_nr.PF_ =0, *φ*_nr.PF_ =0, *φ*_ISC_ =1- *φ*_PF_
2. *k*_nr.PH_ =0, *φ*_nr.PH_ =0, *φ*_ISC_ =*φ*_PH_


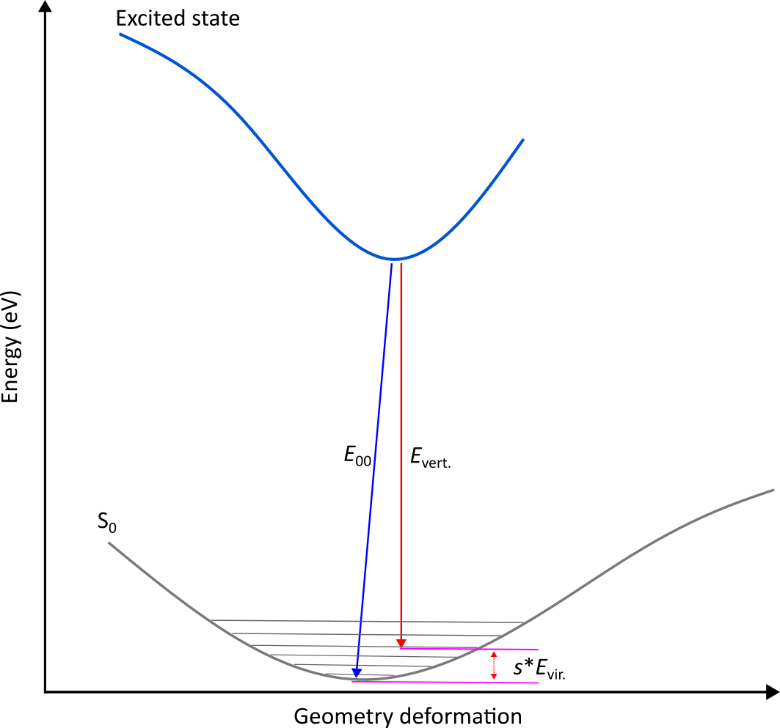


**Fig. S16** Physical quantities related for the spectral lineshape analysis in the Jablonski diagram.

For PF and RTP emission, the different vibronic energy states in the ground state can be described with a small energy difference, while the Franck−Condon factors describing the transition probabilities to different vibronic states (i.e. these Gaussian shape fitting components) follow a Poisson progression, which leads to the relation between spectral lineshape and energetic states involved:

$\frac{I\left( E \right)}{E^{3}}=a\sum_{n=0}^{\infty} \left( \frac{s^{n}}{n!}e^{-s} \right)\left[ \exp\left( -\frac{\left( E-\left( E_{\mathrm{vert}}+s*E_{\mathrm{vibr}}-n*E_{\mathrm{vibr}} \right) \right)^{2}}{2\sigma_{n}^{2}} \right) \frac{1}{\sigma_{n}\sqrt{2\pi}} \right]$ .

In the above equation, *a* is a pre-factor, *s* the Huang-Rhys factor, *E*_vibr_ the energy difference between the vibronic states nearby. The first vibronic band peak energy difference *E*_00_ between the excited state and the ground state can be determined by:

${E_{00}=E}_{\mathrm{vert}}+s*E_{\mathrm{vibr}}$ .

The vertical transition energy difference *E*_vert_ and *E*_00_ during the transition is schematically shown in **Fig. S16**.

**Table S6** Energy levels determined from different methods and the molecular simulation results.

|  | Sample | *s* (S_1_/T_1_) | *E*_00_ (S_1_/T_1_) | *E*_vir._ (S_1_/T_1_) | *E*_vert._ (S_1_/T_1_) | *E*_peak_(S_1_/T_1_) |
| --- | --- | --- | --- | --- | --- | --- |
| Exp. | 1.8-mDTAZ-PhtCz | 0.683 / 2.466 | 2.985 / 2.945 | 0.145 / 0.145 | 2.887 / 2.82 | 2.904 / 2.707 |
|  | 1.8-pDTAZ-PhtCz | 1.510 / 2.632 | 2.881 / 2.578 | 0.127 / 0.145 | 2.689 / 2.196 | 2.812 / 2.371 |
| Sim. | 1.8-mDTAZ-PhtCz |  | 2.978 / 2.912 |  |  |  |
|  | 1.8-pDTAZ-PhtCz |  | 2.669 / 2.554 |  |  |  |


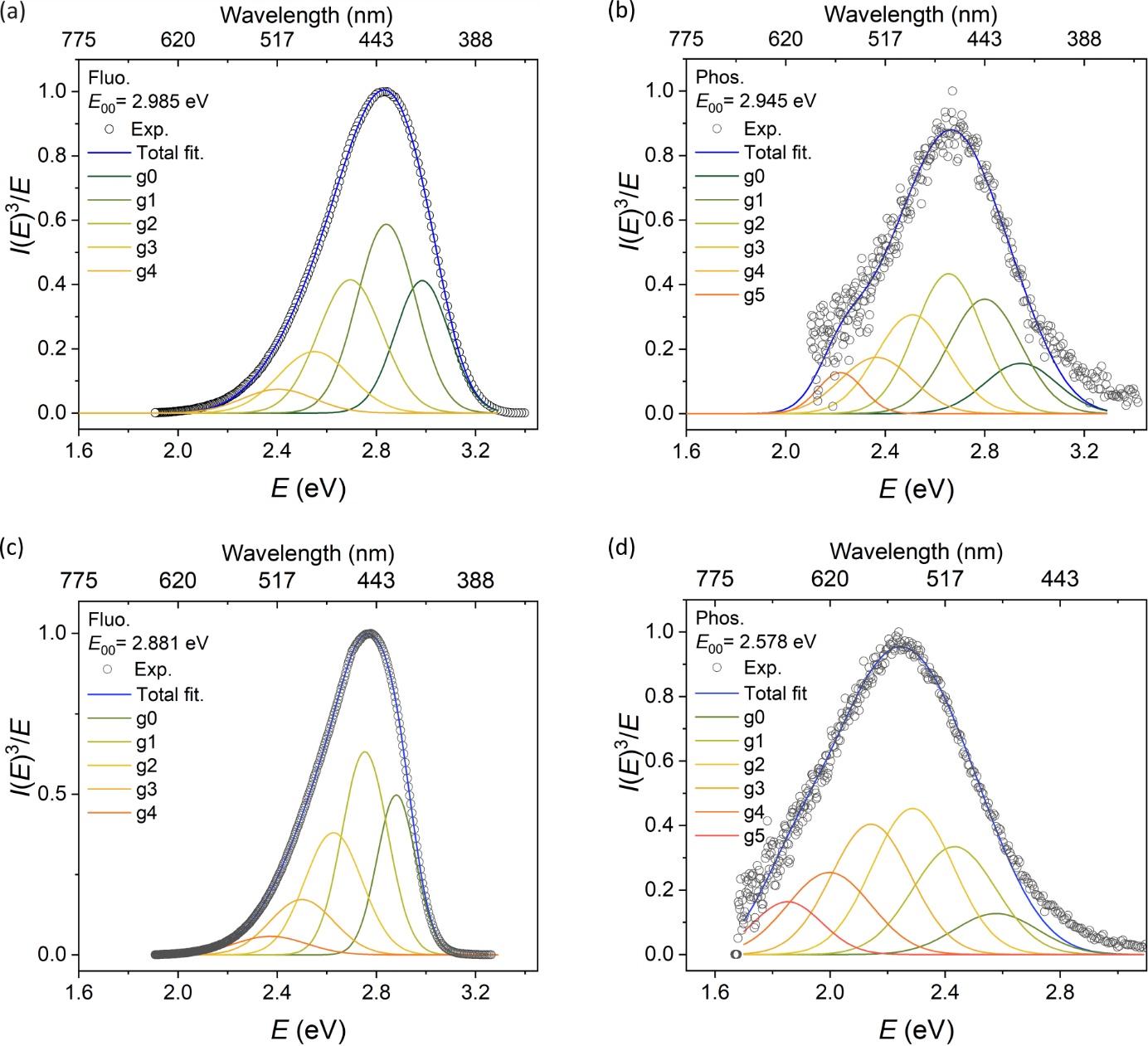


**Fig. S17** Lineshape analysis. Fluorescence spectra (steady-state PL in PMMA host measured at ambient condition with a laser excitation at 360 nm.) of (a) 1.8-mDTAZ-PhtCz and (c) 1.8-pDTAZ-PhtCz. Phosphorescence spectra (delayed spectra measured at room temperature with a laser excitation at 360 nm) of (b) 1.8-mDTAZ-PhtCz. (d) 1.8-pDTAZ-PhtCz.

# 2. X-ray crystal structure analysis and crystal imaging

The crystals of 1.8-mDTAZ-PhtCz and 1.8-pDTAZ-PhtCz were grown by slow evaporation in dichloromethane and methanol. The X-ray crystallographic coordinates for the molecular structure in this study have been deposited at the Cambridge Crystallographic Data Centre (CCDC), under deposition numbers 2236406 (1.8-mDTAZ-PhtCz) and 2287999 (1.8-pDTAZ-PhtCz). These data can be obtained free of charge from the Cambridge Crystallographic Data Centre “http://www.ccdc.cam.ac.uk/data_request/cif”.

As shown in **Fig. S19**, for molecules 1.8-pDTAZ-PhtCz, the diphenyltriazine group tended towards a planar conformation due to strong intramolecular hydrogen bonding constraints. The torsion angle between the triazine molecule and the tert-butylcarbazole in 1.8-pDTAZ-PhtCz is almost identical to that in 1.8-mDTAZ-PhtCz. In the crystal unit of 1.8-pDTAZ-PhtCz, two molecules are closely stacked and show a head-to-tail staggered arrangement. The closest distance from the tert-butyl carbazolyl to the N-phenyl ring of the other molecule is 3.91 Å. The detailed crystal parameters summarized in **Table S8**. As shown in **Fig. S20**, single crystals of 1.8-pDTAZ-PhtCz, similar to 1.8-mDTAZ-PhtCz, exhibit ultra-long afterglow emission.


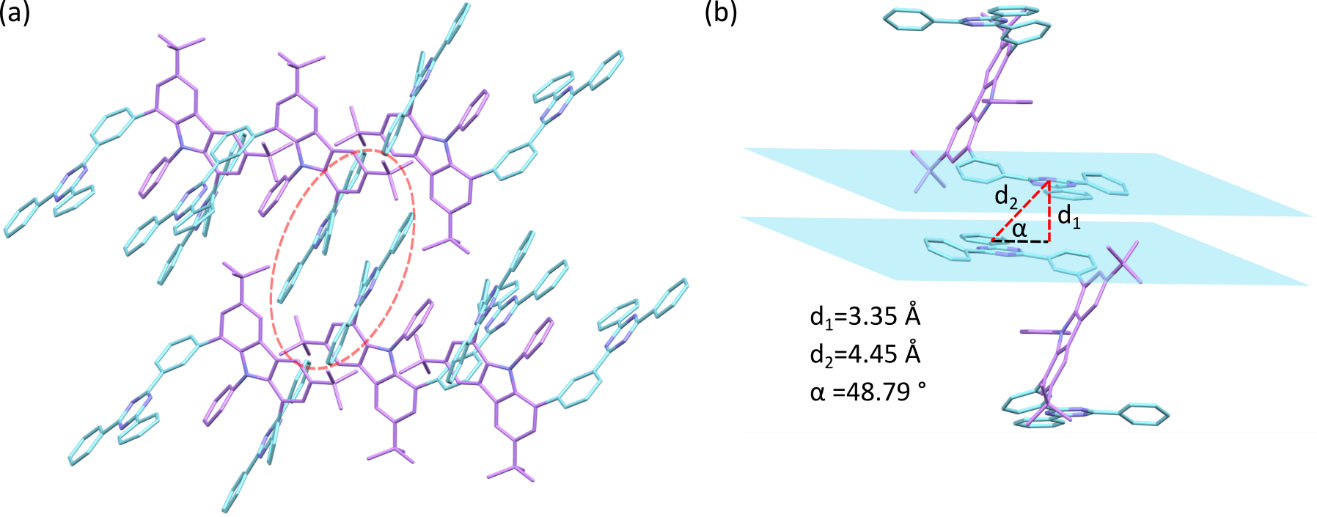


**Fig. S18** (a) Intermolecular structure and (b) molecular packing behavior in a unit cell of 1.8-mDTAZ-PhtCz. The local packing shown in (b) are selected from the part in red cycles.


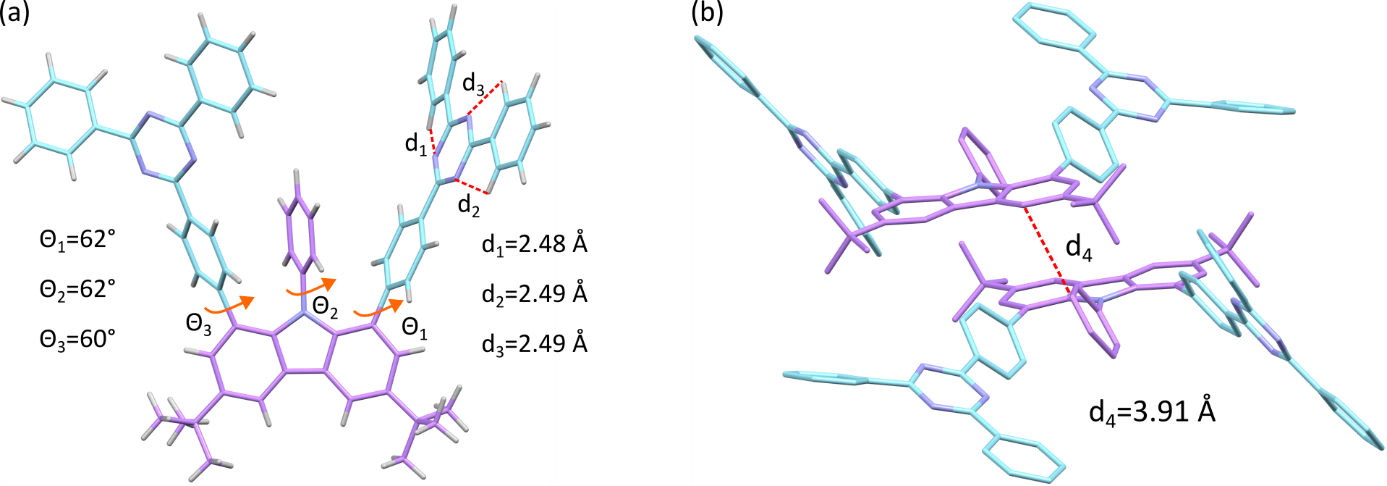


**Fig. S19** (a) Intramolecular interactions in the single crystal of 1.8-pDTAZ-PhtCz. (b) Molecular packing behavior in a unit cell 1.8-pDTAZ-PhtCz.


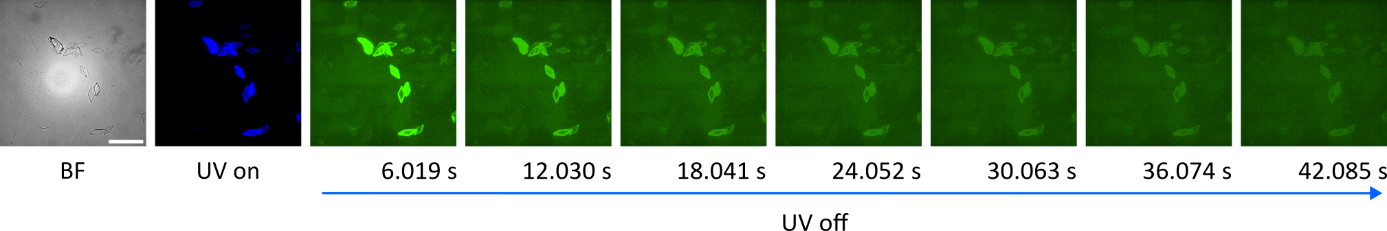


**Fig. S20** Ultralong afterglow emission from the 1.8-pDTAZ-PhtCz crystals.

**Crystallographic Data and Structure Refinement Details**

**Table S7**Crystal data and structure refinement for 1.8-mDTAZ-PhtCz (2236406).

| Empirical formula | C_72_H_63_Cl_8_N_7_ |  |
| --- | --- | --- |
| Moiety formula | C_68_H_55_N_7_, 4(CH_2_Cl_2_) |  |
| Formula weight | 1309.89 g/mol |  |
| Temperature | 120(2) K |  |
| Crystal system | Monoclinic |  |
| Space group name, number | C 2/c, (15) |  |
| Unit cell dimensions | a = 17.6557(15) Å |  |
|  | b = 17.4013(8) Å | β = 108.245(6)° |
|  | c = 22.2560(17) Å |  |
| Volume | 6494.0(8) Å^3^ |  |
| Z | 4 |  |
| Density (calculated) | 1.340 mg/m^3^ |  |
| Absorption coefficient | 0.396 mm^-1^ |  |
| F(000) | 2720 |  |
| Crystal size | 0.060 x 0.120 x 0.900 mm^3^ |  |
| Wavelength, radiation type | 0.71073 Å, MoKα |  |
| Theta range for data collection | 2.531 to 28.067° |  |
| Index ranges | -22<=h<=23, -22<=k<=22, -29<=l<=29 | |
| Reflections collected | 17827 |  |
| Independent reflections | 7771 [R(int) = 0.0373] |  |
| Data/restraints/parameters | 7771 / 6 / 401 |  |
| Goodness-of-fit on F^2^ | 1.082 |  |
| Final R indices [I>2sigma(I)] | R1 = 0.0775, wR2 = 0.1854 |  |
| Final R indexes [all data] | R1 = 0.1153, wR2 = 0.2163 |  |
| Largest diff. peak and hole | 0.712 / -0.819 eÅ^-3^ |  |

**Table S8**Crystal data and structure refinement for 1.8-pDTAZ-PhtCz (2287999).

| Empirical formula | C_68_H_55_N_7_ |  |
| --- | --- | --- |
| Moiety formula | C_68_H_55_N_7_ |  |
| Formula weight | 970.19 g/mol |  |
| Temperature | 120(2) K |  |
| Crystal system | Triclinic |  |
| Space group name, number | P -1, (2) |  |
| Unit cell dimensions | a = 9.6915(3) Å | α = 75.474(2)° |
|  | b = 16.8118(4) Å | β = 79.839(2)° |
|  | c = 17.3312(5) Å | γ = 73.930(2)° |
| Volume | 2609.29(13) Å^3^ |  |
| Z | 2 |  |
| Density (calculated) | 1.235 mg/m^3^ |  |
| Absorption coefficient | 0.564 mm^-1^ |  |
| F(000) | 1024 |  |
| Crystal size | 0.070 x 0.070 x 0.160 mm^3^ |  |
| Wavelength, radiation type | 1.54178 Å, CuKα |  |
| Theta range for data collection | 5.606 to 67.934° |  |
| Index ranges | -11<=h<=11, -20<=k<=20, -20<=l<=20 | |
| Reflections collected | 29054 |  |
| Independent reflections | 8982 [R(int) = 0.0297] |  |
| Data/restraints/parameters | 8982 / 12 / 696 |  |
| Goodness-of-fit on F^2^ | 1.060 |  |
| Final R indices [I>2sigma(I)] | R1 = 0.0620, wR2 = 0.1456 |  |
| Final R indexes [all data] | R1 = 0.0835, wR2 = 0.1662 |  |
| Largest diff. peak and hole | 0.376 / -0.259 eÅ^-3^ |  |

# 3. Supplementary note S1: Three-level model

**
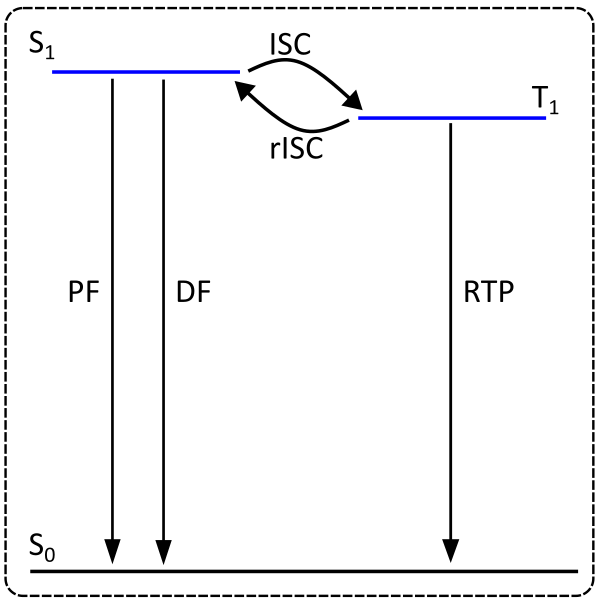
**

**Scheme S2** A three-level model for simultaneous PF, TADF, and RTP emission from a single emitter.

From Scheme S2, the singlet density *n*_S_ and triplet density *n*_T_ as a function of time (*t*) can be described by two coupled ordinary differential equations (coupled ODEs):

$\frac{dn_{S}}{dt}=-\frac{n_{S}}{\tau_{S}}-k_{\mathrm{ISC}}n_{S}+k_{\mathrm{rISC}}n_{T}$ (Eq. S1)

$\frac{dn_{T}}{dt}={-\frac{n_{T}}{\tau_{T}}+k}_{\mathrm{ISC}}n_{S}-k_{\mathrm{rISC}}n_{T}$ (Eq. S2)

In the above equations, *n*_S_ and *n*_T_ denote the singlet and triplet lifetime. Here, the bimolecular annihilation effect is not included. This is reasonable when the exciton density is low. For emitters with both TADF from singlet and RTP radiation from triplet, the transient PL decay is therefore the sum of *n*_S_ and *n*_T_ as a function of time:

$n=n_{S}+n_{T}$. (Eq. S3)

The non-radiative losses in singlets or triplets, contributes to the exciton lifetime:

$\tau_{(S,T)}=\frac{1}{k_{r}+k_{\mathrm{nr}}}$. (Eq. S4)

It has been demonstrated that the analytical formula for the singlet decay should be in a form of bi-exponential. Thus, given the above-mentioned three-level model, the transient PL decay can only show two distinct lifetime. We here further investigate the underlying mechanism by solving these equations numerically. We started to assume an arbitrary low singlet exciton density as the initial condition. Such a treatment can make sure that the annihilation effect is not involved, so the rate equations are physically robust. The shape of the solution for Equation S1-S4 can be used to determine how many components can be observed in such a three-level system.

For the given coupled ODEs, six parameters are involved. One can drop the physical meaning for these parameters firstly, but just comparing the difference of the numerical solution to determine the solution properties. Observing the form of Eq. S1-S2, one can see that 1/τ_S_ and *k*_ISC_ have the same effect on the decay rate of *n*_S_ (thus: d*n*_S_/dt), and 1/τ_T_ and *k*_rISC_ have the same effect on the decay rate of *n*_T_ (d*n*_T_/d*t*). In other words, whenever the difference between 1/τ_S_ and *k*_ISC_, 1/τ_T,_ and *k*_rISC_ is large enough, such as more than 1 order difference, the term with a larger absolute number will dominate while the smaller term can be cancelled. Specifically for Eq. S2, this mathematical effect gives rise to an effect that only one term either 1/τ_T_ or *k*_rISC_ can dominate. Thus, only one major component will contribute to the decrease of *n*_T_ in the solution form. Physically, this effect means that triplets from a single energy level can only either contributes to one major process, either TADF with lifetime in 10^-6^ s or RTP emission with lifetime in 1 s.

The mathematical analysis can be demonstrated by scanning the kinetic parameters involved. Since it is known that the singlet lifetime for TADF emitters can be varied from 10^-9^ to 10^-7^ s range, while the long-lived RTP emission can have lifetime from 10^-3^ to 1 s, the solution for Eq. S1-S4 can be obtained with different combinations of kinetic parameters.

**
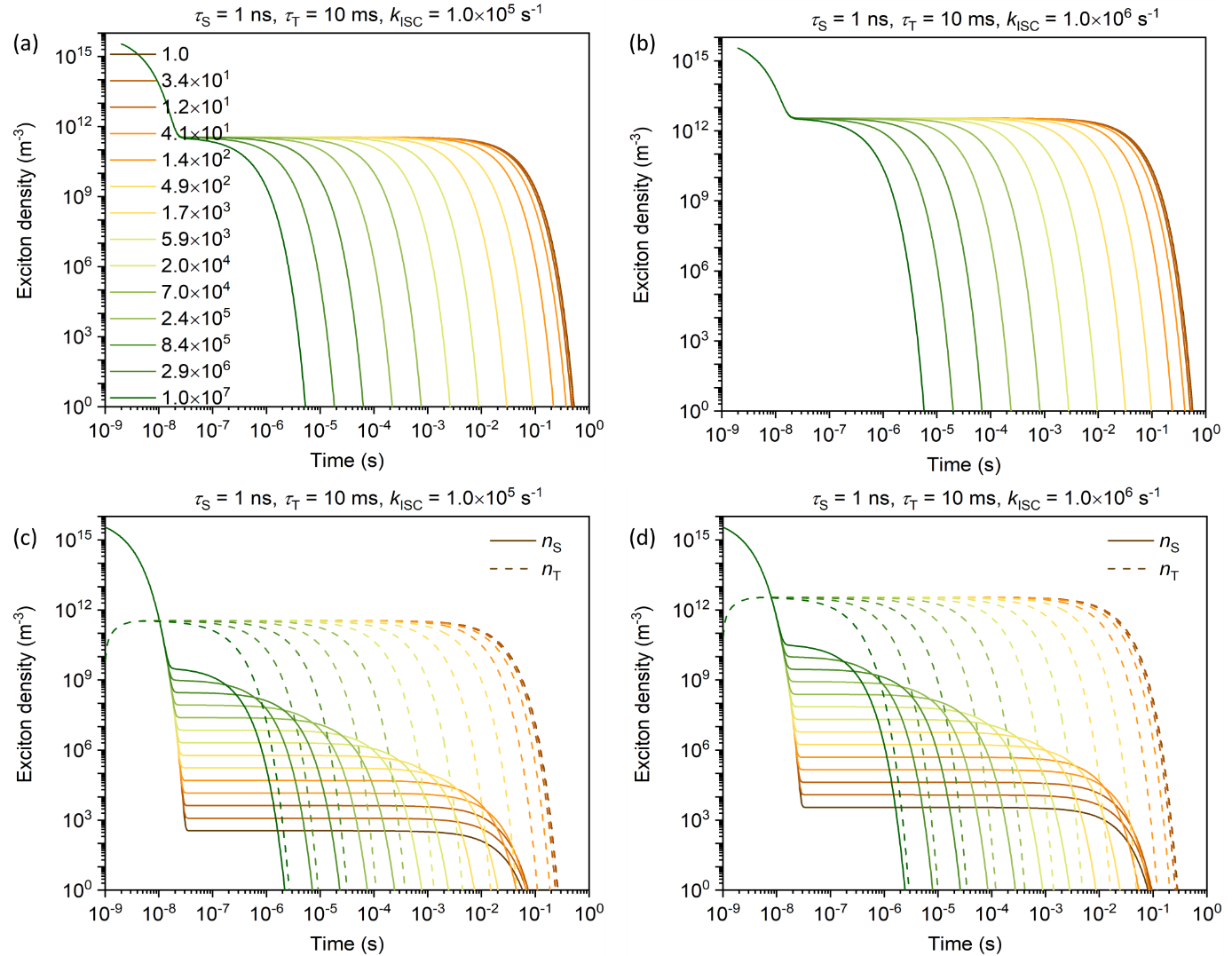
** **Fig. S21** Exciton density modelling with a three-level model. The τ_S_ is assumed to be 1 ns and τ_T_ 10 ms. The ISC rate is 10^5^ s^-1^ for (a) and (c), while it is 10^6^ s^-1^ for (b) and (d). For each plot, the rISC rate is swept from 1 to 10^7^ s^-1^ in the logarithmic scale. Top row: the emission decay (*n*_S_+*n*_T_); bottom row: emissive singlet and triplet density contributing to the entire PL decay.


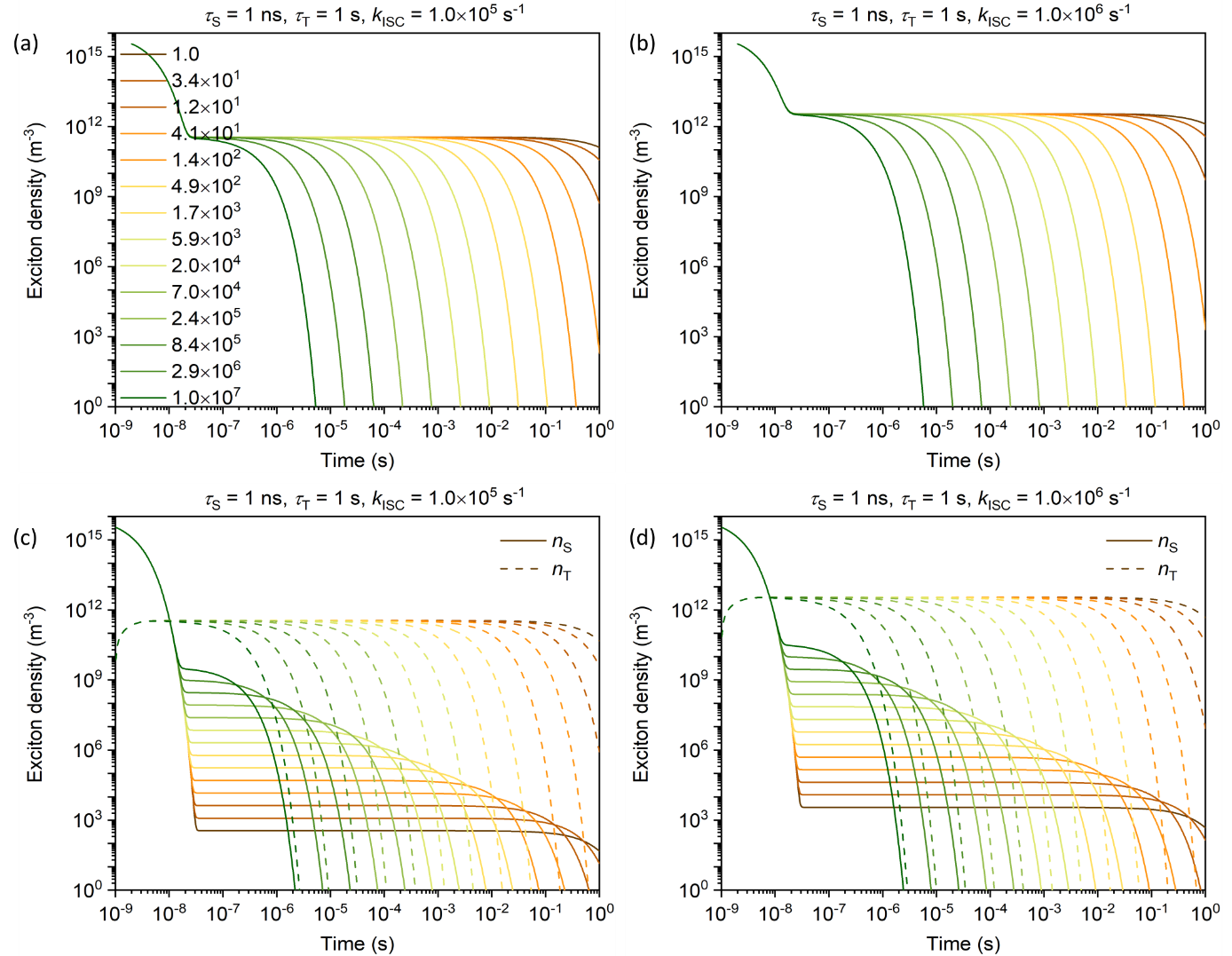
 **Fig. S22** Exciton density modelling with a three-level system. The τ_S_ is assumed to be 1 ns and τ_T_ 1 s. The ISC rate is 10^5^ s^-1^ in (a) and (c), while it is 10^6^ s^-1^ in (b) and (d). For each plot, the rISC rate is swept from 1 to 10^7^ s^-1^ in the logarithmic scale. Top row: the sum of emissive exciton density (*n*_S_+*n*_T_); bottom row: emissive singlet and triplet density contributing to the PL decay.

The contribution of each excited state can be examined by separating the singlet and triplet evolution, as shown in the bottom rows in **Fig. S21-S22**. When 1/τ_T_ is comparable or larger than *k*_rISC_, the long-lived decay intensity and lifetime is mainly determined by the triplet radiation as phosphorescence. Within the rate range when 1/τ_T_>*k*_rISC_, the change of *k*_rISC_ plays a negligible role on the PL decay. The TADF emission is vanished, leading to the observation of one delayed PL component as phosphorescence. When *k*_rISC_ increasing to a range which is comparable to 1/τ_T_, the delayed emission from singlets via rISC contributes to direct triplet emission. TADF emission starts to play a role for the total PL decay. However, in this specific case, since 1/τ_T_ and *k*_rISC_ is on the same order, still only one delayed component can be resolved.

**
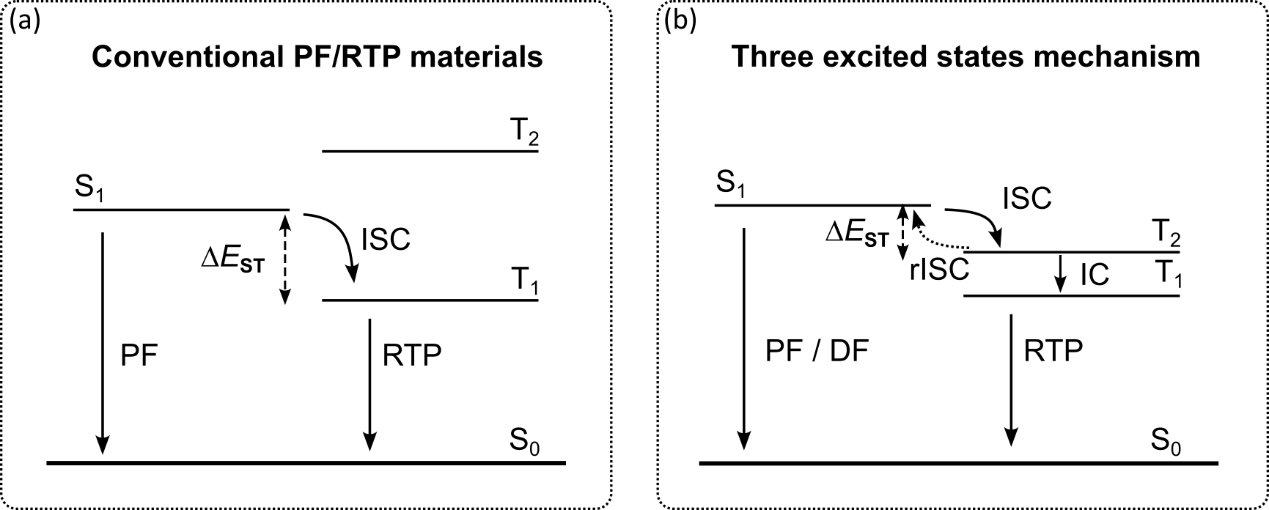
**

**Fig. S23** **Schematic illustration about the mechanism difference.** (a) Conventional PF/RTP materials; (b) PF/TADF/RTP materials with multiple excited states model in the current work.

# 4. Supplementary note S2: Four-level model

For a more comprehensive four-level mode with the consideration of (reverse) IC between T_2_ and T_1_, the exciton kinetics can be described as:

$\frac{{dn}_{S1}}{dt}=-\frac{n_{S1}}{\tau_{S1}}-k_{\mathrm{ISC}}n_{S1}+k_{rISC2}n_{T2}+k_{rISC1}n_{T1}$, (Eq. S5)

$\frac{{dn}_{T2}}{dt}=k_{\mathrm{ISC}}n_{S1}-k_{r\mathrm{ISC}2}n_{T2}-k_{IC}n_{T2}+k_{rIC}n_{T1}$, (Eq. S6)

$\frac{{dn}_{T1}}{dt}=-\frac{n_{T1}}{\tau_{T1}}+k_{IC}n_{T2}-k_{rIC}n_{T1}-k_{rISC1}n_{T1}-k_{TTA}n_{T1}^{2}$. (Eq. S7)

In above equations, the rate *k*_RISC1_ is the rISC rate from T_1_ to S_1_, *k*_RISC2_ the rISC rate from T_2_ to S_1_, *k*_IC t_he rate of internal conversion from T_2_ to T_1_, and *k*_rIC_ the rate of reverse internal conversion from T_1_ to T_2_.


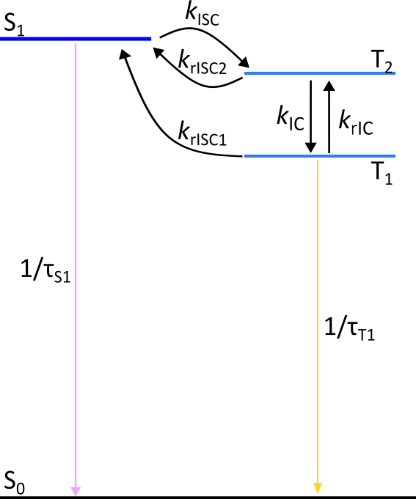


**Fig. S24** A comprehensive four-level model. The ISC processes from S_1_ to T_2_ and T_1_ have been considered, with also the consideration of rISC process from T_1_ to S_1_, and the rIC process from T_1_ to T_2_. Moreover, the internal transitions between T_2_ and T_1_ have been included as well.

Thermal up-conversion of larger barriers than the 'thermal energy' is possible due to the Boltzman distribution of different triplet states and while the rates follow the Arrhenius equation. We therefore further assume that the rISC from T_1_ to S_1_, and the rIC rate from T_1_ to T_2_ might follow the Arrhenius equation:

$\frac{k_{rISC1}}{k_{rISC2}}=exp\left( \frac{-\Delta E_{T2T1}}{k_{B}T} \right)$, (Eq. S8)

$\frac{k_{\mathrm{rIC}}}{k_{\mathrm{IC}}}=exp\left( \frac{-\Delta E_{T2T1}}{k_{B}T} \right)$. (Eq. S9)

in which Δ*E*_T2T1_ is the energy difference between T_2_ and T_1_.

Experimentally, as summarized in **Table S6**, when the energy difference between S_1_ and T_1_ is estimated from the emissive peak, it is about 0.2 eV. The energy levels determined by different methods are summarized in **Table S6**. There is a slight difference of energy levels in the range ~100 meV when determining from emissive peaks or lineshape analysis. Meanwhile, it might be some simulation deviations around 0.1-0.5 eV from the experimental values of the energy level for the excited state. We therefore did not use a fixed Δ*E*_T2T1_ to fit our transient PL decay, but with a range of different values to check its impact in the following discussion.


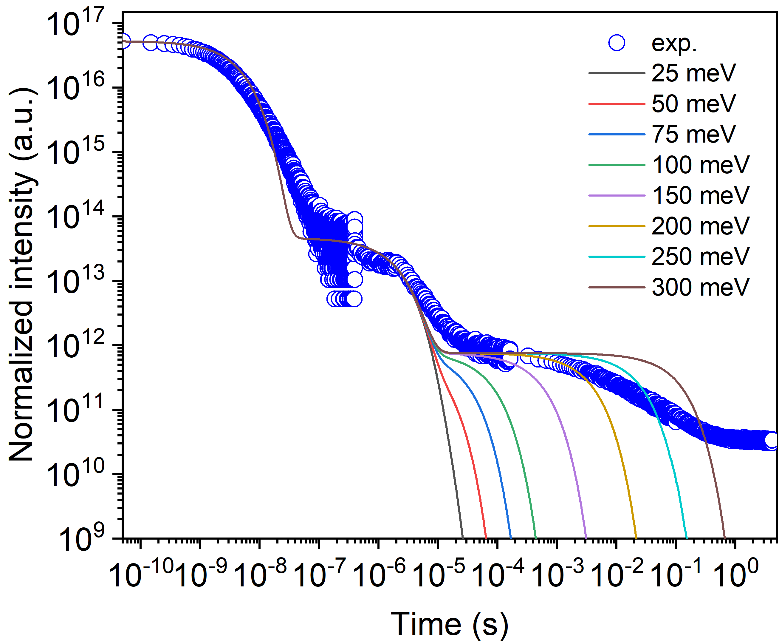


**Fig. S25** Numerical fitting of the experimental transient PL decay with the consideration of rISC from T_1_ to S_1_, and rIC from T_1_ to T_2_.

Furthermore, the TTA effect (*k*_TTA_ = 0) is neglected to check the impact of energy difference between T_2_ and T_1_. Therefore, for the fitting based on the more comprehensive four-level model, the following parameters have been adopted: τ_S1_=6 ns, *k*_ISC_ =6.0×10^7^ s^-1^, *k*_rISC2_ =7.5×10^5^ s^-1^, *k*_B_T = 25.6 meV. Since the TTA effect can even suppress the effective lifetime of phosphorescence, such a numerical model can estimate the energy difference and therefore the rates of rISC2 and rIC.

As shown in **Fig. S25**, with a lower energy difference, such as the 50-100 meV, the k_RISC1_ is about 0.02-0.2 *k*_rISC2_, which is around 1.5×10^4^ -1.5×10^5^ s^-1^. According to Eq.S7, in these cases, the effective lifetime of phosphorescence is only 10-100 μs, many orders smaller than the experimental results. To obtain the phosphorescent decay with a decay lifetime of hundreds of millisecond, the energy difference between T_2_ and T_1_ should be ~250-300 meV under the given assumption (Eq. S8 and S9).

Experimentally, as shown in **Fig. S26**, we can obtain almost identical long-lived decay for the model emitter in PMMA host. Therefore, the experimentally measured phosphorescence lifetime is ~120 ms, indicating that the maximum rISC from T_1_ to S_1_, and rIC rate from T_1_ to T_2_ would be only ~8 s^-1^. Therefore, the rISC rate from T_1_ to S_1_ is many orders of magnitude smaller than the rISC rate from T_2_ to S_1_, which can be dropped to simplify the model.


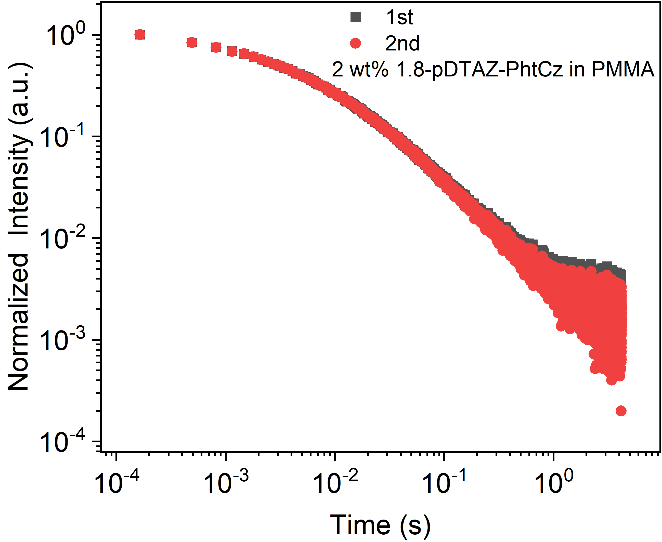


**Fig. S26** Cross check of the phosphorescence decay at room temperature.

Therefore, similar effect can be found between the ratio of rIC and IC. With such an energy difference under the condition of fast equilibrium, rIC rate is also much slower than the IC rate as well. Furthermore, the estimated IC rate is only around 30 s^-1^, orders of magnitude smaller than the rISC rate from T_2_ to S_1_.

# 5. Transient absorption spectroscopy


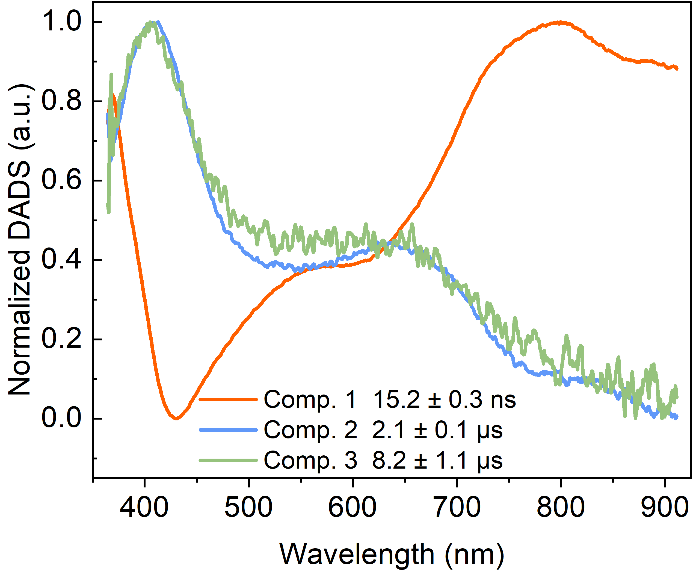


**Fig. S27** Normalized DADS for global analysis with a parallel model for 3 components of the model emitter 1.8-mDTAZ-PhtCz.


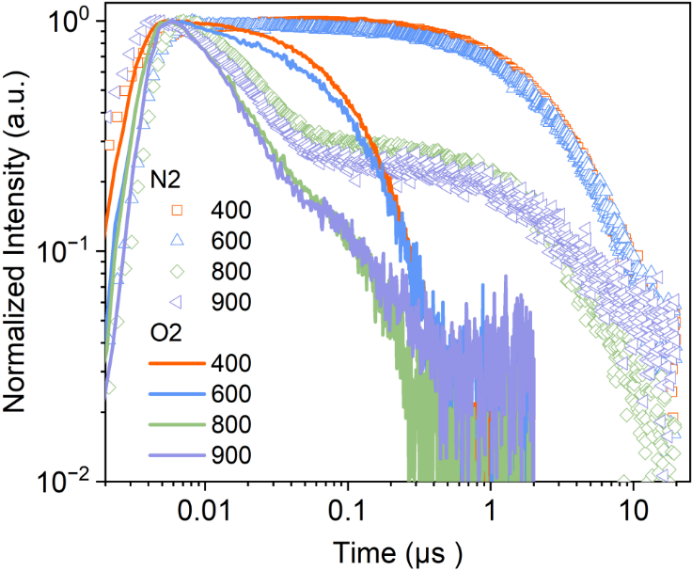


**Fig. S28** Oxygen sensitivity of ns-TA in degassed and aerated solutions of the model emitter 1.8-mDTAZ-PhtCz.


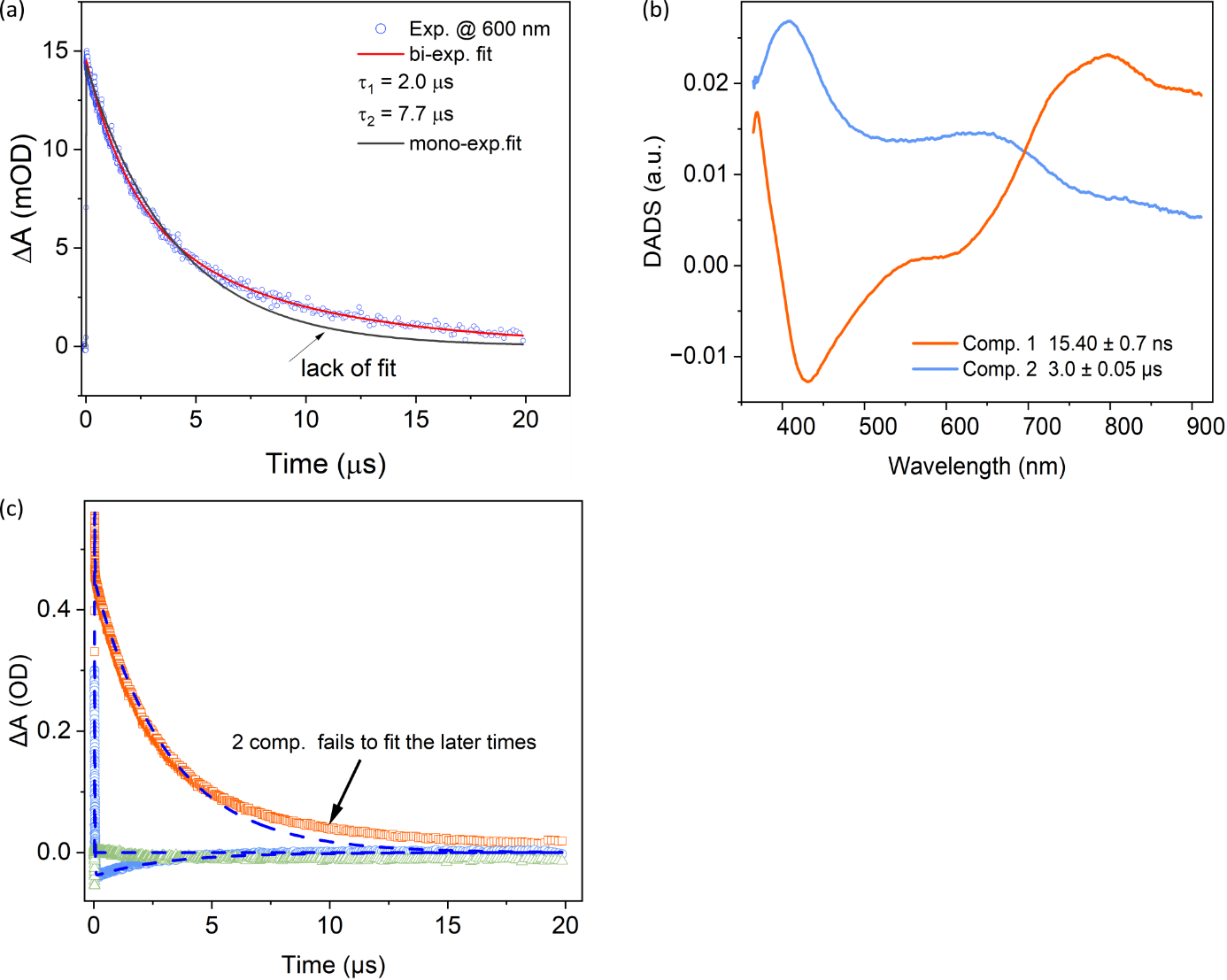


**Fig. S29** Global analysis with a parallel model for two components of 1.8-mDTAZ-PhtCz. (a) Lack of fit with a bi-exponential fitting at 600 nm; (b) DADS; (c) Experimental and fitted kinetics, in which the long-lived signals are failed to fit.


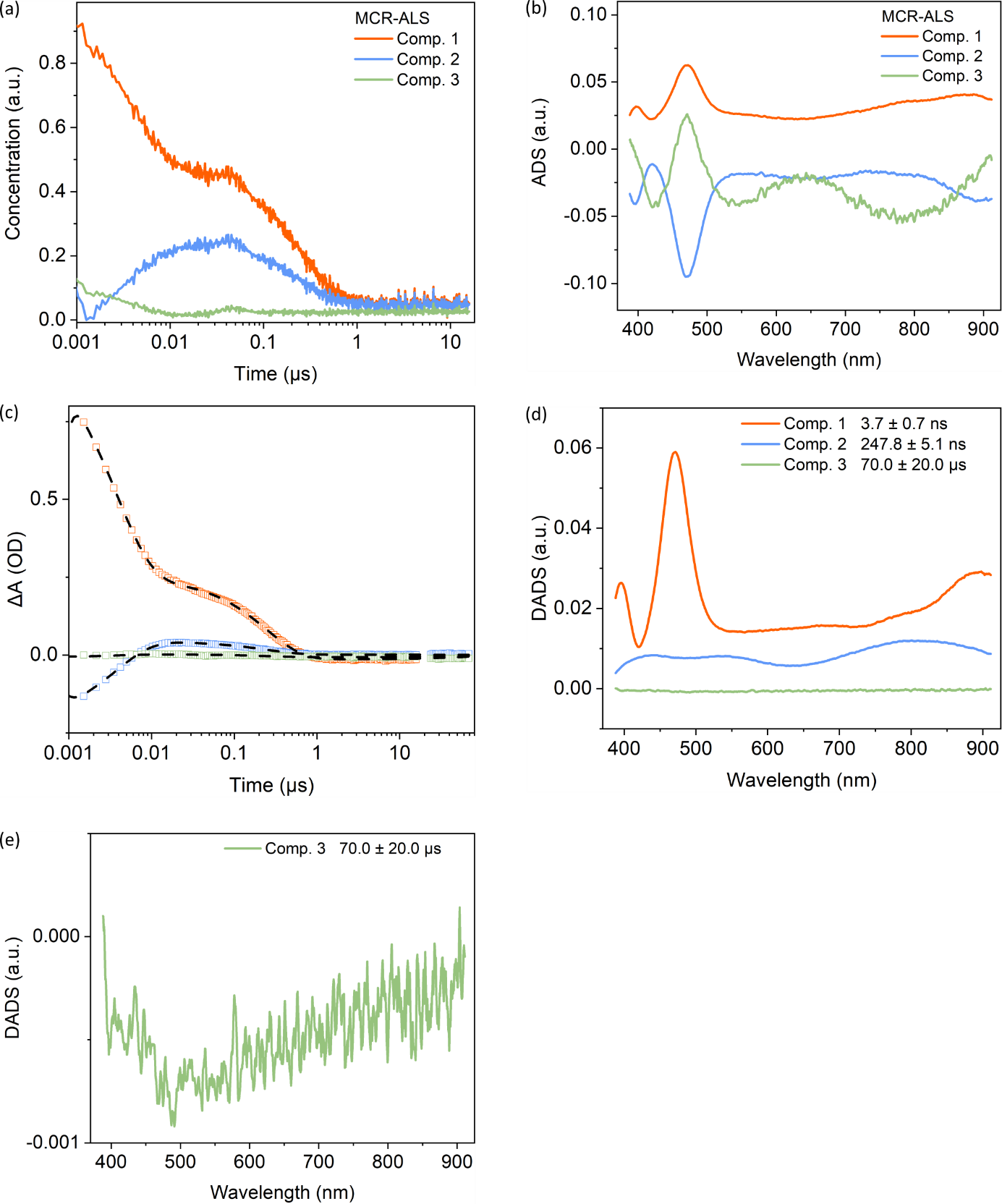


**Fig. S30** Experimental ns-TA spectra of 1.8-pDTAZ-PhtCz in degassed toluene. (a) Concentration evolution and (b) associated spectra of the principal components from MCR-ALS fitting. (c) Kinetic fitting by the global analysis with three components based on a parallel scheme. (d) DADS from the global analysis. (e) A closer look of the DADS for the slowest component shown in (d).


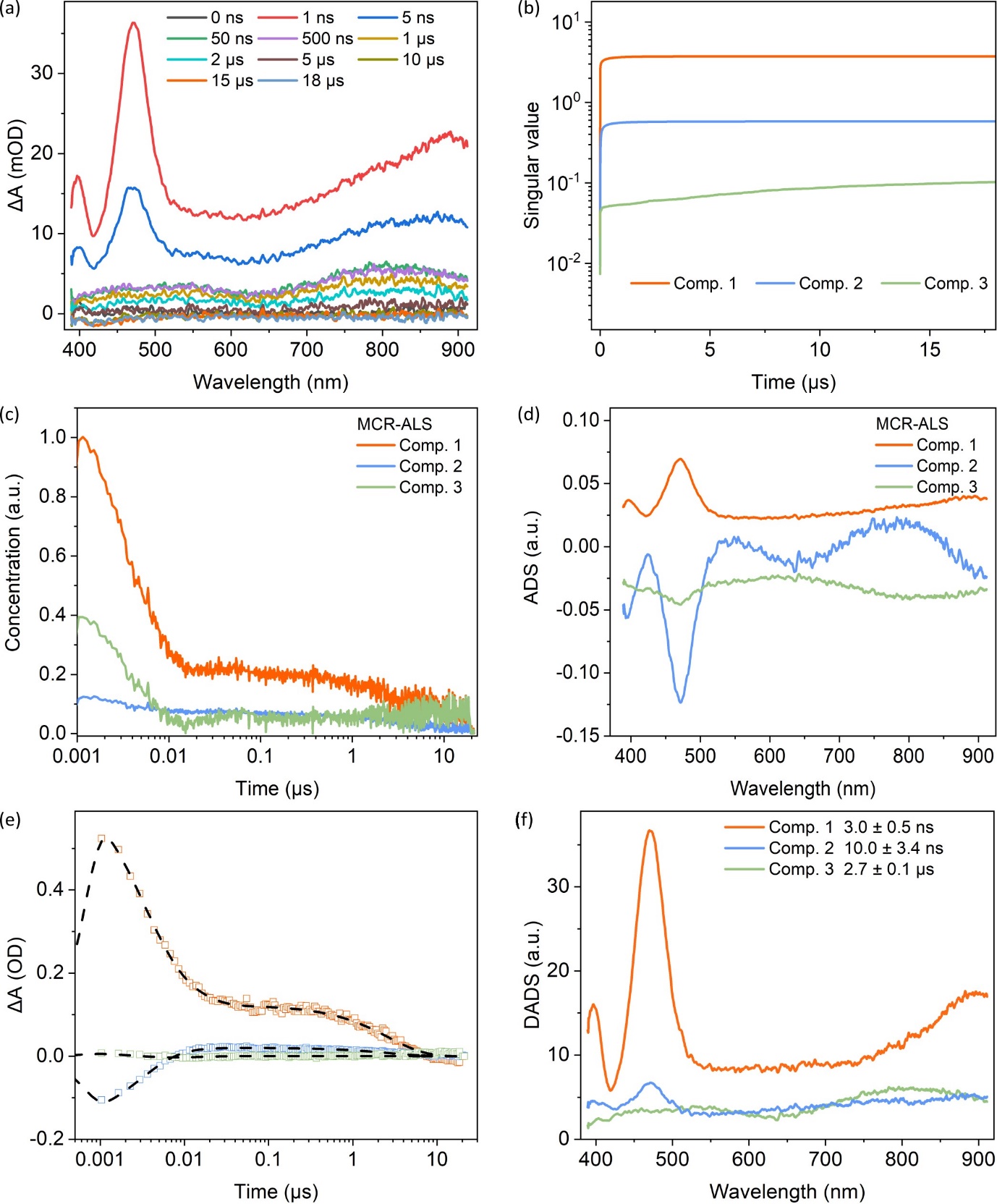


**Fig. S31** Experimental ns-TA spectra of 1.8-pDTAZ-PhtCz in aerated toluene with oxygen quenching. (a) Specific ns-TA spectra in the UV-Vis range. (b) Forward EFA of the ns-TA data. (c) Concentration evolution and (d) Associated spectra of the principal components from MCR-ALS fitting. (e) Kinetic fitting by the global analysis with 3 components based on a parallel scheme. (f) DADS from the global analysis.


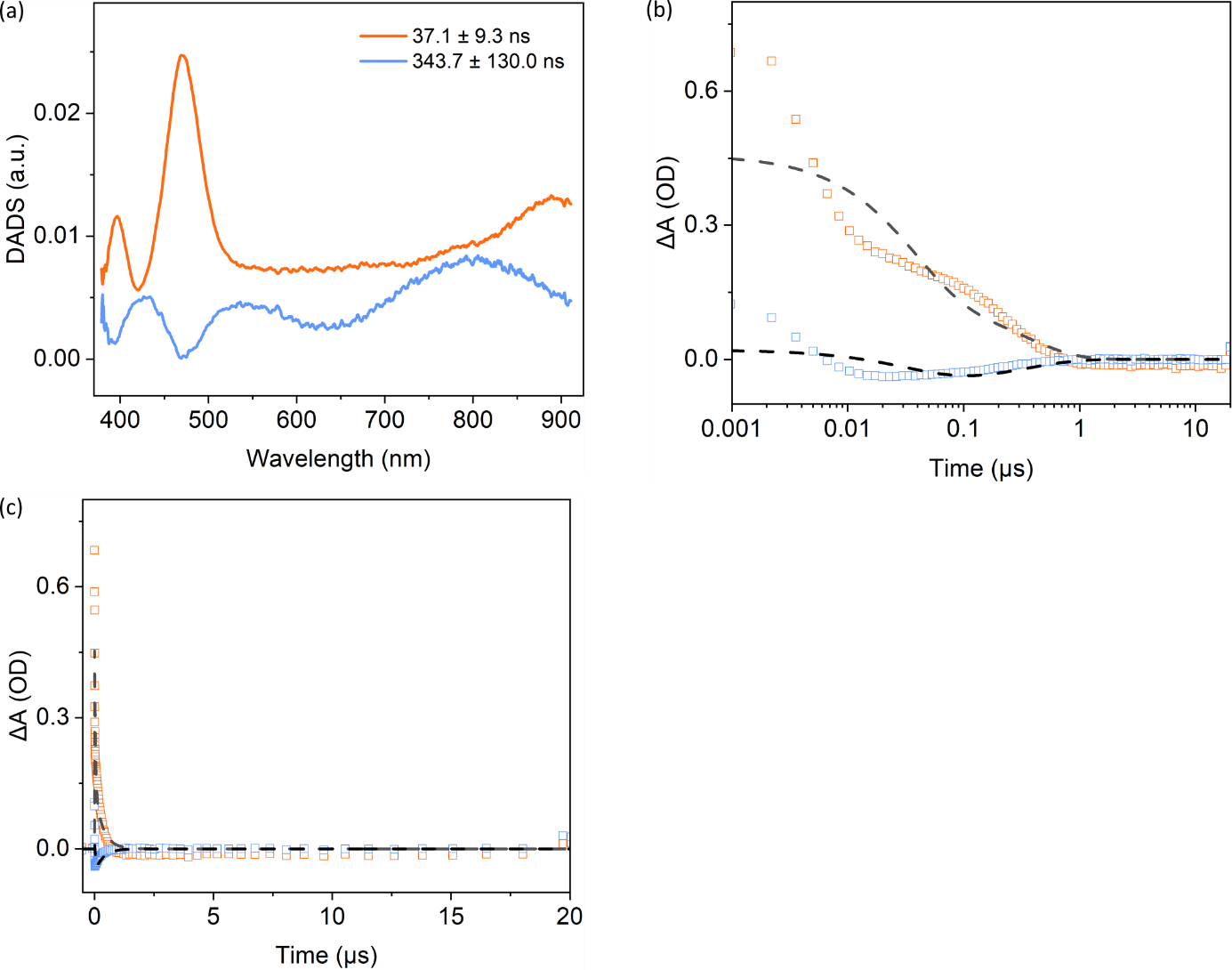


**Fig. S32** Global analysis of the experimental ns-TA spectra of 1.8-pDTAZ-PhtCz in aerated toluene with two components based on the parallel scheme. (a) DADS from the global analysis with the assumption of two components involvement. Lack of fit for the kinetic decay fitting is observed both in the ns region (b) and μs region (c) in this case.


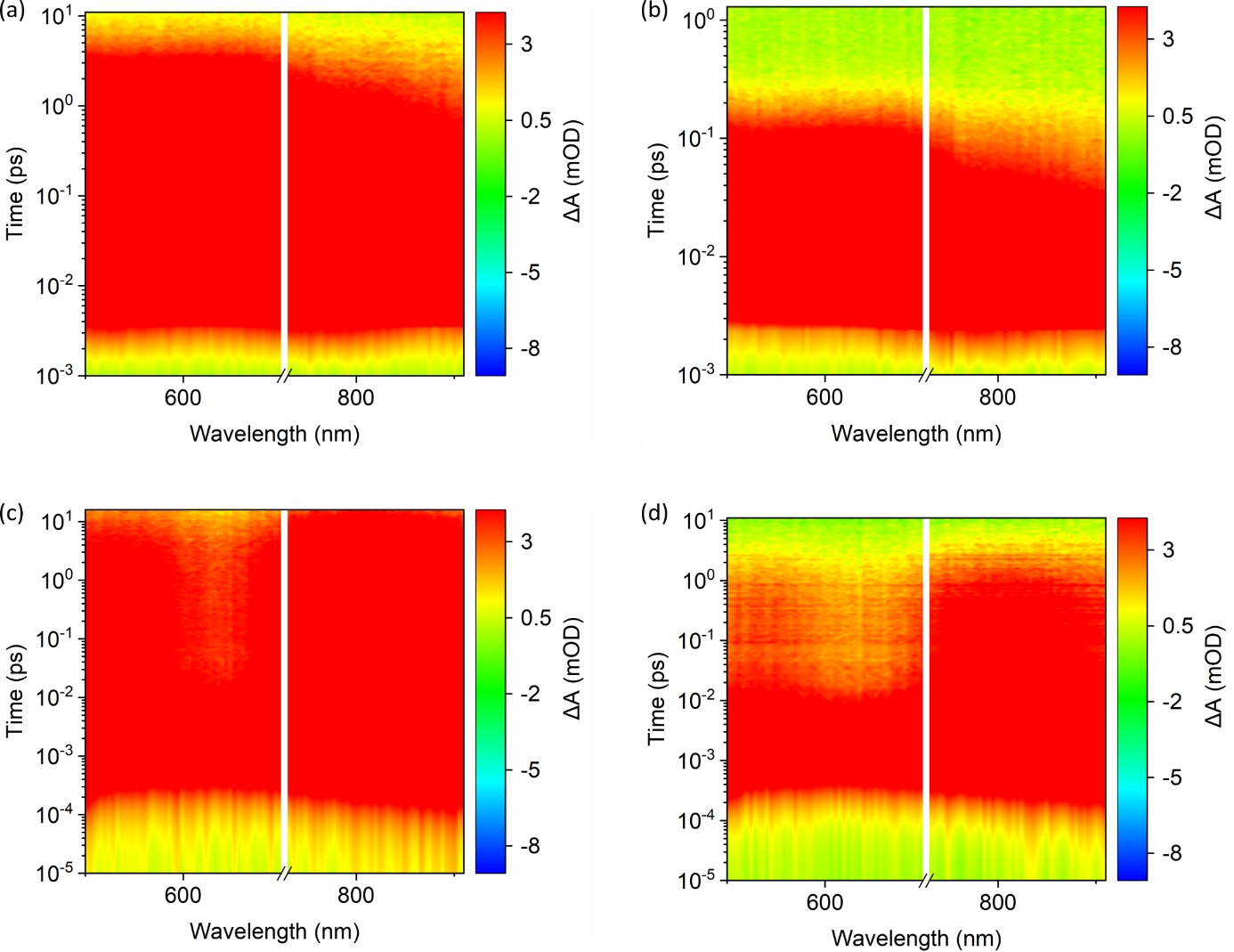


**Fig. S33** The 2D ns-TA spectra in toluene. (a) and (b) show molecules 1.8-mDTAZ-PhtCz in the degassed and aerated, respectively. (c) and (d) show molecules 1.8-pDTAZ-PhtCz in the degassed and aerated, respectively.

# 6. DFT calculations


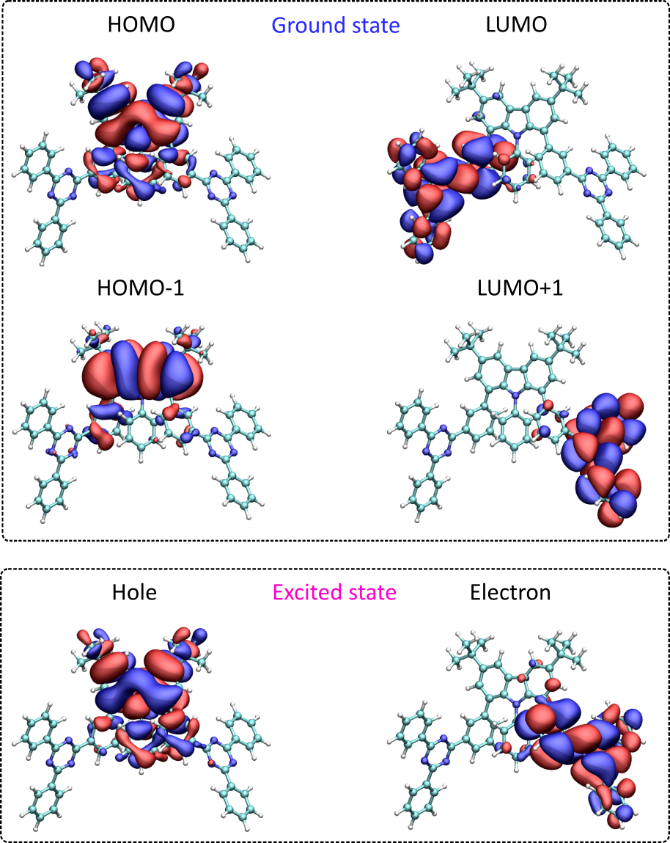


**Fig. S34** Ground state and excited state electron distributions based on restricted ROKS for the model emitter.

**Table S9** ROKS, DFT and TD-DFT simulation results for the model emitter (1.8-mDTAZ-PhtCz).

|  | ROKS | B3LYP | ωT-CAM-B3LYP | ωT-ωB97xd |
| --- | --- | --- | --- | --- |
| Absorption | 3.493 eV | 3.087 eV | 3.099 eV | 3.180 eV |
| S_1_ | 2.978 eV | 2.700 eV | 2.604 eV | 2.650 eV |
| T_2_ | 2.953 eV | 2.790 eV | 2.768 eV | 2.820 eV |
| T_1_ | 2.912 eV | 2.613 eV | 2.585 eV | 2.634 eV |

# 7. Multi-color FRET systems

**
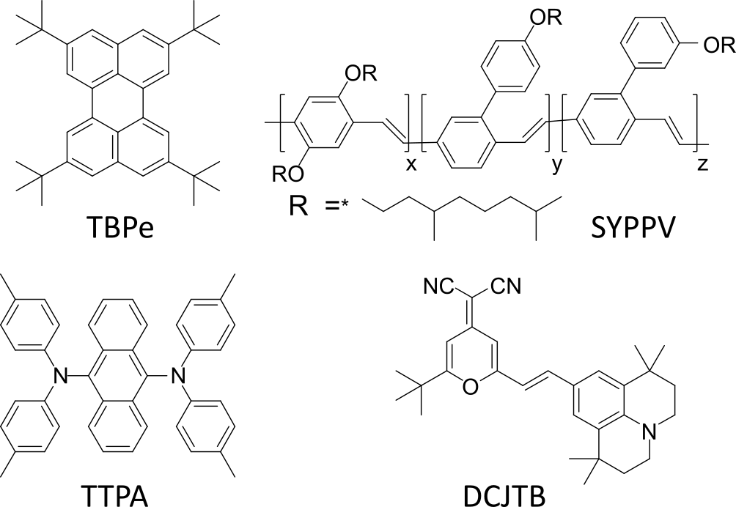
**

**Fig. S35** Chemical structures of conventional fluorescent dopants.


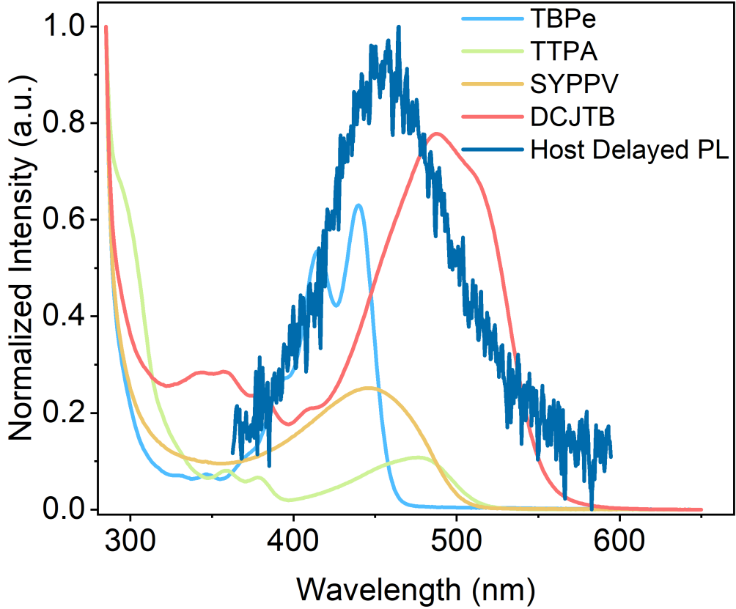


**Fig. S36** Spectra overlap between the dopant absorption and delayed spectra of 1.8-mDTAZ-PhtCz.


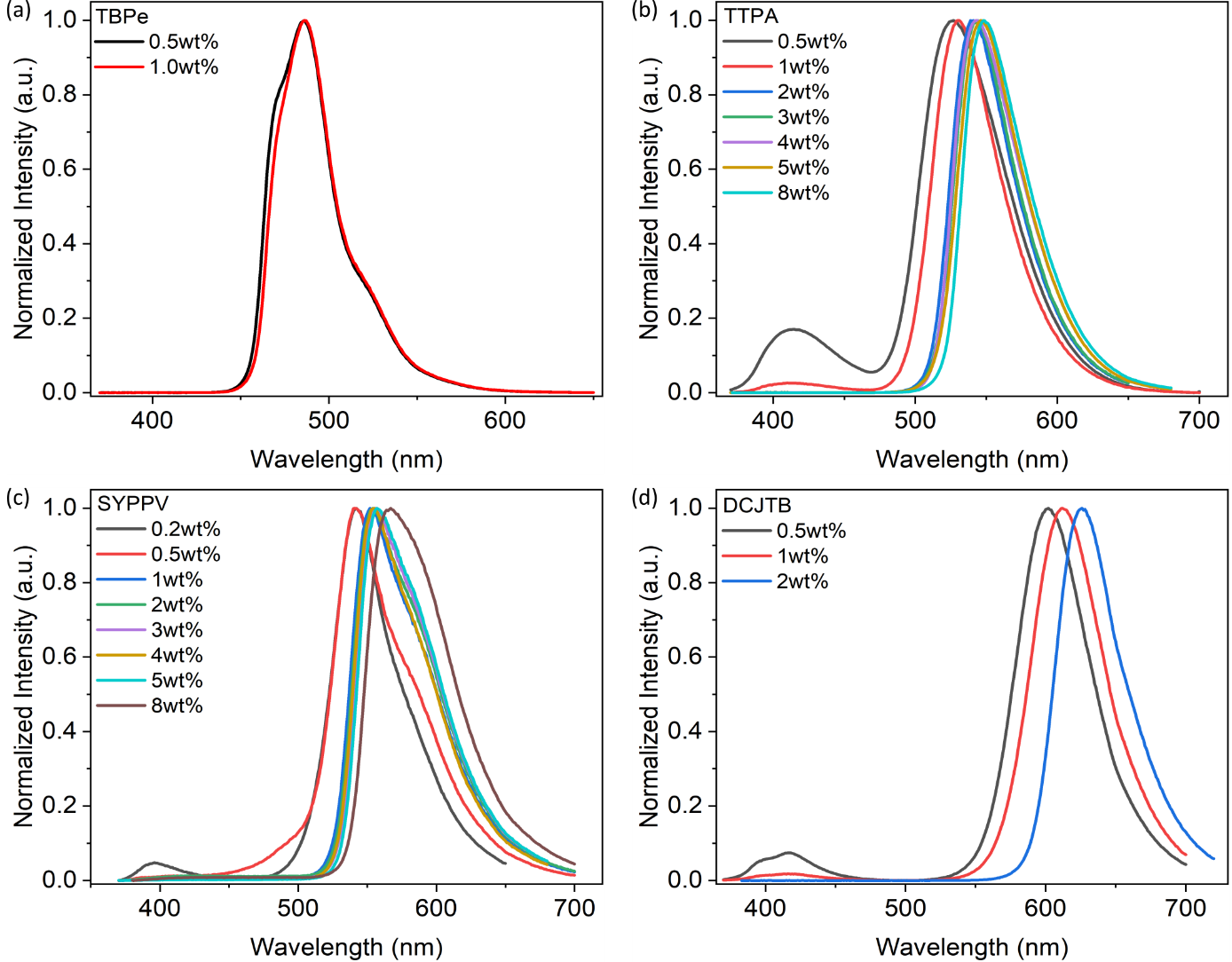


**Fig. S37** Steady state fluorescence spectra with conventional fluorescent emitters doped in PMMA. (a) TBPe; (b) TTPA; (c) SYPPV; (d) DCJTB. Fluorescent emitters are doped in PMMA with a concentration as PMMA: 1.8-mDTAZ-PhtCz: dopant = 1: 2wt%: x wt%, with the concentration of the conventional fluorescent emitter indicated in the legend.


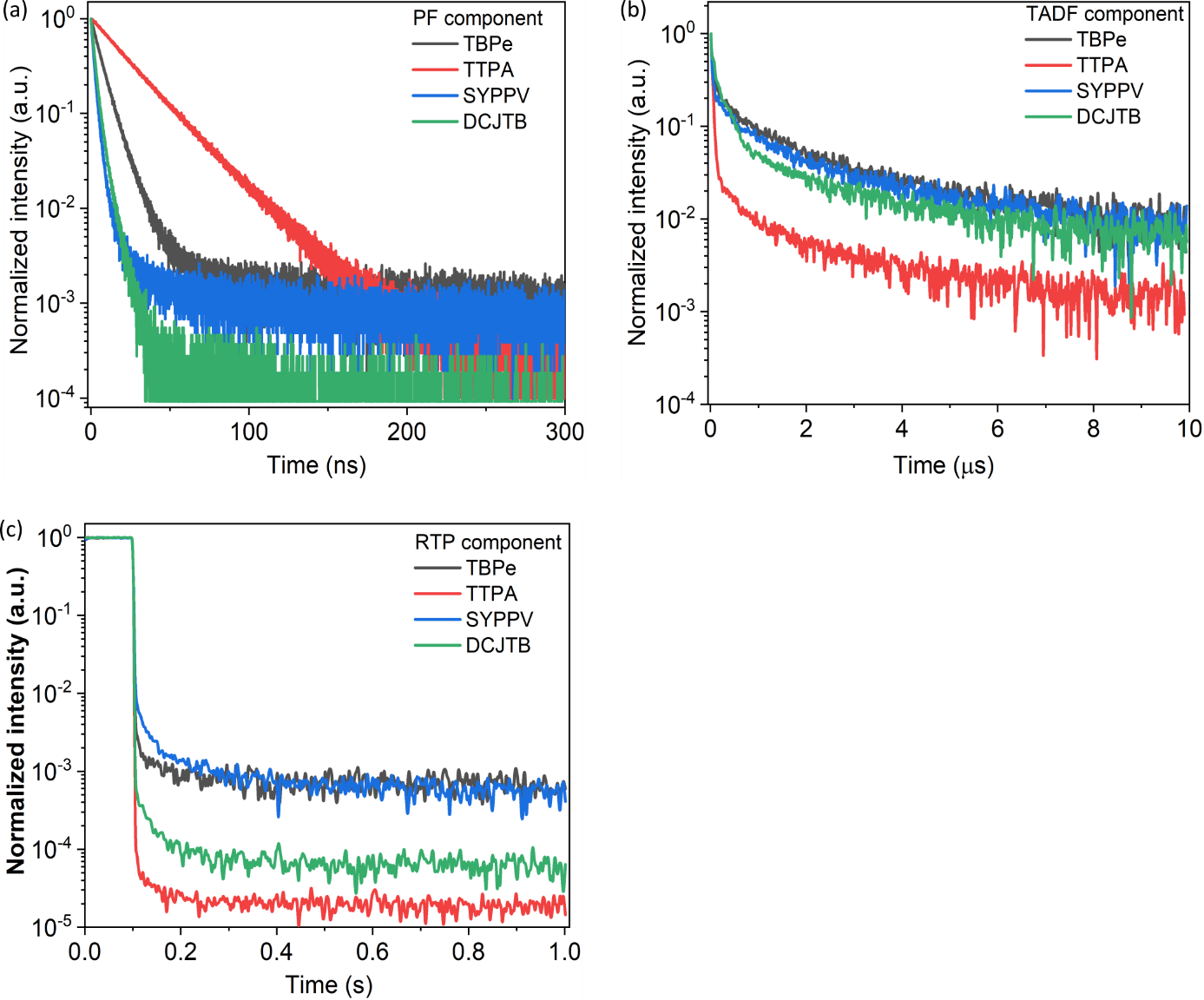


**Fig. S38** Multi-color PF-TADF-RTP emission via FRET from nanosecond to second regions. (a) PF component in ns region. (b) TADF component in μs region. (c) RTP component in ms region.


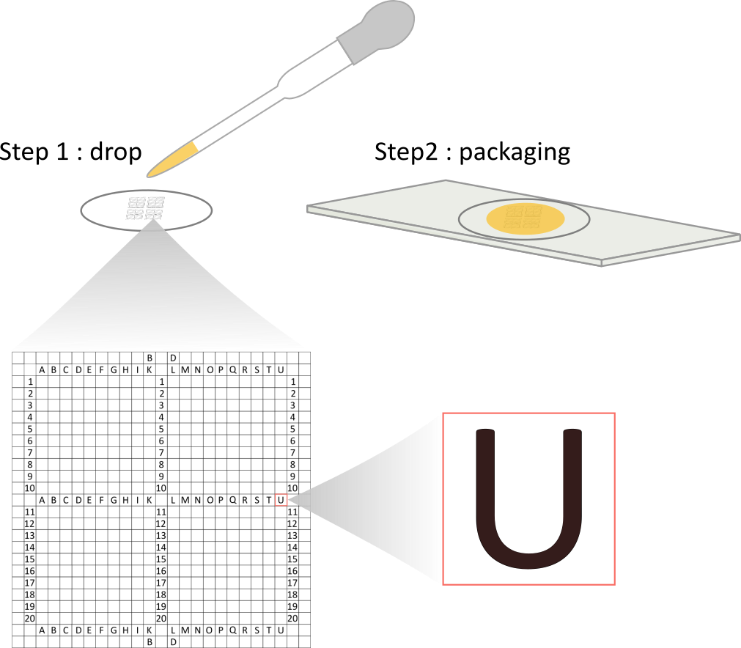


**Fig. S39** Schematic presentation of the preparation of microscopic imaging samples.


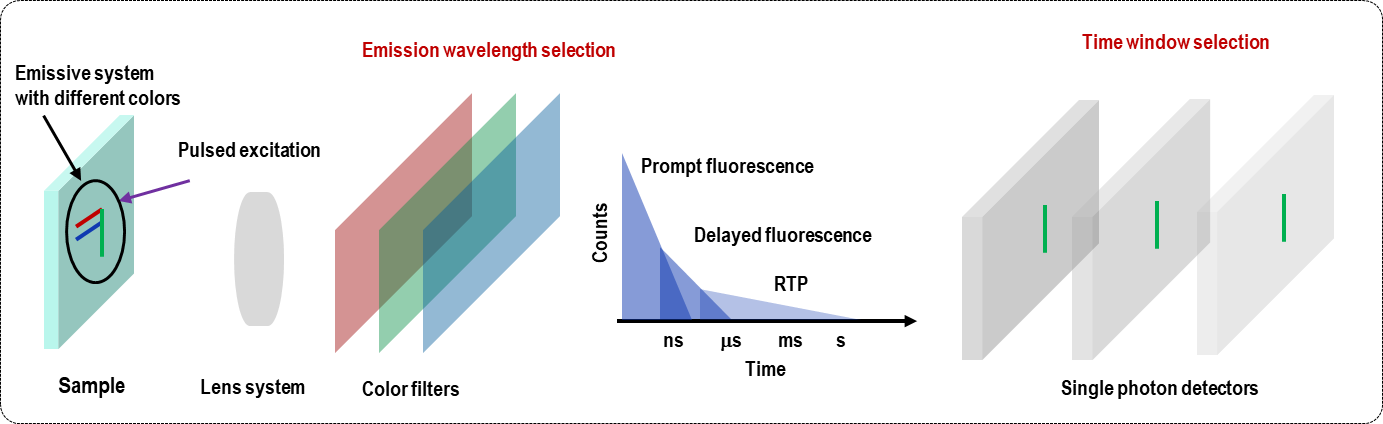


**Fig. S40** Schematic illustration on time-dependent and emissive wavelength-dependent microscopy imaging application.


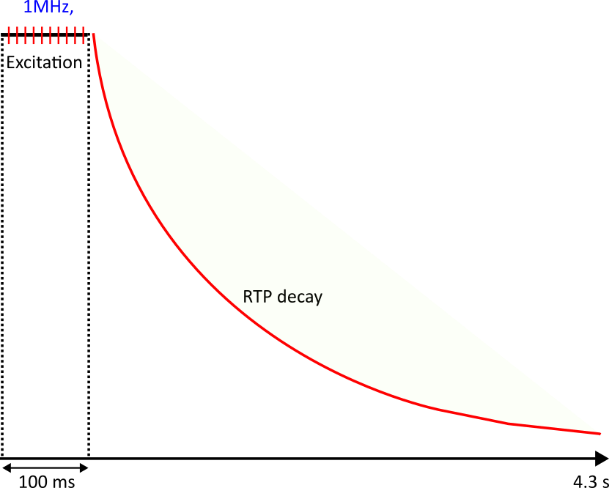


**Fig. S41** Schematic diagram of the RTP test.

To properly capture the long-lived phosphorescence signal in the millisecond region, we here still use the TCSPC method to measure the long-lived decay, by tuning the excitation pulses to burst mode. To accumulate a sufficient population of long-lived triplet states, excited with 1 MHz laser for 100 ms, and then the excitation is stopped for ~4 s, during which time region we can collect the histogram of number of photons as a function arrival time spanning from 0-4s. If there is no long-lived emissive triplets, e.g. in the degassed toluene of the model emitter in **Fig. S9**, no long-lived signal will be detected. In other words, for three-level systems with only a PF and DF component, such a measurement method can avoid the potential artifact due to a large gate time in TRPL measurements with an iCCD to collect the weak signal in the tail part.

**Table S10** The RTP parameters of reported organic emitters without heavy atoms based on carbazole as the donor.

| Emitters | RTP lifetime | Phosphorescence quantum yield | Wavelength  for RTP decay | Reference |
| --- | --- | --- | --- | --- |
| Cz-DBF | 650 ms | 14.3% | 550 nm | Nat. Commun. **10**, 1595 (2019) |
| DPhCzT | 1.06 s | 1.25% | 530 nm | Nat. Mater. **14**, 685–690 (2015) |
| DECzT | 1.28 s | 0.6 % | 529 nm |  |
| 4-BACz | 558 ms | 6.9% | 549 nm | J. Mater. Chem. C. **7**,9095–9101 (2019) |
| CzBP | 518 ms (in crystal) | 1.4% | 570 nm | Adv. Mater. **27**, 6195–6201 (2015) |
| CBA | 540 ms | n.a | 550 nm | Chem.Commun. **51**,10381–10384 (2015) |
| CPM | 748 ms | 3.17% | 530 nm | Adv. Mater. **29**, 1606829 (2017) |
| m-MCBA | 795 ms | 2.1 % | 600 nm | Angew. Chemie - Int. Ed. **57**, 7997–8001 (2018) |
| TCz-F | 727 ms | 7.4 % | 556 nm | Nat. Commun. **11**, 2617 (2020) |
| PhCz | 646 ms | 0.7 % | 530 nm | Adv. Mater. **29**, 1701244 (2017) |
| m-PBCM | 710 ms  (in recrystallized solids) | 5.7 % | n.a. | Adv. Mater. **31**, 1807222 (2019) |
| p-PBCM | 311 ms  (in recrystallized solids) | 9.8 % | n.a. |  |
| o-PBCM | 344ms  (in recrystallized solids) | 10.2 % | n.a. |  |
| CX | 150 ms (in crystal) | n.a. | n.a. | Chem. Sci. **9**, 3782–3787 (2018) |
| PTCz | 810 ms (in crystal) | 5.1 % | n.a. | Adv. Opt. Mater. **9**, 2001549 (2021) |
| CzBz | 321 ms | 2.18 % | n.a. | J. Phys. Chem. C. **125**, 16350–16357 (2021). |
| 1.8-pDTAZ-PhtCz | 118.7 ms | 33.6 % | 523 nm | This work |
